# Dealkylation as a Strategy to Synthesize Unconventional Lithium Salts from *ortho*-Phenyl-phosphonate-boranes

**DOI:** 10.1021/acs.inorgchem.5c05101

**Published:** 2026-01-13

**Authors:** Anthony D. Kornokovich, Arnold L. Rheingold, Vallabha R. Rikka, Wan Si Tang, Judith A. Jeevarajan, John D. Protasiewicz

**Affiliations:** # Department of Chemistry, 2546 Case Western Reserve University, 2080 Adelbert Road, Cleveland, Ohio 44106, United States; ‡ Department of Chemistry and Biochemistry, 8784 University of California, San Diego, La Jolla, California 92093, United States; § Electrochemical Safety Research Institute, 630960 UL Research Institutes, 5000 Gulf Freeway, Houston, Texas 77204, United States

## Abstract

Several *ortho*-phenyl-phosphonate-boranes 1-BR_2_-2-{P­(O)­(OEt)_2_}­C_6_H_4_ (R = Cy (cyclohexyl, **2a**), Ipc ((+)-isopinocampheyl, **2b**), and ^n^Hx (*n*-hexyl, **2c**)) have been prepared and characterized. Compounds **2a**–**c** can be selectively mono-dealkylated to afford the corresponding lithium *ortho*-phenyl-borato­phosphonate salts [Li­(S)_*n*_]­[1-BR_2_-2-{P­(O)_2_(OEt)}­C_6_H_4_] (S = MeCN or EtOAc). All compounds were characterized by multinuclear NMR spectroscopy (^1^H, ^13^C­{^1^H}, and ^31^P­{^1^H}). Reactions of **2a** with NaI or KI yielded the respective [Na­(MeCN)]­[1-BCy_2_-2-{P­(O)_2_(OEt)}­C_6_H_4_] ([Na­(MeCN)]­[**3**]) and [K­(MeCN)]­[1-BCy_2_-2-{P­(O)_2_(OEt)}­C_6_H_4_] ([K­(MeCN)]­[**3**]) salts. Single-crystal X-ray diffraction studies of **2a** and [Li­(MeCN)_2_]­[1-BCy_2_-2-{P­(O)_2_(OEt)}­C_6_H_4_] ([Li­(MeCN)_2_]­[**3**]) document the presence of intramolecular P=O···B interactions that form pseudo-heterocyclic rings. In the solid state, [Li­(MeCN)_2_]­[**3**] exists as a lithium-bridged dimer. Thermogravimetric analysis of [Li­(MeCN)_2_]­[**3**] reveals high thermal stability with a decomposition onset above 200 °C. [Li­(MeCN)_2_]­[**3**] displays good to excellent solubility in many organic solvents. Reactions of [Li­(MeCN)_2_]­[**3**] with chlorotrimethylsilane, methyl triflate, and HCl·Et_2_O yielded the new *ortho*-phenyl-phosphonate-boranes 1-BCy_2_-2-{P­(O)­(OE)­(OEt)}­C_6_H_4_ (E = Me_3_Si, Me, H). Compounds **2a** and [Li­(MeCN)_2_]­[**3**] are weakly emissive in solution and in the solid state, with evidence of solvent-dependent dual emission.

## Graphical abstract

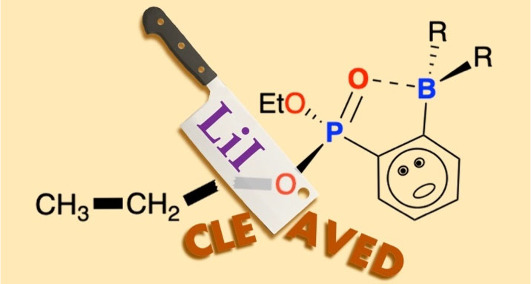

The design of new main group anions, especially weakly coordinating anions (**WCAs**), has become an important area of inorganic chemistry, showing relevance across various inorganic applications, including electrolyte systems,^1^ catalyst activation,^2^ and the stabilization of reactive main group cations.^3^ Through careful selection of organic substituents about the central atom, the electrostatic Coulombic attractions to counterions can be modulated, high oxidative and electrophilic stability can be imparted, and good solubility in organic media can be achieved.^4,5^

Early examples of **WCAs** include small, fluorinated anions such as [PF_6_]^−^, [BF_4_]^−^, and [NTf_2_]^−^.^6^ Since then, research has focused on the development of larger, more sterically restricted anions tailored to further reduce coordinating interactions.^7^ Representative examples include fluorinated borates such as [B­(C_6_F_5_)_4_]^−^ and [B­(Ar^CF^_3_)_4_]^−^ (Ar^CF^_3_ = 3,5-(CF_3_)_2_C_6_H_3_) as well as alkoxy­aluminates such as the Krossing anion, [Al­(OR^F^)_4_]^−^ (R^F^ = C­(CF_3_)_3_).^6,8^ Examples of **WCAs** are also being explored for potential use as counterions for electrolyte salts in energy storage applications such as lithium ion batteries (**LIBs**).^8,9^

Our group’s ongoing interest is in the design and synthesis of custom anions that impart flame-retardant characteristics to lithium salts. Our strategy toward such bifunctional flame-retardant ions (**FRIONs**) has involved searching for new lithium salts that integrate phosphorus­(V) centers into the anion that may promote char formation.^10–12^ Preliminary studies have revealed good thermal and electrochemical stability as additives in **LIB** liquid electrolyte formulations. However, previous **FRIONs** examined thus far suffered from limited solubility in organic solvents, in particular, alkyl carbonate solutions, which limits their application in **LIB** chemistry. Common to many **WCAs** and our previous **FRIONs** is the existence of one or more elements of molecular symmetry. Recognizing, as others have,^13,14^ that the solubility of battery constituent compounds and salts can be enhanced by intentionally lowering the molecular symmetry (Carnelley’s rule^15^), we set out to prepare examples of low-symmetry lithium borate salts that would also feature P­(V) centers to enhance the propensity to undergo char formation during combustion in air.

Herein we report on the synthesis and characterization of lithium *ortho*-phenyl-borato­phosphonate salts with the general formula [Li­(S)_*n*_]­[1-BR_2_-2-{P­(O)_2_(OEt)}­C_6_H_4_] (S = solvent) that are readily prepared by LiI-promoted dealkylation reactions of the neutral precursors 1-BR_2_-2-{P­(O)­(OEt)_2_}­C_6_H_4_.

Reactions of (*ortho*-bromophenyl)­phosphines with RLi, followed by electrophilic quenching with R_2_BCl to afford the corresponding *ortho*-aryl-phosphinoboranes, are well established.^16,17^ *ortho*-Aryl-phosphinoboranes represent an interesting class of compounds, as the close proximity of a phosphine donor site to a boron acceptor site gives rise to potential Frustrated Lewis Pair (**FLP**) chemistry.^16,18^ *ortho*-Phenyl-phosphinoboranes can stabilize highly reactive zwitterionic intermediates through diethyl azodicarboxylate and PhNCO reactions.^19–21^ Ample precedent thus provides a convenient entryway to *ortho*-phenyl-phosphonate-boranes **2a**–**c** from **1** (Scheme 1). For example, the reaction of compound **1** ^22^ with ^i^PrLi at −78 °C followed by the addition of Cy_2_BCl, affords 1-BCy_2_-2-{P­(O)­(OEt)_2_}­C_6_H_4_ (**2a**). Compounds **2a** and **2b** were isolated as crystalline solids in 63.5% and 25.4% yields, respectively. Compound **2c** was isolated as an oil in 67.7% yield. All three compounds displayed diagnostic ^31^P­{^1^H} NMR resonances (δ = 42.7, **2a**, δ = 40.7, **2b**, δ = 44.1, **2c**) significantly shifted downfield from the shift displayed for **1** (δ = 14.8), consistent with formation of an intramolecular P=O···B interaction.^23^

**Scheme 1:**
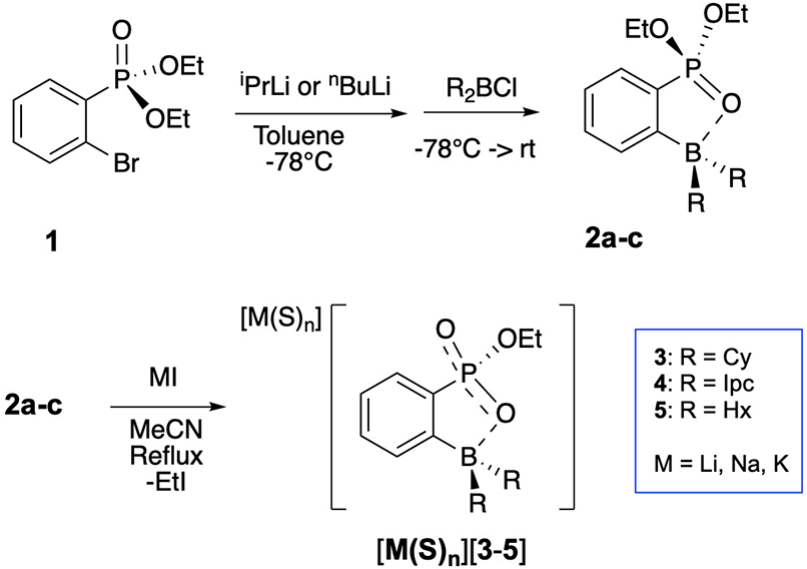
Generalized Route to [M­(S)_*n*_]­[1-BR_2_-2-{P­(O)_2_(OEt)}­C_6_H_4_] Salts

Reactions of phosphorus esters with alkali metal halides (MX) to exchange hydrocarbyl groups with alkali metals and extrude RX are well established.^24–26^ Heating mixtures of **2a** with a slight excess of LiI (1.2 equiv) under reflux for 26 h produces a white precipitate, identified as [Li­(MeCN)_2_]­[**3**] (Scheme 1). This material was isolated in 59.3% yield as an analytically pure, free-flowing solid after filtration and drying under reduced pressure. Extended drying under high vacuum, however, can remove one of the two molecules of MeCN (by ^1^H NMR spectroscopy). Reaction of **2b** with LiI similarly generated lithium salt [Li]­[**4**], but upon workup and purification by passing the reaction mixture through silica gel using EtOAc, the [Li­(EtOAc)_3_]­[**4**] salt was ultimately isolated as a colorless oil in 36.9% yield. Reaction of LiI with **2c** led to isolation of a yellow oil in ca. 77% yield, for which NMR spectroscopy is consistent with the formulation as [Li­(MeCN)]­[**5**]. We have not yet isolated [Li­(MeCN)]­[**5**] in its pure form. All three salts display ^31^P­{^1^Η} NMR chemical shifts between δ 31.6 and 32.9 ppm in DMSO-*d*_6_. Two resonances for [Li­(EtOAc)_3_]­[**4**] are observed at 31.9 and 31.6 ppm, consistent with the expectation that two diastereomers can be distinguished by NMR spectroscopy after the addition of the new chiral center at phosphorus to the existing homochiral (+)-isopinocampheyl units at boron. The greater generality of the salt-forming protocol was demonstrated in reactions of **2a** with NaI and KI, which yielded [Na­(MeCN)]­[**3**] and [K­(MeCN)]­[**3**], respectively, in good yields. The ^31^P­{^1^H} NMR spectra of [Na­(MeCN)]­[**3**] and [K­(MeCN)]­[**3**] are quite similar to those of the lithium analogues (δ = 33.0 and 32.9 ppm).

Crystals of **2a** and [Li­(MeCN)_2_]­[**3**] suitable for X-ray diffraction were grown from concentrated solutions of toluene and acetonitrile, respectively, at −20 °C. The results of the two diffraction studies are depicted in Figure 1. Each structure displays significant B···OP interactions, yielding a five-membered pseudo-heterocyclic ring. In the solid state, [Li­(MeCN)_2_]­[**3**] associates into a dimer having a pair of lithium cations bridging adjacent anions via Li···OP interactions. A crystallographically imposed inversion center in the center of the Li_2_O_2_ diamond core relates the two halves of the dimer. Each lithium completes a tetrahedral coordination environment by the addition of two MeCN molecules.

**Figure 1:**
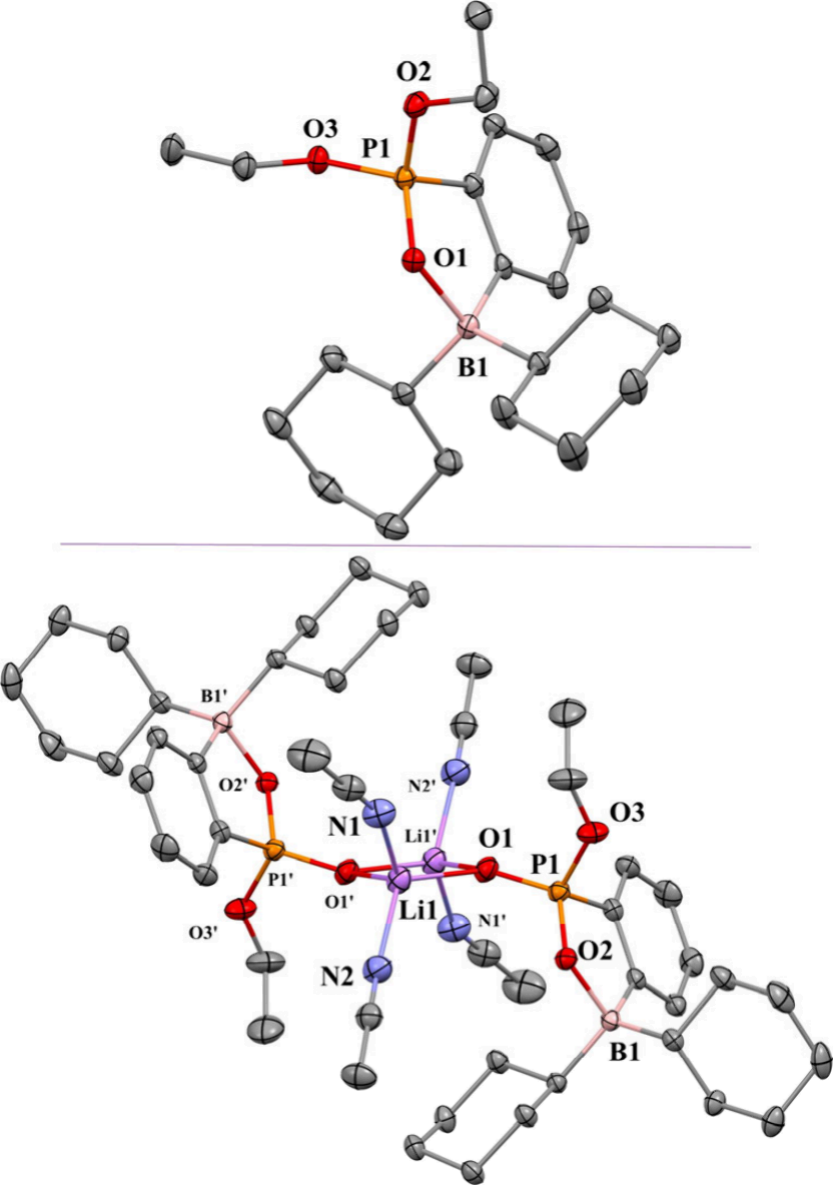
Thermal ellipsoid plots illustrating the 3D structures of compound **2a** (top) and [Li­(MeCN)_2_]­[**3**] (bottom) (hydrogen atoms omitted for clarity) in the solid state.

The B···OP distance of 1.666(2) Å in **2a** is longer than the corresponding 1.624(2) Å distance observed for [Li­(MeCN)_2_]­[**3**]. As the B···OP distances for the dative bond decrease, there is a corresponding increase in phosphorus–oxygen bond distances from 1.513(1) to 1.525(1) Å. These values fall within the range of other examples of B···OP dative bond distances determined for *ortho*-aryl-phosphinoborate oxides (from 1.463 to 1.643 Å).^23,27^ DFT calculations (B3LYP-D3­(BJ)/6-311++G­(2d,p)) on model neutral and anion compounds (Scheme 2) largely paralleled the changes in experimental EO (E = B or P) bond lengths. The fit of the computed to the experimental EO distances is better for the neutral species ***a***, as might be expected, for the calculation does not include the presence of Li ion coordination to the phosphoryl oxygen atoms. The greatest changes in PO bond distances are realized for the PO bond involving the oxygen atom from which an ethyl group was removed. The degree of pyramidalization at boron, as indicated by the sum of the CBC bond angles around boron, changes only 0.6°, consistent with marginal differences in the PO···B interactions between this pair of structures.

**Scheme 2:**
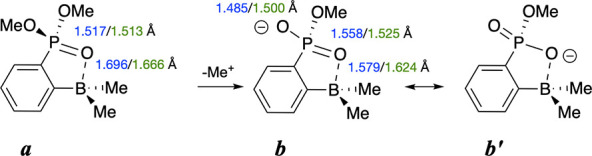
Calculated (Blue, Model R, R′ = Me) and Crystallographically Determined (Green, R = Et, R′ = Cy, **2a** and [Li­(MeCN)_2_]­[**3**] EO Bond Distances (E = P or B)

Preliminary reactions of [Li­(MeCN)_2_]­[**3**] with electrophilic reagents trimethylchlorosilane, methyl triflate, and HCl·Et_2_O led to new *ortho*-phenyl-phosphonate-boranes **6**, **7**, and **8**, respectively (Scheme 3). The ^31^P­{^1^H} chemical shifts for **6** (33.5 ppm), **7** (δ 42.6), and **8** (δ 36.6) compare favorably with those of the closely related **2a** (δ 42.7). These materials were all isolated as viscous oils, which presented difficulties in isolating them cleanly; nevertheless, the NMR data are consistent with the expected transformations. Notably, the 9 ppm downfield shift observed for **6** relative to **2a** mirrors trends previously reported in McKenna-type reactions, supporting the formation of a silylated phosphonate species.^28^ Similarly, the ^31^P­{^1^H} resonance of **7** aligns closely with that of **2a**, further corroborating its assignment. Lastly, an upfield shift of 6.1 ppm was observed for **8** with respect to **2a**, consistent with the trend of dealkylation of dialkyl phosphonates to their corresponding phosphonic acids.^29,30^

**Scheme 3:**
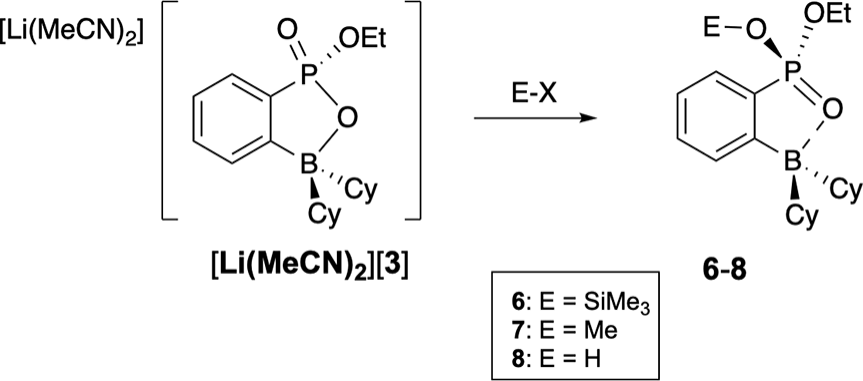
Reactions of [Li­(MeCN)_**2**_]­[**3**] with Electrophiles, Where X = Cl or OTf

Both [Li­(MeCN)_2_]­[**3**] and **2a** exhibited weak fluorescence in the solid state and in solution when exposed to UV light. The UV–vis absorption spectra of [Li­(MeCN)_2_]­[**3**] and **2a** in THF reveal absorption bands with λ_abs_ values of 275 and 282 nm, respectively. The λ_abs_ values were relatively insensitive to the choice of solvent (hexanes, THF, MeCN, MeOH, CHCl_3_, and AcOH; Figures S44 and S51).

Preliminary studies of the fluorescence spectra, however, indicated significant solvent-dependent emissions for both compounds. Both **2a** and [Li­(MeCN)_2_]­[**3**] are weakly emissive, with quantum yields (Φ_F_) less than ca. 0.01, and as the nature of the solvent is changed, the relative ratios of higher energy (ca. 307–319 nm) and lower energy (350–401 nm) bands change. While further detailed studies are required to rigorously make assignments, we note that this type of dual emission has been previously well documented in systems with much greater quantum yields and attributed to concurrent emission from tetracoordinated and trisubstituted borane species.^31,32^

Good solubility in organic solvents is a desired attribute of lithium salts for consideration as part of **LIB** electrolyte solutions. [Li­(MeCN)_2_]­[**3**] was thus surveyed for these properties. [Li­(MeCN)_2_]­[**3**] is soluble in a range of polar solvents such as H_2_O (0.2 M), ethanol (0.4 M), THF (0.2 M), DMSO (0.5 M), diethyl carbonate (DEC) (0.3 M), and ethyl methyl carbonate (EMC) (0.2 M). This material also displayed limited solubility in less-polar solvents such as benzene, toluene, MeCN, and CHCl_3_ and was insoluble in hexanes and diethyl ether. [Li­(MeCN)_2_]­[**3**] is soluble to at least 0.7 M in a mixture of DEC/EMC alkyl carbonate solutions. As [Li­(EtOAc)_3_]­[**4**] was isolated as an oil, it might be expected to exhibit greater solubility. This is indeed true, and solutions of 0.8–1.3 M are possible in both nonpolar (hexanes, toluene) and polar (DCM, DEC, EMC) solvents. In addition, the possible development of salts such as [Li­(EtOAc)_3_]­[**4**] as ionic liquids is being examined.^33–35^

Thermal stability is another desired attribute of lithium salts for consideration as part of **LIB** electrolyte solutions. Thermal gravimetric analysis (TGA, Figure S56) of [Li­(MeCN)_2_]­[**3**] reveals an initial slow weight loss from 82 to 127 °C, consistent with release of two MeCN, which is followed by more rapid weight losses (decomposition) above 200 °C. In DMSO solution, [Li­(MeCN)_2_]­[**3**] decomposes at 160 °C over the course of hours, slowly producing a number of decomposition species, from which lithium ethyl (phenyl) phosphonate (δ = 7.5 ppm) could be identified.

In summary, we have introduced *ortho*-phenyl-phosphonate-boranes, 1-BR_2_-2-{P­(O)­(OEt)_2_}­C_6_H_4_, as synthetically accessible precursors for a new class of lithium salts, [Li­(S)_*n*_]­[1-BR_2_-2-{P­(O)_2_(OEt)}­C_6_H_4_], and established their structural, thermal, and solubility profiles. Other key findings include (i) a synthetic pathway allowing steric tuning at boron; (ii) intramolecular P=O···B interactions that persist in the neutral and anionic states; (iii) high thermal robustness and carbonate solubility for potential phosphorus-rich **WCA**; and (iv) reactivity toward electrophiles, providing a handle for further functionalization.

## Supplementary Material



## Notes

Experimental procedures and characterization data for all reactions and products, including ^1^H, ^13^C­{^1^H}, amd ^31^P­{^1^H} NMR spectra amd crystallographic Data for **2a** and [Li­(MeCN)_2_]­[**3**] (PDF)

## †

Deceased, March 3, 2024.
