# Supplementary material for: Dealkylation as a Strategy to Synthesize Unconventional Lithium Salts from ortho-Phenyl-phosphonate-boranes
Source: Inorg Chem. 2026 Jan 13;65(3):1738–42. doi: 10.1021/acs.inorgchem.5c05101 (PMC12848966; doi:10.1021/acs.inorgchem.5c05101)
Supplement: Supplementary file 1 [file ic5c05101_si_001.pdf]

Electronic Supporting Information

For

Dealkylation as a Strategy to Unconventional Lithium Salts from ortho-Phenyl-phosphonate-boranes

Anthony D. Kornokovich[a], Arnold L. Rheingold[b], Vallabha R. Rikka[c], Wan Si Tang[c], Judith A. Jeevarajan[c], and John D. Protasiewicz[a*]

[a]Department of Chemistry, Case Western Reserve University, 2080 Adelbert Road, Cleveland, Ohio 44106, United States

[b]Department of Chemistry and Biochemistry , University of California, San Diego, La Jolla, California 92093, United States

[c]Electrochemical Safety Research Institute, UL Research Institutes, 5000 Gulf Freeway, Houston, TX 77204, United States

E-mail: protasiewicz@case.edu

1. Experimental **S2**
2. NMR Spectra of compounds **1-8** **S9**
3. Solubility of [Li(MeCN)$_2$][**3**] and [Li(EtOAc)$_3$][**4**] **S52**
4. UV/vis absorption and emission spectra of **2a** and [Li(MeCN)$_2$][**3**] **S54**
5. Thermal gravimetric analysis of [Li(MeCN)$_2$][**3**] **S60**
6. Thermolysis of [Li(MeCN)$_2$][**3**] in solution **S61**
7. Crystal data for compounds **2a** and [Li(MeCN)$_2$][**3**] **S62**
8. DFT calculations **S78**
9. References **S82**

1. Experimental

General

All reactions were conducted under a nitrogen atmosphere using standard Schlenk line techniques, unless otherwise stated. All glassware was dried overnight in an oven. Diethyl ether, dichloromethane, and toluene were purified using a solvent purification system MB-SPS/ 16-229. Other solvents and reagents were used without further purification unless stated otherwise. Diethylphosphite, triphenylphosphine, ^{i}PrLi, ^{n}BuLi, lithium iodide, sodium iodide, potassium iodide, 1-bromo-2-iodobenzene, (2-bromophenyl)boronic acid, copper (I) oxide, phenanthroline, diisopropylethylamine, dicyclohexylchloroborane, chloro (dimethylsulfide) borane complex, 1-hexene, (+)-chlorodiisopinocamphylborane, dicyclohexylchloroborane, chloro(trimethyl)silane, methyl triflate, and palladium (II) acetate were all purchased commercially from Millipore Sigma, Fisher Scientific, or TCI America Research Chemicals. Diethyl (2-bromophenyl) phosphonate and dihexylchloroborane were prepared using modified literature procedures.[1-3] ^{1}H, ^{13}C{^{1}H}, ^{31}P{^{1}H} NMR spectra were recorded with a 500 MHz Brüker BioSpin NMR spectrometer tuned to 500, 202, and 126 MHz respectively. Melting Point data was collected using a capillary Melt-temp apparatus. Elemental analysis was done by Robertson Microlit Laboratories, Madison, New Jersey. TGA experiments were performed on TGA 550 using a platinum pan (100 *μL)* with an N_2 rate of 60 mL min^{-1}. The oven temperature was heated from room temperature up to 700 °C with a continuous ramp of 20 °C min^{-1}. UV/Vis and fluorescence data were recorded by using a Cary 50 Bio UV/Vis spectrophotometer and a Cary Eclipse spectrometer, respectively. L-Tryptophan in distilled water was used as the quantum yield standard. Safety statement: No uncommon hazards are noted.

Diethyl (2-bromophenyl) phosphonate (1)[2]

In a 500 mL round-bottom flask, copper (I) oxide (0.442 g, 3.00 mmol), phenanthroline (1.11 g, 6.00 mmol), and (2-bromophenyl) boronic acid (24.1 g, 120 mmol) were sequentially added. Diethyl phosphite (7.73 mL, 60 mmol), diisopropylethylamine (31.4 mL, 180 mmol), and acetonitrile (200 mL) were added sequentially via syringe. The solution appeared red. The reaction was stirred open to the atmosphere for 22 hours. The solution transformed from red to dark brown. The reaction mixture was passed through a plug of Celite, and the filtrate was collected. The volatiles were removed via rotor evaporation, leaving a brown oil (21.5 g). The oil was purified via column chromatography using ethyl acetate and hexanes (25:75) as the eluent. The volatiles were removed via rotor evaporation, leaving an orange oil (8.59 g, 48.8%). ^{1}H NMR (500 MHz, Chloroform-d) δ (ppm) 8.05-8.00 (m, 1H), 7.68 (t, *J* = 5 Hz, 1H), 7.43-7.36 (m, 2H), 4.22-4.14 (m, 4H), 1.37 (t, *J* = 7.5 Hz, 6H). ^{31}P{^{1}H} NMR (202 MHz, Chloroform-d) δ (ppm) 14.8 (s).

Synthesis of 1-BCy$_2$-2-{P(O)(OEt)$_2$}C$_6$H$_4$ (2a)

Into a flame-dried 500 mL round-bottom flask under N_2 was added **1** (4.00 g, 13.7 mmol) and anhydrous toluene (150 mL). The solution cooled to -78°C. A solution of ^{i}PrLi (23.40 mL, 16.38 mmol, 0.7 M) in pentane was added dropwise via syringe into the reaction flask. Upon addition, the solution transforms from a pale yellow to a dark orange. The reaction mixture was stirred for two hours at -78°C. A solution of Cy$_2$BCl (16.4 mL, 16.4 mmol, 1.0 M) in hexanes was added dropwise via syringe into the reaction flask. The reaction continued stirring for one hour at -78°C and was warmed to room temperature for an additional hour. Volatiles were removed under rotor evaporation to afford a brown residue. The crude product was dissolved in diethyl ether (50 mL), water (30 mL), and 1 M HCl (30 mL), and stirred for 30 minutes. The organic phase was separated, and the aqueous layer was extracted with diethyl ether (3 × 25 mL). The combined organic extracts were dried over $MgSO_4$, filtered, and concentrated under reduced pressure to yield a pale-yellow oil that solidified upon standing. Purification by column chromatography on silica gel using a 1:1 mixture of dichloromethane and hexanes as eluent. The volatiles were removed via rotor evaporation to afford **2a** as a white crystalline solid (3.38 g, 63.5%).^{1}H NMR (500 MHz, Chloroform-d) δ (ppm) 7.50-7.46 (m, 3H), 7.27-7.23 (m, 1H), 4.28-4.14 (m, 4H), 1.70 (d, J = 15Hz, 2H), 1.61 (d, J = 9 Hz, 6H), 1.37 (t, J = 7.5 Hz, 6 H), 1.21-1.03 (m, 6H), 0.95 (q, J = 10 Hz, 2H), 0.79-0.66 (m, 4H). ^{13}C{^{1}H} (126 MHz, Chloroform-d) δ (ppm) 170.9 (bs, ipso C-B),132.5 (s), 130.3 (d, J = 21 Hz), 126.8 (d, J = 16 Hz), 125.5 (d, J = 15 Hz), 122.1 (d, J = 186 Hz)), 65.1 (s), 32.2 (s), 30.5 (d, J = 34 Hz), 29.3 (d, J = 28 Hz), 28.2 (s), 16.6 (s).^{31}P{^{1}H} NMR (202 MHz, Chloroform-d) δ (ppm) 42.7 (s). M.P. 55-61°C.

Synthesis of [Li(MeCN)$_2$] [1-BCy$_2$-2-{P(O)$_2$(OEt)}C$_6$H$_4$] ([Li(MeCN)$_2$][3])

A dry 250 mL round-bottom flask was charged with anhydrous MeCN (75 mL) and **2a** (2.75 g, 7.05 mmol) under N_2. LiI (1.17 g, 8.46 mmol) was added, and the reaction was heated under reflux for 26 hours. After cooling to room temperature, a white precipitate was evident. The precipitate was collected via vacuum filtration, washed with acetonitrile (75 mL), and dried under reduced pressure (0.06 mmHg), leaving a free-flowing white solid (1.88 g, 59.3%).^{1}H NMR (500 MHz, DMSO-d$_6$) δ (ppm) 7.22 (t, J = 7.5 Hz, 1H), 7.15-7.11 (m, 2H), 6.98-6.94 (m, 1H) 3.86 (p, J = 7.5 Hz, 2H), 2.07 (s, 6H),1.63 (t, J = 10 Hz, 2H), 1.57-1.49 (m, 6H), 1.26 (d, J = 10 Hz, 1H), 1.19 (d, J = 10 Hz, 1H), 1.13 (t, J = 7.5 Hz, 3H) 1.04-0.87 (m, 8H), 0.66 (q, J = 10 Hz, 1H) 0.47 (q, J = 10 Hz, 1H)), 0.38 (q, J = 10 Hz, 2H). ^{13}C{^{1}H} NMR (126 MHz, DMSO-d$_6$) δ (ppm) 167.3 (d, J = 35 Hz), 133.6 (d, J = 176 Hz),128.2 (d, J = 19 Hz), 127.7 (s), 125.0 (d, J = 16 Hz), 122.6 (d, J = 14 Hz), 117.7 (s), 58.6 (s), 31.5 (s), 29.6 (s), 29.3 (s), 28.7-28.4 (m), 27.3 (s, J = 10 Hz), 16.5 (s), 0.7 (s). ^{31}P{^{1}H} NMR (202 MHz, DMSO-d$_6$) δ (ppm) 32.9 (s). Anal. Calc. for LiC$_{24}$H$_{37}$BN$_2$O$_3$P, Calculated: C, 63.96; H, 8.22; N, 6.21; Found: C, 63.68; H, 8.42; N, 5.99. M.P 123-126°C.

[Li(MeCN)][1-BCy$_2$-2-{P(O)$_2$(OEt)}C$_6$H$_4$] ([Li(MeCN)][3])

Into a dry 20 mL vial charged with [Li(MeCN)$_2$][**3**] (0.650g). The solid was further dried under reduced pressure (0.06 mmHg) while heating at 80°C for two hours to afford [Li(MeCN)][**3**] as a free-flowing solid (0.554 g, 93.8%). The ^{1}H and ^{31}P{^{1}H} NMR spectrum are essentially the same as **[Li(MeCN)$_2$][3]** indicating MeCN is replaced with DMSO to afford [Li(DMSO)$_n$][3]. ^{1}H NMR (500 MHz, DMSO-d$_6$) δ (ppm) 7.22 (t, J = 7.5 Hz, 1H), 7.15-7.11 (m, 2H), 6.98-6.94 (m, 1H) 3.86 (p, J = 7.5 Hz, 2H), 2.07 (s, 3H), 1.63 (t, J = 10 Hz, 2H), 1.57-1.49 (m, 6H), 1.26 (d, J = 10 Hz, 1H), 1.19 (d, J = 10 Hz, 1H), 1.13 (t, J = 7.5 Hz, 3H) 1.04-0.87 (m, 8H), 0.66 (q, J = 10 Hz, 1H) 0.47 (q, J = 10 Hz, 1H)), 0.38 (q, J = 10 Hz, 2H). ^{31}P{^{1}H} NMR (202 MHz, DMSO-d$_6$) δ (ppm) 32.9 (s).

[Li(DEC)$_2$][1-BCy$_2$-2-{P(O)$_2$(OEt)}C$_6$H$_4$] ([Li(DEC)$_2$][3])

A dry 20 mL vial was charged with [Li(MeCN)$_2$][**3**] (0.453 g) and diethyl carbonate (**DEC**) (4 mL). The solution was filtered through cotton into a dry 20 mL vial. Volatiles were removed under reduced pressure (0.06 mmHg) while heating to 85°C for three hours. A clear oil was obtained (0.582 g, 95.7%). The ^{1}H and ^{31}P{^{1}H} NMR spectrum are essentially the same as **[Li(MeCN)$_2$][3]** indicating DEC is replaced with DMSO to afford [Li(DMSO)$_n$][3]. ^{1}H NMR (500 MHz, DMSO-d$_6$) δ (ppm) 7.22 (t, J = 10 Hz, 1H), 7.15-7.10 (m, 2H), 6.98-6.94 (m, 1H), 4.10 (q, J = 10 Hz, 8H), 3.86 (p, J = 7.5 Hz, 2 H), 1.63 (t, J = 10 Hz, 2H), 1.55-1.50 (m, 6H), 1.25 (d, J = 5 Hz, 1H), 1.20 (t, J = 7.5 Hz, 14H), 1.13 (t, J = 7.5 Hz, 3H), 1.04-0.89 (m, 8H), 0.65 (q, J = 7.5 Hz, 1H) 0.47 (q, J = 7.5 Hz, 1H)), 0.42-0.34 (m, 2H). ^{31}P{^{1}H} NMR (202 MHz, DMSO-d$_6$) δ (ppm) 32.9 (s).

Synthesis of [Na(MeCN)][1-BCy$_2$-2-{P(O)$_2$(OEt)}C$_6$H$_4$] ([Na(MeCN)][3])

A dry 100 mL round-bottom flask was charged with **2a** (1.10 g, 7.05 mmol) and anhydrous MeCN (40 mL) under N$_2$. NaI (0.513 g, 3.38 mmol) was added, and the reaction was heated under reflux for 22 hours. After cooling to room temperature, volatiles were removed via rotor evaporation, yielding a white solid. The solid was further washed with hexanes and dried under reduced pressure (0.06 mmHg), leaving a free-flowing white solid (0.607 g, 50.9%).^{1}H NMR (500 MHz, DMSO-d$_6$) δ (ppm) 7.22-7.19 (m, 1H), 7.14-7.09 (m, 2H), 6.95 (q, J = 6.5 Hz, 1H), 3.85 (p, J = 7.5 Hz, 2H), 2.07 (s, 3H), 1.63 (t, J = 14 Hz, 2H), 1.56-1.49 (bm, 6H), 1.25 (d, J = 12 Hz, 1H), 1.19 (d, J = 12 Hz, 1H), 1.12 (t, J = 7.5 Hz, 3H), 1.04-0.86 (bm, 8H), 0.65 (q, J = 13 Hz, 1H), 0.47 (q, J = 13 Hz, 1H), 0.37 (q, J = 13 Hz, 2H). ^{13}C{^{1}H} NMR (126 MHz, DMSO-d$_6$) δ (ppm) 134.5 (d, J = 179 Hz), 128.6 (d, J = 18.9 Hz), 128.1 (d, J = 2.5 Hz), 125.3 (d, J = 16.4 Hz), 123.0 (d, J = 13.9 Hz), 118.1 (s), 59.0 (d, J = 6 Hz), 32.1 (bs), 30.0 (s), 29.1 (s), 29.0 (s), 28.9 (s), 27.8 (d, J = 10 Hz), 16.9 (d, J = 5 Hz), 1.2 (s). ^{31}P{^{1}H} NMR (202 MHz, DMSO-d$_6$) δ (ppm) 33.0 (s). M.P. 132-135°C.

Synthesis of [K(MeCN)][1-BCy$_2$-2-{P(O)$_2$(OEt)}C$_6$H$_4$] ([K(MeCN)][3])

A dry 100 mL round-bottom flask was charged with **2a** (1.37 g, 3.51 mmol) and anhydrous MeCN (40 mL) under N_2. KI (0.728 g, 4.21 mmol) was added, and the reaction was heated under reflux for 24 hours. After cooling to room temperature, the volatiles were removed via rotor evaporation, leaving a white solid. The solid was purified by redissolving in MeCN (2mL) and layering it in toluene (2mL), which was placed in the freezer (-30°C) for 2 hours. A crystalline solid was collected via vacuum filtration and further dried under reduced pressure (0.06 mmHg) leaving a free flowing solid (0.794 g, 51.3%).^{1}H NMR (500 MHz, DMSO-d$_6$*)* δ (ppm) 7.20 (t, *J* = 9 Hz, 1H), 7.13-7.08 (m, 2H), 6.95 (q, *J* = 6.5 Hz, 1H), 3.84 (p, *J* = 7.5 Hz, 2H), 2.07 (s, 3H), 1.63 (t, *J* = 14 Hz, 2H), 1.55-1.50 (m, 6H), 1.25 (d, *J* = 12 Hz, 1H), 1.17 (d, *J* = 13 Hz, 1H), 1.11 (t, *J* = 7.5 Hz, 3H), 1.04-0.94 (m, 8H), 0.644 (q, *J* = 10 Hz, 1H), 0.46 (q, *J* = 10 Hz, 1H), 0.40-0.32 (m, 2H). ^{31}P{^{1}H} NMR (202 MHz, DMSO-d$_6$) δ (ppm) 32.9 (s). M.P. 115-120°C.

Synthesis of 1-BIpc$_2$-2-{P(O)(OEt)$_2$}C$_6$H$_4$ (2b)

Into a dry 100 mL round-bottom flask charged with N_2 was added **1** (2.00 g, 6.82 mmol) and anhydrous toluene (50 mL) were added. The solution was cooled to -78°C for 30 minutes. A solution of of ^{n}BuLi (2.73 mL, 6.82 mmol, 2.5 M) in hexanes was added dropwise via syringe to the reaction flask. Upon addition of ^{n}BuLi the solution went from pale yellow to dark red. The reaction was stirred for 1 hour at -78°C. In a glove bag (+)-Ipc$_2$BCl (3.55 mL, 5.68 mmol, 1.6 M in hexanes) was measured via syringe and transferred into the reaction flask dropwise via syringe. The solution went from a dark red to a light orange color upon adding (+)-Ipc$_2$BCl. The reaction was stirred for 1 hour at -78°C and was warmed to room temperature for an additional hour. Volatiles were removed via rotor evaporation to afford a brown oil (4.78 g). The brown oil was dissolved in a minimal amount of ethanol and layered with hexane and placed in the freezer (-20°C) for crystallization. A crystalline solid was obtained via vacuum filtration and washed with ~20 mL of ethanol (-20°C). A white solid was obtained (0.719 g, 25.4%).^{1}H NMR (500 MHz, Chloroform-d) δ (ppm) 7.85-7.82 (m, 1H), 7.50-7.44 (m, 2H), 7.29-7.24 (m, 1H), 4.29-4.16 (m, 4H), 2.12-2.03 (m, 5H), 1.89-1.84 (m, 3H), 1.56-1.51 (m, 2H), 1.47-1.41(m, 2H), 1.38-1.32 (m, 6H), 1.28-1.22 (m, 1H), 1.19-1.15 (m, 1H), 1.13-1.09 (m, 12H),0.97 (d, *J* = 10 Hz, 3H), 0,53 (d, *J* = 10 Hz, 3H). ^{13}C{^{1}H} (126 MHz, Chloroform-d) δ (ppm) 131.7 (d, *J* = 20 Hz), 131.1 (d, *J* = 3.8 Hz), 126.5 (d, *J* = 18 Hz), 125.9 (d, *J* = 15 Hz), 123.4 (d, *J* = 176 Hz), 64.9 (dd, *J* = 37, 7.5 Hz), 49.6 (s), 49.1 (s), 42.6 (s), 42.4 (s), 39.8 (s), 39.5 (s), 39.0 (s), 38.7 (s), 32.7 (s), 31.9 (s), 31.8 (s), 30.3 (s), 28.4 (d, *J* = 8.8 Hz), 24.4 (s), 24.1 (s), 23.3 (s), 23.1 (s), 16.3 (dd). ^{31}P{^{1}H} NMR (202 MHz, Chloroform-d) δ (ppm) 40.65 (s). Anal. Calc. for $C_{30}H_{48}BO_3P$, Calculated: C, 72.28; H, 9.71; Found: C, 71.45; H, 9.73. M.P: 121-123°C.

Synthesis of [Li(EtOAC)3][1-BIpc$_2$-2-{P(O)$_2$(OEt)}C$_6$H$_4$] ([Li(EtOAc)$_3$][4])

A dry 100 mL round-bottom flask was charged with anhydrous MeCN (40 mL) and **2b** (0.456 g, 0.915 mmol) under N_2. LiI (0.176 g, 1.30 mmol) was added, and the reaction was heated under reflux for 18 hours. The reaction was cooled to room temperature. The volatiles were removed via rotor evaporation to afford a waxy white solid. Purification was achieved by column chromatography on silica gel using a 1:1 mixture of EtOAc and hexanes as the eluent, followed by a transition to pure ethyl acetate. The volatiles were removed via rotor evaporation to afford [Li(EtOAc)$_3$][**4**] as a clear viscous oil. The oil was triturated with ~10 mL of hexanes twice and dried under high vacuum (0.06 mmHg) for two hours to yield a clear oil (0.32 g, 47 %). ^{1}H NMR (500 MHz, DMSO-d$_6$) δ (ppm) 7.48 (bt, J = 10 Hz, 1H), 7.25 (bt, J = 9 Hz, 1H), 7.12-7.08 (m, 1H), 6.98 (q, J = 6 Hz, 1H), 4.02 (q, J = 7 Hz, 6H), 3.98-3.88 (m, 2H), 2.33-2.23 (m, 1H), 2.12-2.02 (m, 2H), 1.99 (s, 9H), 1.93-1.79 (m, 3H), 1.77-1.69 (m, 4H), 1.62-1.58 (m, 1H), 1.48-1.45 (m, 1H), 1.40-1.30 (m, 3H), 1.17 (t, J = 5 Hz, 13H), 1.12-1.02 (m, 16H), 0.95-0.87 (m, 6H), 0.48 (d, J = 7 Hz, 1H), 0.34 (d, J = 7 Hz, 1H). ^{13}C{^{1}H} (126 MHz, DMSO-d$_6$) δ (ppm) 170.3 (s), 135.8 (d, J = 176 Hz), 131.6 (d, J = 10 Hz), 131.4 (d, J = 10 Hz), 127.2 (s), 125.3 (d, J = 15 Hz), 123.4 (d, J = 14 Hz), 69.4 (s), 59.8 (s), 59.0 (dd, J = 38 and 6 Hz), 49.4 (s), 49.3(d, J = 5 Hz), 49.0 (s), 47.4 (s), 47.1 (s), 42.1 (s), 42.0 (d, J = 4 Hz), 38.2 (s), 37.9 (s), 37.4 (s), 37.2 (s), 33.6 (s), 35.1-31.2 (m), 30.6 (d, J = 5 Hz), 25.0 (s), 24.6 (d, J = 2.5 Hz), 24.3 (s), 23.1-22.9 (m), 20.8 (s), 20.7 (s), 16.8 (d, J = 6 Hz), 14.1 (s). ^{31}P{^{1}H} NMR (202 MHz, DMSO-d$_6$) δ (ppm) 31.9 (s), 31.6 (s). ^{1}H NMR (500 MHz, CDCl$_3$-d) δ (ppm) 7.75 (bs, 1H), 7.36-7.28 (m, 2H), 7.10 (q, J = 6.6 Hz, 1H), 4.05 (q, J = 7.2 Hz, 6H), 3.92-3.84 (bm, 2H), 3.36 (bs, 1H), 2.05-1.97 (bm, overlapping protons, 15H), 1.92-1.82 (bm, overlapping protons, 4H), 1.68-1.20 (bm, overlapping protons, 15H), 1.18- 1.08 (bm, 18H), 1.01-0.92 (bm, 4H), 0.89 (s, 1H), 0.63 (bs, 2H), 0.42 (bs, 1H), 0.22 (bs, 1H). ^{31}P{^{1}H} NMR (202 MHz, CDCl$_3$-d) δ (ppm) 37.7 (bs). ^{13}C{^{1}H} NMR (126 MHz, CDCl$_3$-d) δ (ppm) 172.6 (s), 133.1 (s), 132.9 (s), 129.7 (bs), 125.4 (d, J = 15 Hz), 124.7 (d, J = 15 Hz), 72.4 (s), 62.0 (bm), 61.2 (s), 49.9 (bs), 49.5 (s), 47.9 (s), 47.7 (s), 42.8 (s), 42.5 (s), 41.8 (s), 39.9 (s), 39.6 (s), 38.9 (s), 38.7-38.5 (bm), 38.1 (s), 34.6 (s), 32.6 (bs), 31.9 (s), 30.8 (bs), 28.5 (d, J = 5.0 Hz), 27.8 (s), 24.5 (bs), 24.2 (s), 23.8 (s), 23.2 (d, J = 4.8 Hz), 23.1 (s), 21.3 (s), 20.8 (s), 16.5 (d, J = 6.6 Hz), 14.9 (s).

Synthesis of Dihexylchloroborane[1, 3]

A dry, two-neck 50 mL round-bottom flask was equipped with a short-path distillation apparatus connected to a second, two-neck 50 mL round-bottom flask. The setup was dried under vacuum and purged with N_2 three times over a 15-minute period. Anhydrous DCM (10 mL) was added via syringe, followed by the addition of 1-hexene (10.8 mL, 86.4 mmol) to the reaction flask. The solution was cooled to 0°C for 30 minutes. A bleach bubbler was attached to the second round-bottom flask to scrub odorous vapors from the reaction. Chloro(dimethylsulfide)borane complex (3.00 mL, 28.8 mmol) was added dropwise via syringe, taking care to avoid aggressive bubbling. The reaction mixture was allowed to warm to room temperature and stirred for 1 hour. Volatiles were removed under reduced pressure, leaving a clear oil. The product was purified by vacuum distillation, with the distillate collected at (36-59°C). A clear oil was obtained (5.28 g, 84.6%). ^{1}H NMR (500 MHz, CDCl$_3$-d) δ (ppm) 5.29 (s, 1H), 1.54-1.49 (m, 4H), 1.36-1.28 (m, 16H), 0.89 (t, J = 10 Hz, 6H). ^{13}C{^{1}H} (126 MHz, CDCl$_3$-d) δ (ppm) 53.5 (s), 32.1 (s), 31.9 (s), 29.4 (bs), 24.7 (s), 22.8 (s), 14.2 (s).

Synthesis of 1-BHx$_2$-2-{P(O)(OEt)$_2$}C$_6$H$_4$ (2c)

Into a flame dried 250 mL round bottom flask under N_2 was added **1** (2.00 g, 6.82 mmol) and anhydrous toluene (125 mL). The solution was cooled to -78°C. A solution of ^{i}PrLi (11.7 mL, 8.18 mmol, 0.7M) in pentane was added to the reaction dropwise via syringe to the reaction flask. The color of the solution changed from a pale yellow to a dark orange as it was stirred for two hours. Hx$_2$BCl (1.95 g, 9.00 mmol) was then added dropwise via syringe into the reaction flask. The reaction stirred for one hour at -78°C. The mixture was warmed to room temperature, and the volatiles were removed via rotot evaporation, leaving a brown oil. The oil was dissolved in diethyl ether (50 mL) and water (30 mL). The organic phase was separated, and the aqueous layer was extracted with diethyl ether (3 × 25 mL). The combined organic layers were collected and dried over $MgSO_4$, filtered, and volatiles were removed via rotor evaporation to afford a pale-yellow oil. Purification was performed by column chromatography using a 1:1 mixture of DCM and hexane as the eluent. The volatiles were removed via rotor evaporation to afford **2c** as a clear oil (1.82 g, 67.7%). ^{1}H NMR (500 MHz, Chloroform-d) δ (ppm) 7.51-7.45 (m, 3H), 7.28-7.24 (m, 1H), 4.21-4.03 (m, 4H), 1.34 (t, J = 10 Hz, 6H), 1.23 (bs, 14H), 1.10-1.06 (m, 2H), 0.85 (t, J = 15 Hz, 6H), 0.56-0.54 (m, 4H). ^{13}C{^{1}H} (126 MHz, Chloroform-d) δ (ppm) 132.9 (d, J = 3.8 Hz), 129.5 (d, J = 20.2 Hz), 127.0 (d, J = 16.4 Hz), 125.8 (d, J = 15 Hz), 120.7 (d, J = 189 Hz), 65.1 (d, J = 5 Hz), 33.8 (s), 32.3 (s), 26.6 (s), 25.6 (bs), 23.0 (s), 16.2 (d, J = 6.3 Hz), 14.4 (s). ^{31}P{^{1}H} NMR (202 MHz, Chloroform-d) δ (ppm) 44.1 (s).

Synthesis of [Li(MeCN)][1-BHx$_2$-2-{P(O)$_2$(OEt)}C$_6$H$_4$] ([Li(MeCN)][5])

A dry 100 mL round-bottom flask was charged with **2c** (1.00 g, 2.45 mmol) and anhydrous MeCN (40 mL) under N_2. LiI (0.423 g, 2.94 mmol) was added, and the reaction was heated under reflux for 16 hours. The reaction was cooled to room temperature, and the volatiles were removed via reduced pressure (0.06 mmHg), leaving a yellow oil (0.781g, 77.2%). ^{1}H NMR (500 MHz, DMSO-d$_6$) δ (ppm) 7.25 (t, J = 10 Hz, 1H), 7.15-7.13 (m, 2H), 7.02-6.98 (m, 1H), 3.70-3.53 (m, 2H), 2.07 (s, 3H), 1.22-1.10 (m, 16H), 1.05 (t, J = 7.5 Hz, 3H), 0.81 (q, J = 7.5 Hz, 6H), 0.23 (t, J = 7.5Hz, 4H).^{31}P{^{1}H} NMR (202 MHz, DMSO-d$_6$) δ (ppm) 31.9 (s).

Synthesis of 1-BCy$_2$-2-{P(O)(OTMS)(OEt)}C$_6$H$_4$ (6)

Into a flame-dried 50 mL round-bottom flask charged with N_2 was added [Li(MeCN)$_2$][**3**] (0.257 g, 0.571 mmol) and anhydrous diethyl ether (10 mL). The solution was stirred for 5 minutes to dissolve the solid. Trimethylchlorosilane (0.07 mL, 0.6 mmol) was added dropwise via syringe to the reaction flask. A white precipitate was observed after 1 minute of stirring. The reaction went for 30 minutes and was then transferred to a 25 mL dry Schlenk flask. Volatiles were removed under reduced pressure (0.06 mmHg), leaving a yellow oil (0.080 g, 33%). ^{1}H NMR (500 MHz, DMSO-d$_6$) δ (ppm) 7.60-7.56 (m, 1H), 7.46 (t, J = 6.9 Hz, 1H), 7.32 (d, J = 7.0 Hz,1H), 7.26 (q, J = 7.3 Hz, 1H), 4.14-4.04 (m, 2H), 3.73 (q, J = 6.9 Hz, 6H), 1.63-1.53 (m, 8H), 1.31-1.21 (m, 4H), 1.08 (t, J = 7 Hz, 12H), 1.04-0.83 (m, 8H), 0.68 (q, J = 12.5 Hz, 2H), 0.61-0.52 (m, 4H), 0.36 (s, 15H). ^{31}P{^{1}H} NMR (202 MHz, DMSO-d$_6$) δ (ppm) 33.5 (s). ^{13}C{^{1}H} (126 MHz, DMSO-d$_6$) δ (ppm) 168.4 (d, J = 37 Hz), 131.0 (s), 128.8 (d, J = 25 Hz), 126.3 (d, J = 18 Hz), 124.7 (d, J = 15 Hz), 64.8 (s), 62.9 (s), 31.0 (bs), 29.6 (s), 29.3 (d, J = 10.0 Hz), 28.6-28.3 (m), 27.3 (d, J = 8.8 Hz), 16.0 (d, J = 6.3 Hz), 15.0 (s), 1.8 (s), 0.9 (s), 0.5 (s).

Synthesis of 1-BCy_2-2-{P(O)(OMe)(OEt)}C_6H_4 (7)

Into a dry 50 mL round-bottom flask charged with N_2 was added [Li(MeCN)$_2$][**3**] (0.252 g, 0.560 mmol), and anhydrous THF (5 mL). The solution stirred until the solid completely dissolved. Inside a glove bag, methyl triflate (0.06 mL, 0.6 mmol) was measured via syringe and transferred to the reaction flask dropwise. The solution was stirred for one hour at room temperature. The volatiles were removed via rotor evaporation to afford an opaque oil (0.66 g). The oil was dissolved in hexanes, and a white precipitate was observed. The precipitate was removed via gravity filtration, and the volatiles were removed by rotor evaporation to afford a milky oil (0.18 g). The oil was then passed through silica gel using diethyl ether and hexanes (30:70) as the eluent. The volatiles were removed via reduced pressure (0.06 mmHg) to afford a milky oil (0.13 g). ^{1}H NMR (500 MHz, $CDCl_3$-d) δ (ppm) 7.49-7.43 (m, 3H), 7.26-7.22 (m, 1H), 4.26-4.10 (m, 4H), 3.32 (d, J = 7.9 Hz, 3H),1.79 (p, J = 7.6 Hz, 2H), 1.72-1.59 (m, 12H), 1.41-1.35 (m, 4H), 1.20-1.03 (m, 6H), 0.95 (q, J = 12.3 Hz, 2H), 0.79-0.66 (m, 4H). ^{31}P{^{1}H} NMR (202 MHz, $CDCl_3$-d) δ (ppm) 42.7 (s).

Synthesis of 1-BCy_2-2-{P(O)(OH)(OEt)}C_6H_4 (8)

Into a dry 100 mL round-bottom flask charged with N_2 was added [Li(MeCN)$_2$][**3**] (0.255 g, 0.571 mmol) and anhydrous diethyl ether (25 mL). The solution stirred until the solid was completely dissolved. A solution of 2M HCl (0.34 mL, 0.69 mmol) in diethyl ether was added via syringe to the reaction flask. The solution appeared cloudy after the addition of hydrochloric acid (HCl). The reaction went for 20 minutes at room temperature. The volatiles were removed under reduced pressure (0.06 mmHg) to afford a clear oil (0.101 g, 48.8%). ^{1}H NMR (500 MHz, DMSO-d_6) δ (ppm) 7.36 (t, J = 8.6 Hz, 1H), 7.26 (t, J = 7.8 Hz,2H), 7.21 (d, J = 7.6 Hz, 1H), 7.08 (q, J = 6.1 Hz, 1H), 4.00-3.94 (m, 2H), 3.38 (q, J = 6.8 Hz, 3H), 1.64-1.53 (m, 8H), 1.26-1.17 (m, 8H), 1.09 (t, J = 7.5 Hz, 5H), 1.04-0.90 (m, 8H), 0.68 (q, J = 12.4 Hz, 1H), 0.52-0.41 (m, 3H).^{31}P{^{1}H} NMR (202 MHz, DMSO-d_6) δ (ppm) 36.6 (s).

2. NMR Spectra of compounds 1-8.

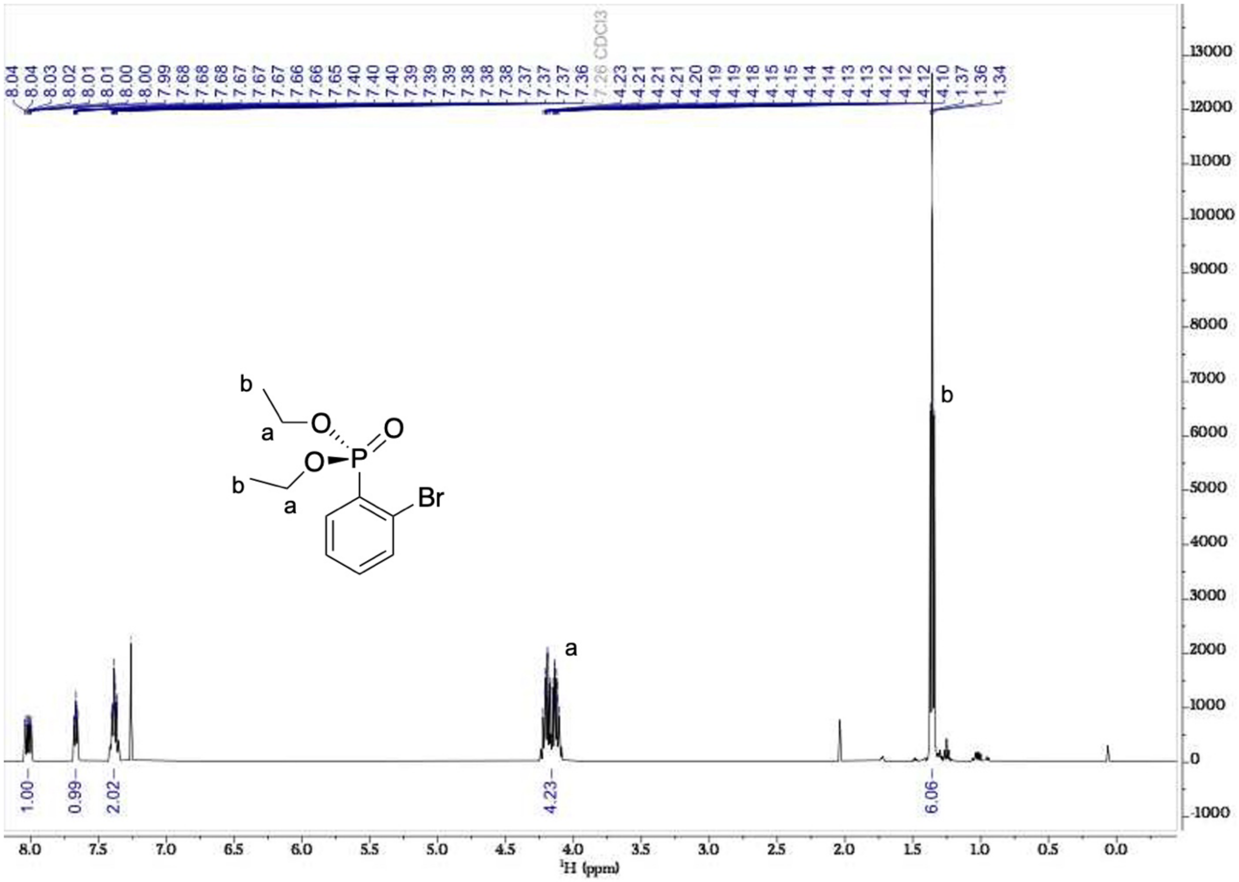

Figure S1. ^{1}H NMR (500 MHz, Chloroform-d) spectrum of **1**.

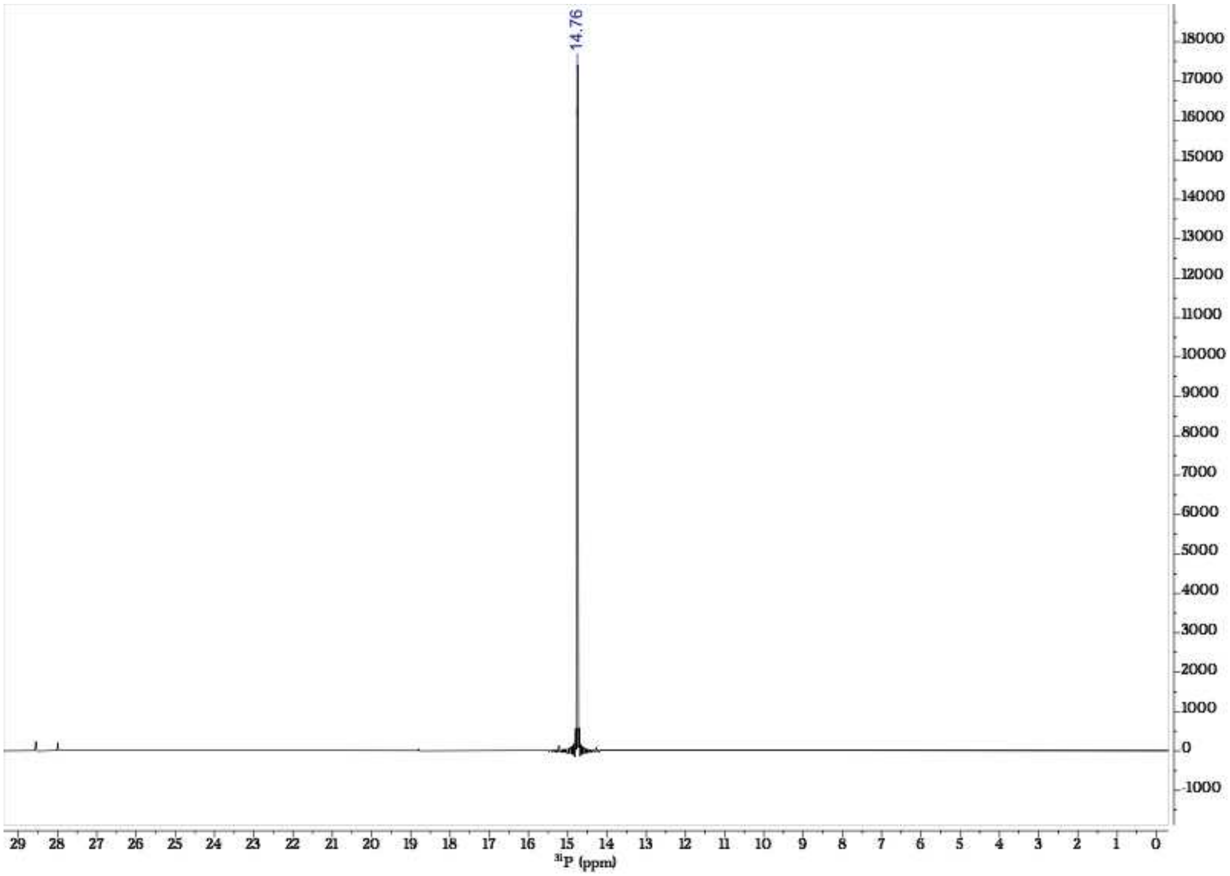

Figure S2. $^{31}P\{^1H\}$ NMR (202 MHz, Chloroform-d) spectrum of **1**.

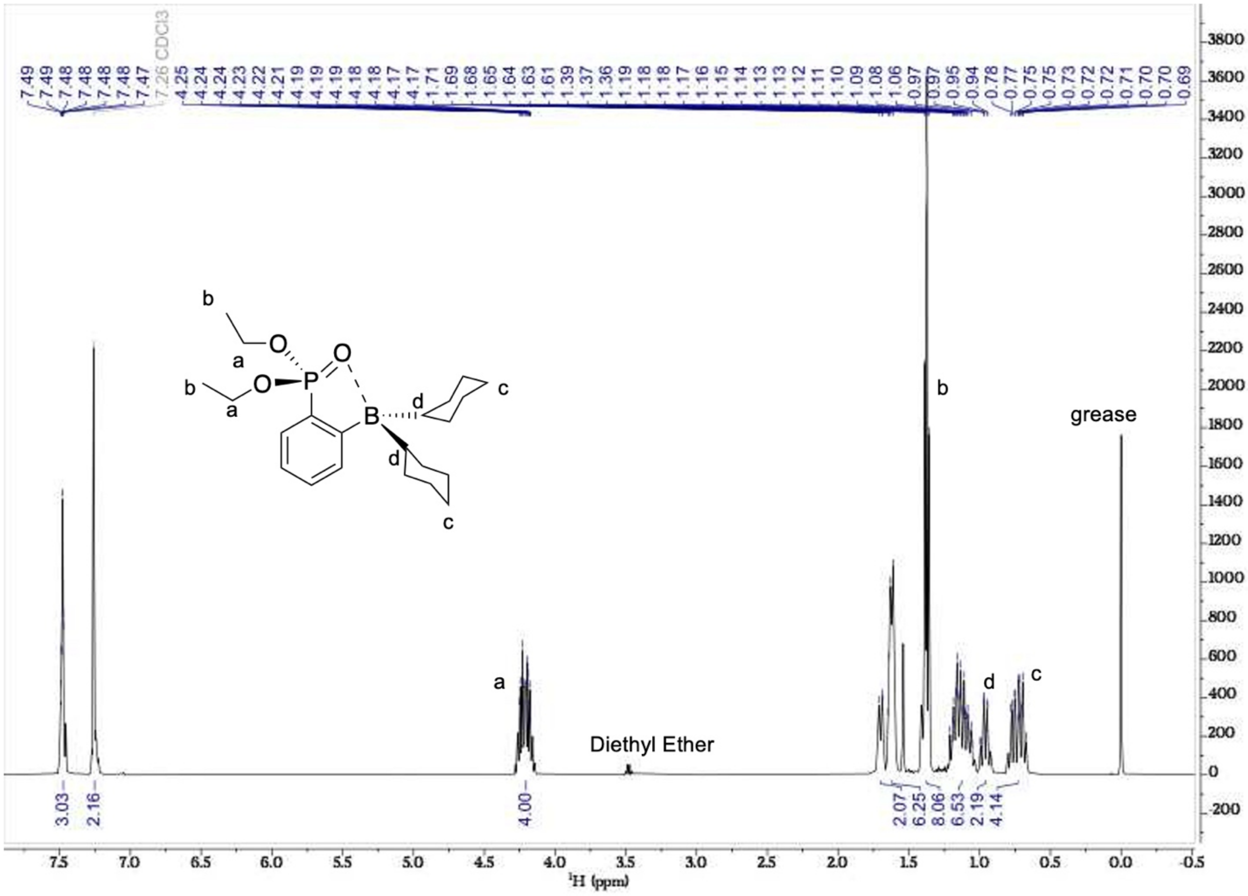

Figure S3. ^{1}H NMR (500 MHz, Chloroform-d) spectrum of **2a**.

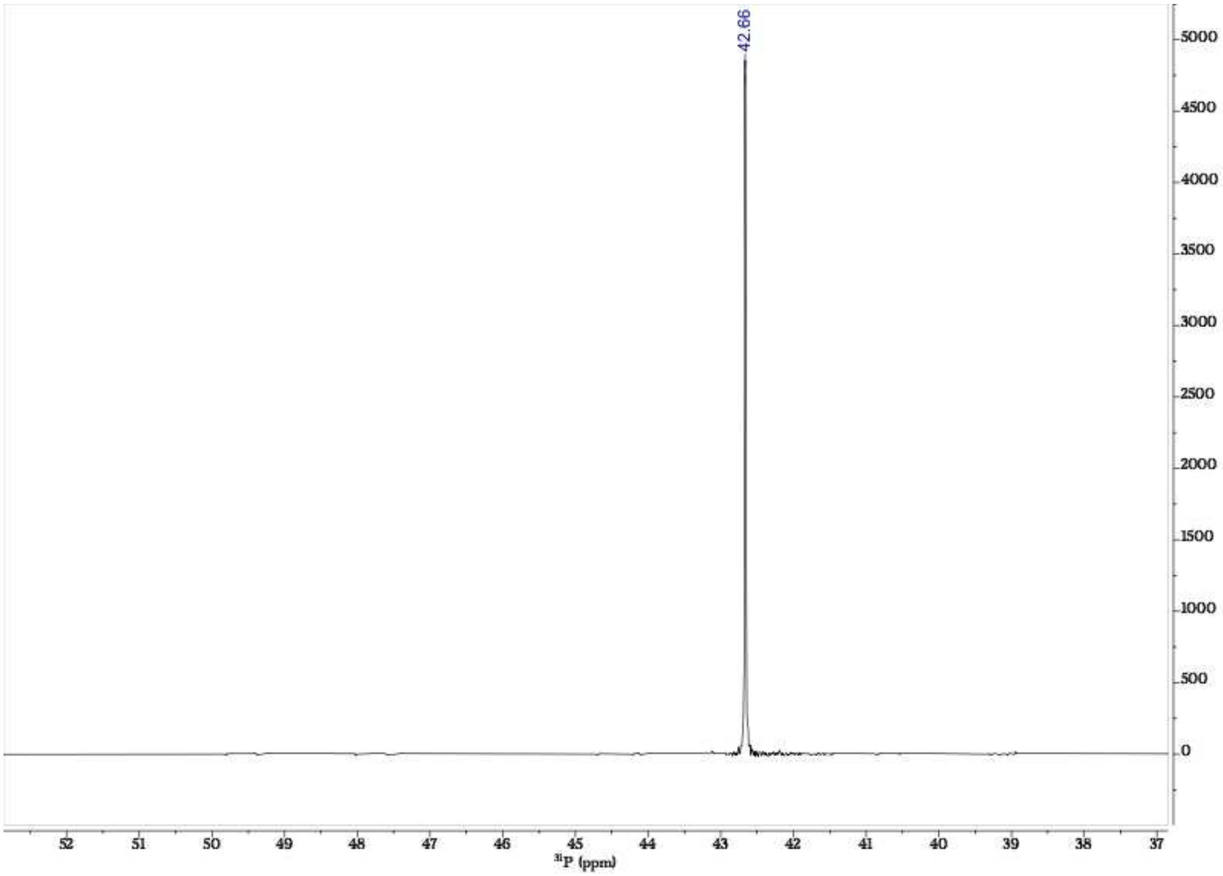

Figure S4. $^{31}P\{^{1}H\}$ NMR (202 MHz, Chloroform-d) spectrum of **2a**.

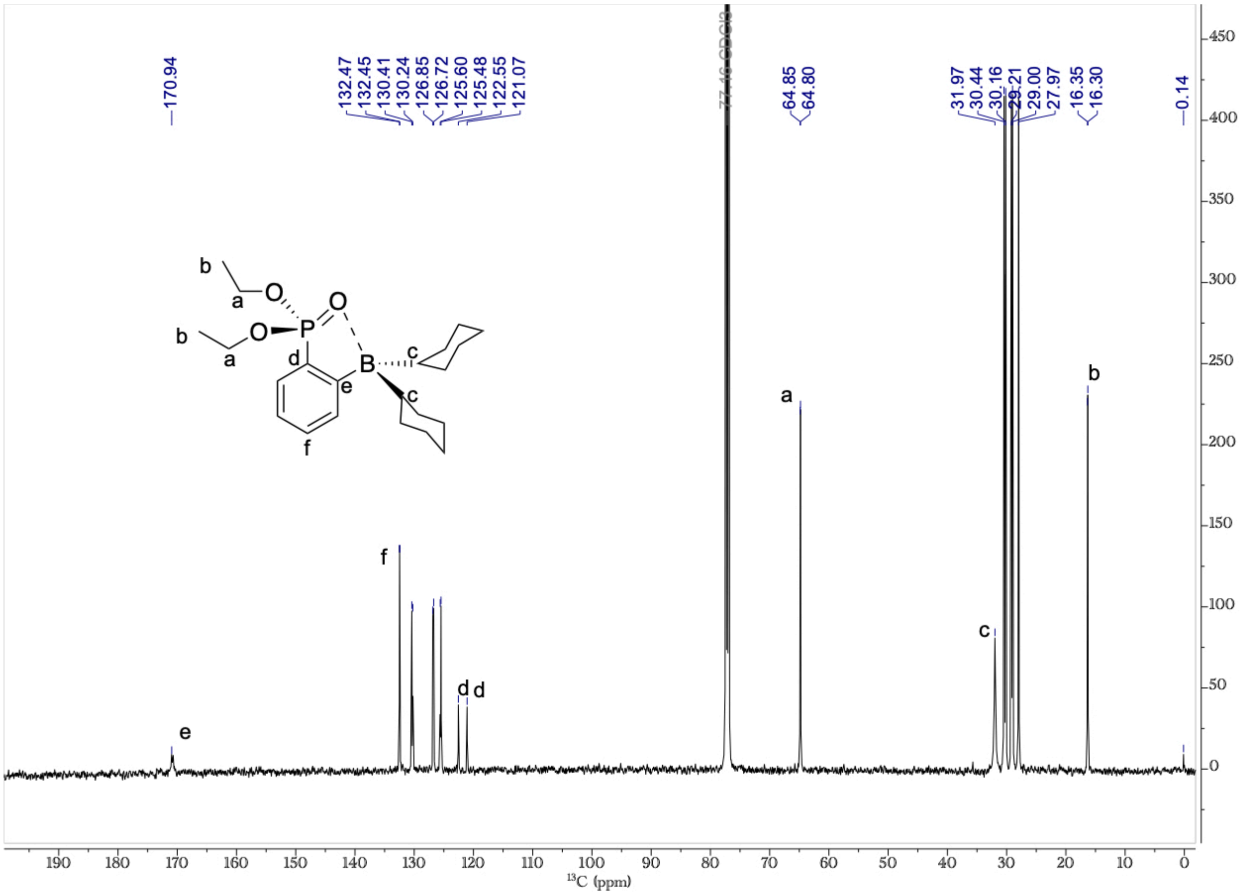

Figure S5. $^{13}C\{^{1}H\}$ NMR (126 MHz, Chloroform-d) spectrum of **2a**.

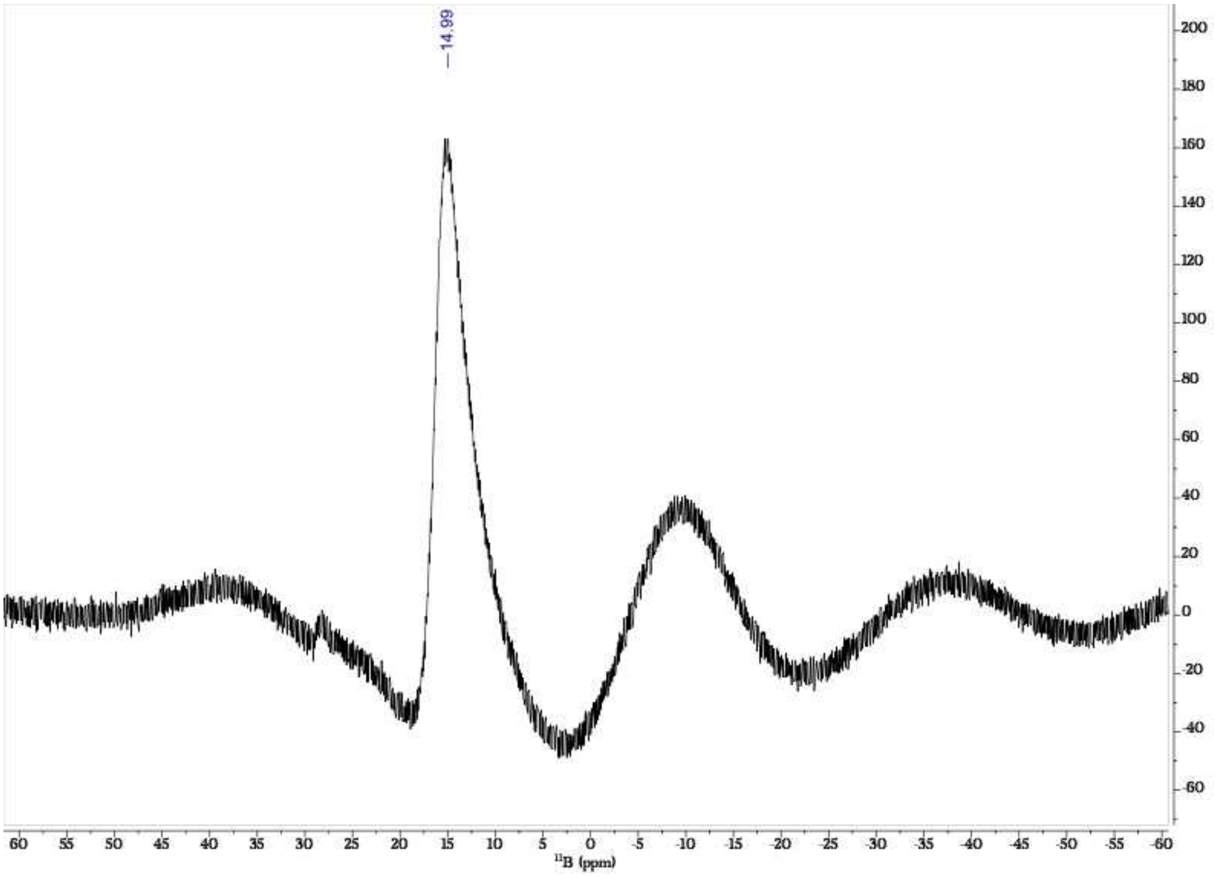

Figure S6. ^{11}B NMR (160 MHz, Chloroform-d) spectrum **2a**.

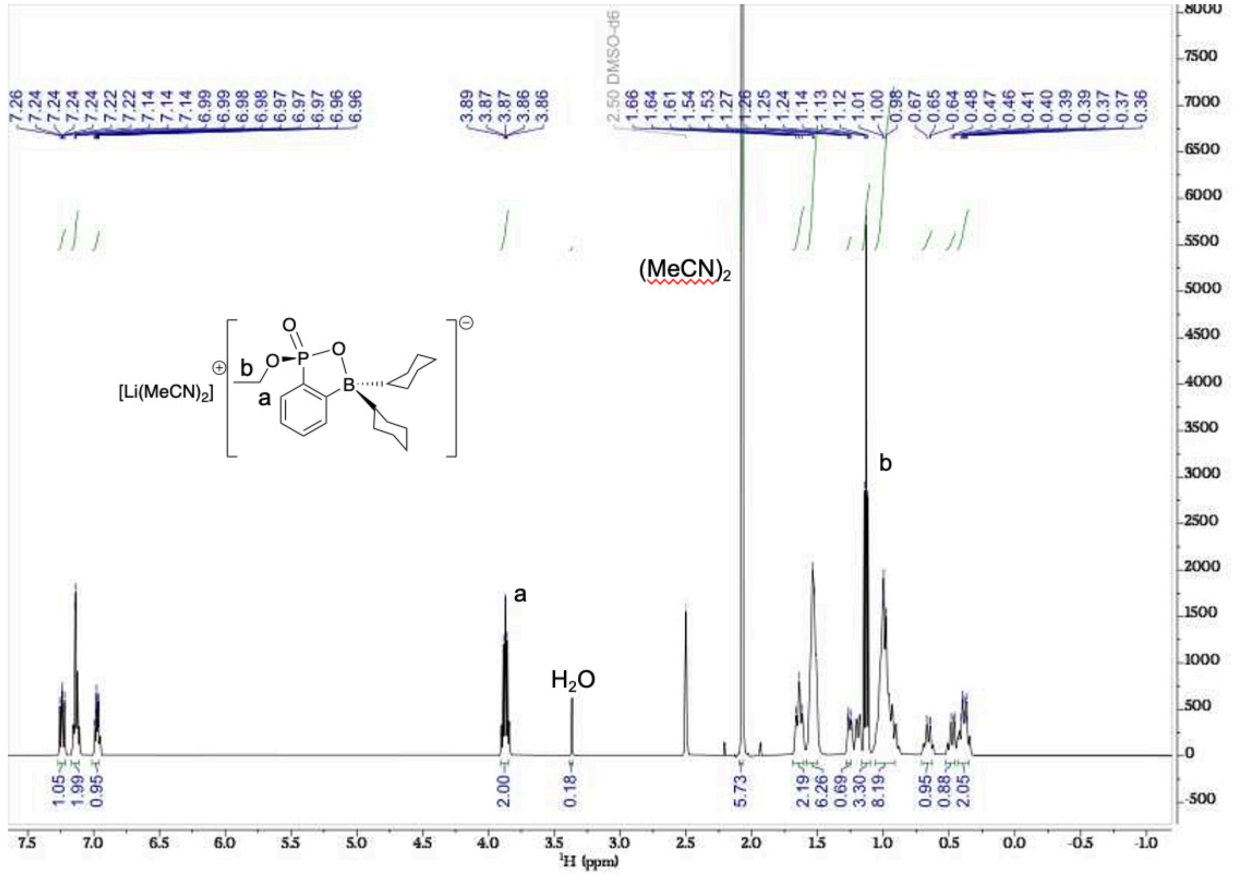

Figure S7. ^{1}H NMR (500 MHz, DMSO-d$_6$) spectrum of [Li(MeCN)$_2$][**3**].

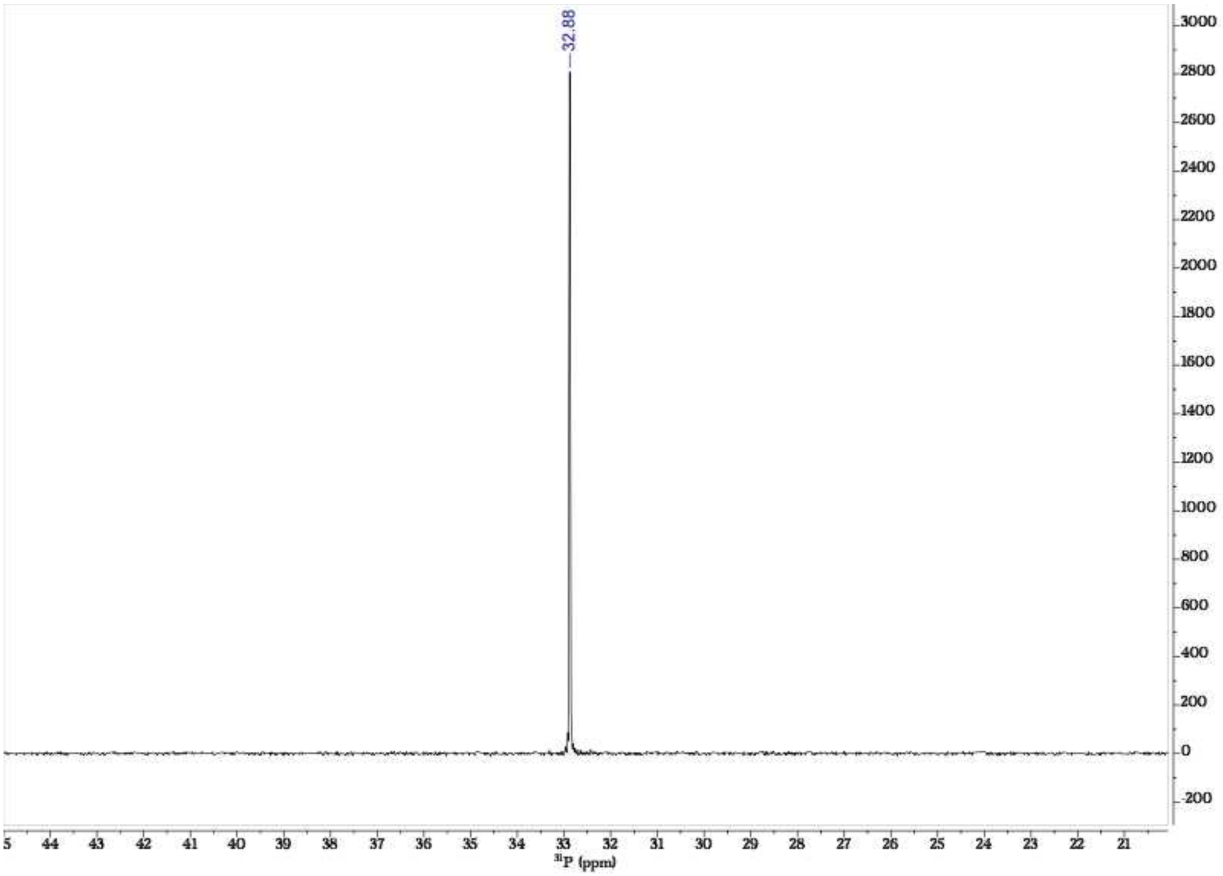

Figure S8. $^{31}P\{^{1}H\}$ NMR (202 MHz, DMSO-D$_6$) spectrum of [Li(MeCN)$_2$][**3**].

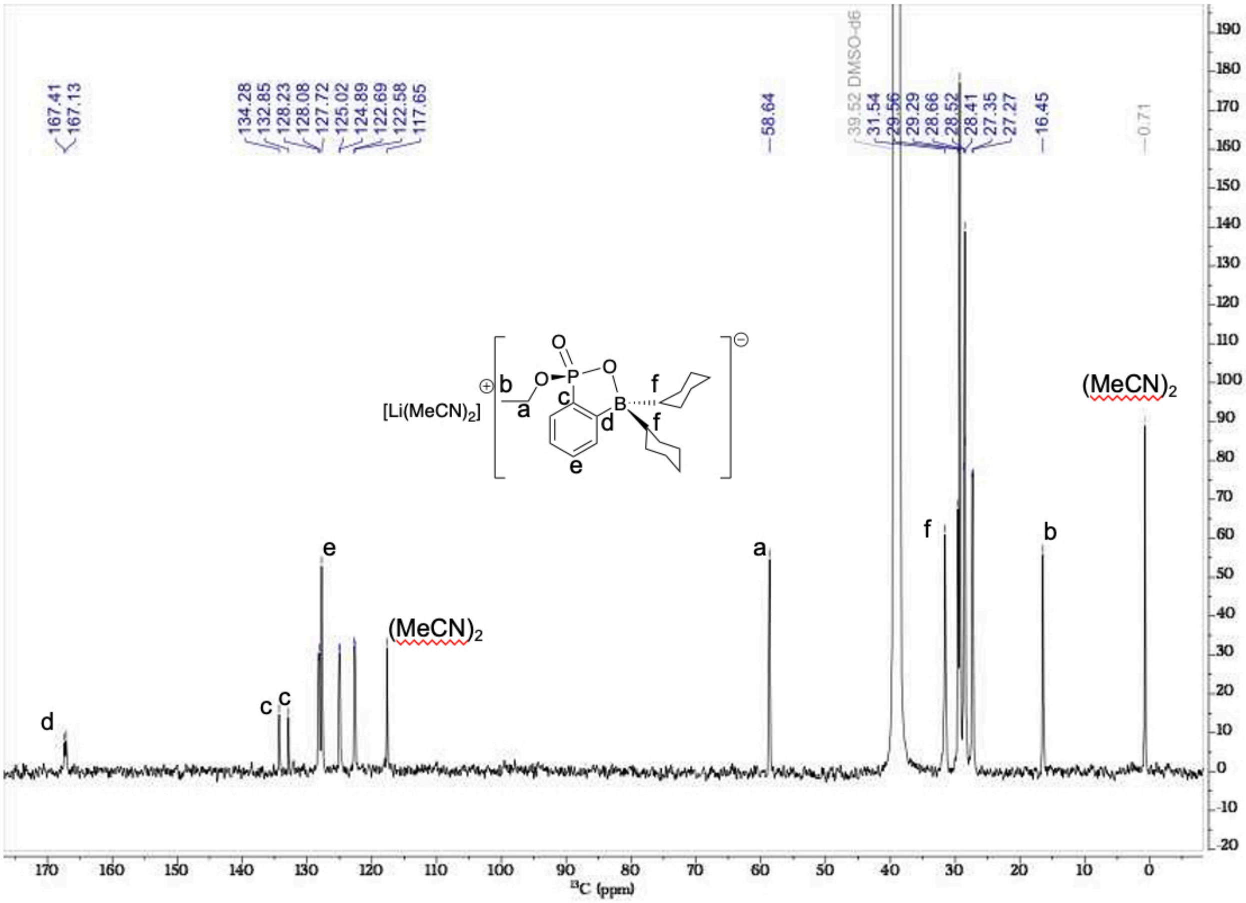

Figure S9. $^{13}C\{^{1}H\}$ NMR (126 MHz, DMSO-d_6) spectrum of $[Li(MeCN)_2]$[**3**].

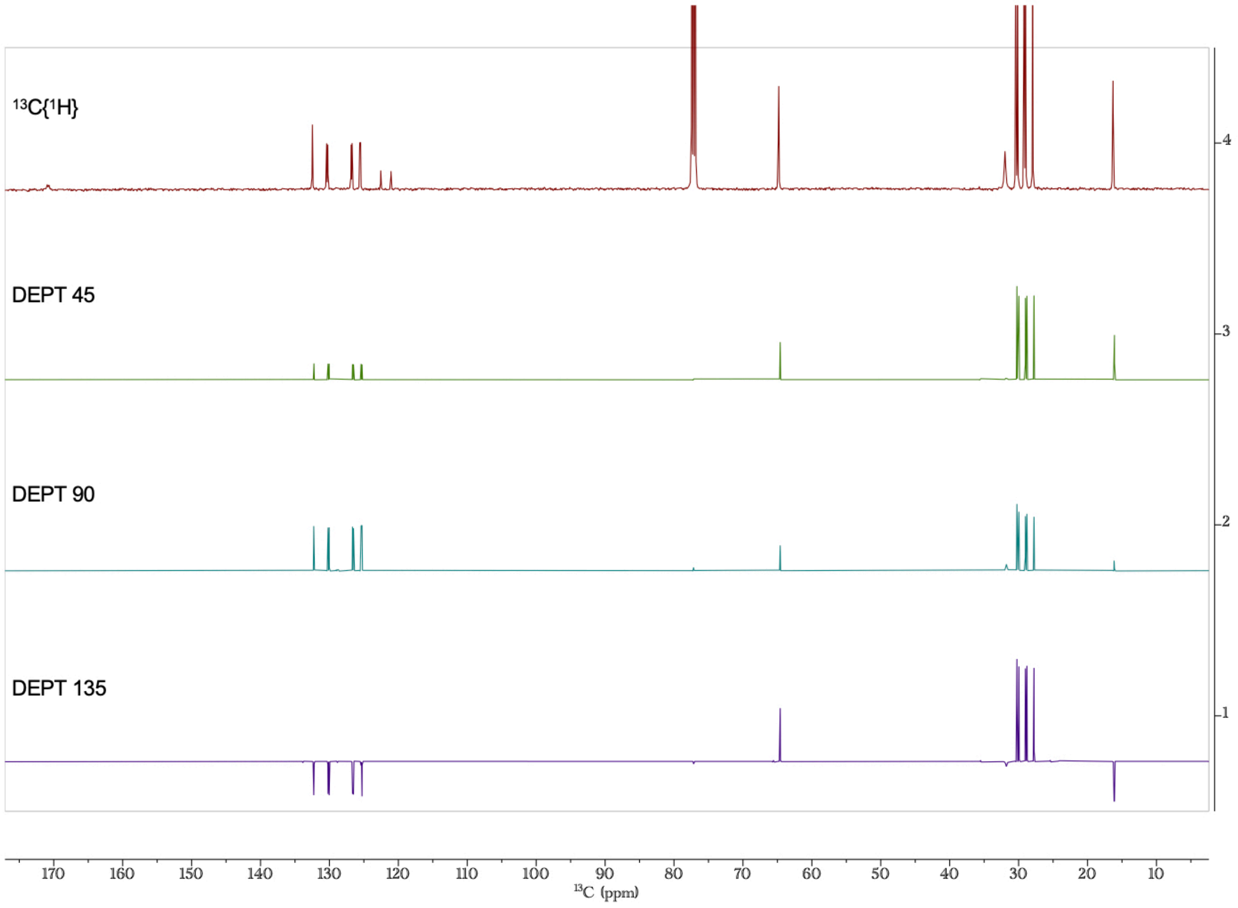

Figure S10. Stacked $^{13}C\{^1H\}$, DEPT 45, DEPT 90, and DEPT 135 (126MHz, $CDCl_3$) spectra of **2a**.

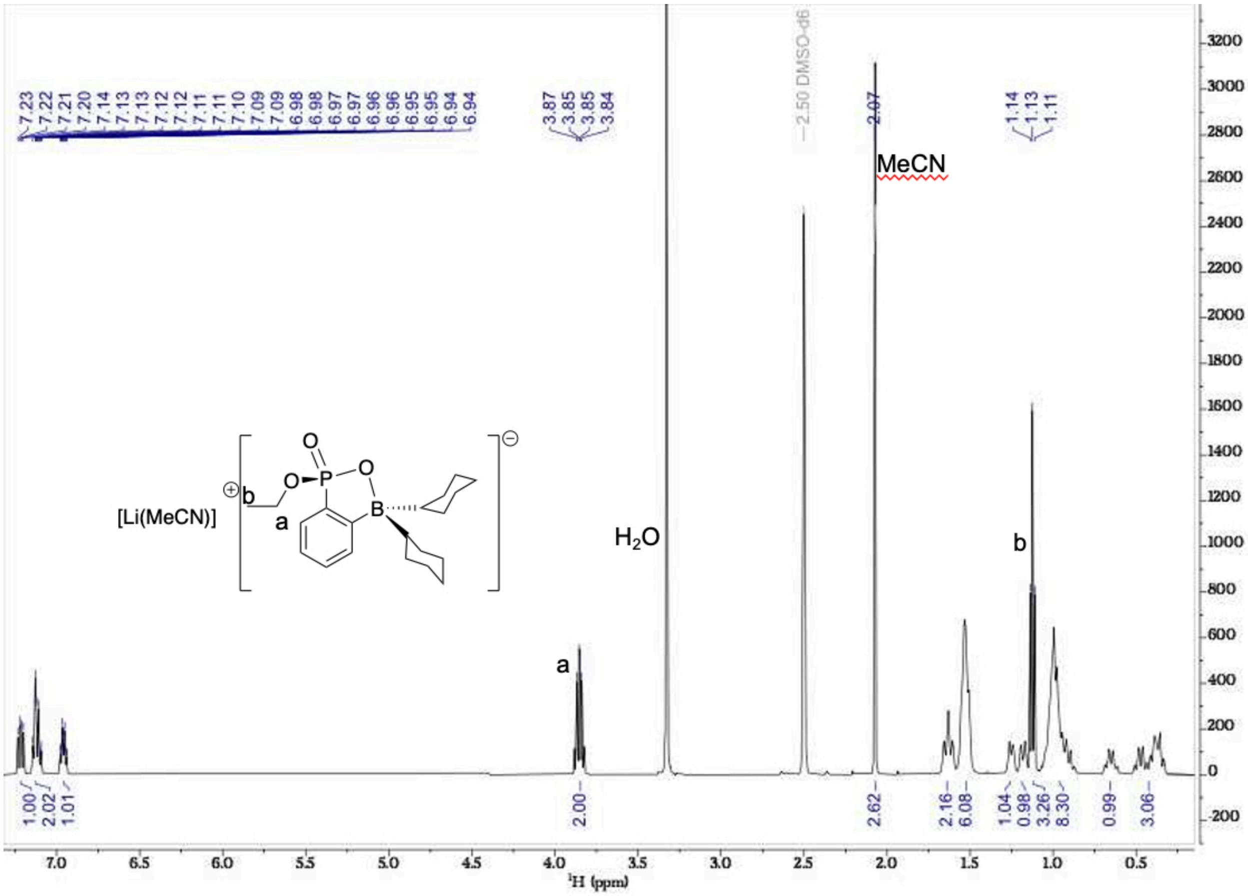

Figure S11. ^1H NMR (500 MHz, DMSO-d$_6$), spectrum of [Li(MeCN)][**3**].

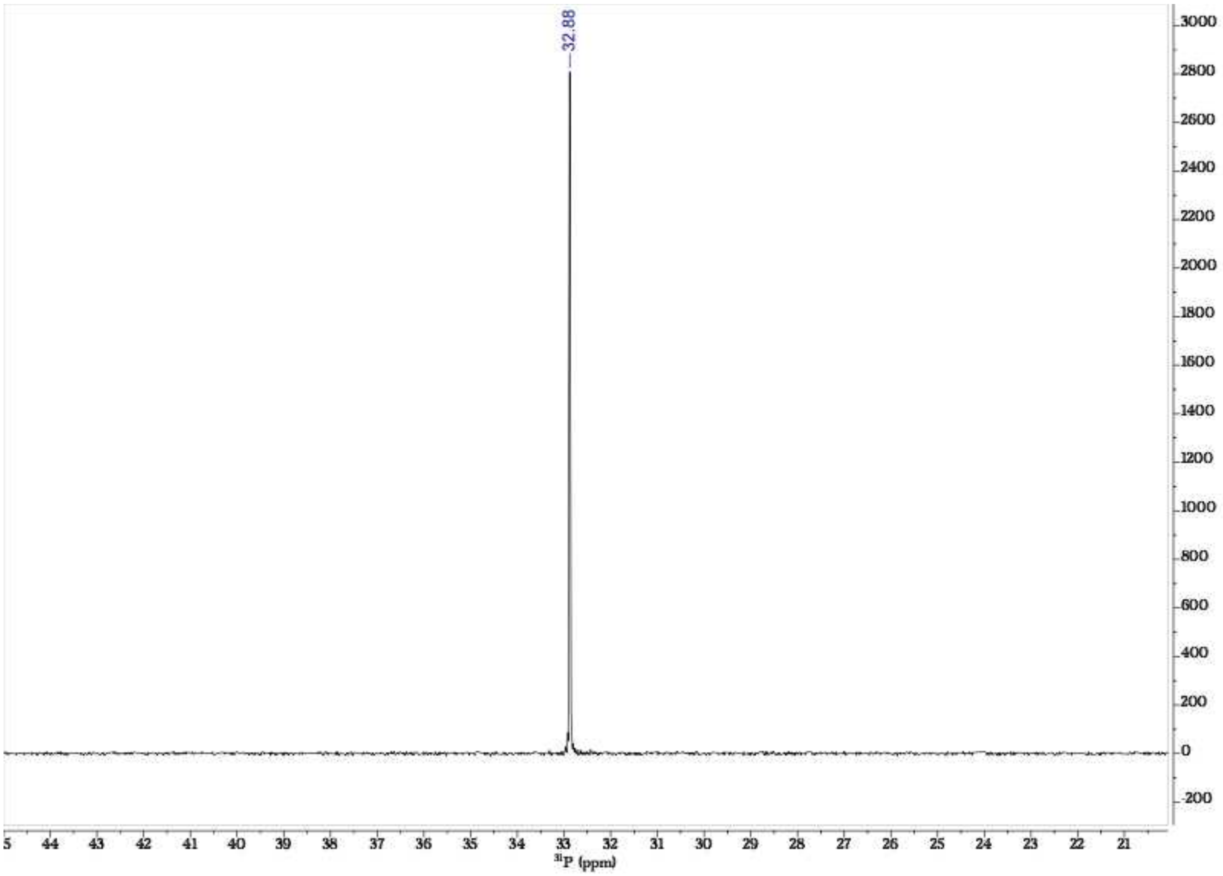

Figure S12. $^{31}P\{^{1}H\}$ NMR (202 MHz, DMSO-d_6) spectrum of [Li(MeCN)][**3**].

.

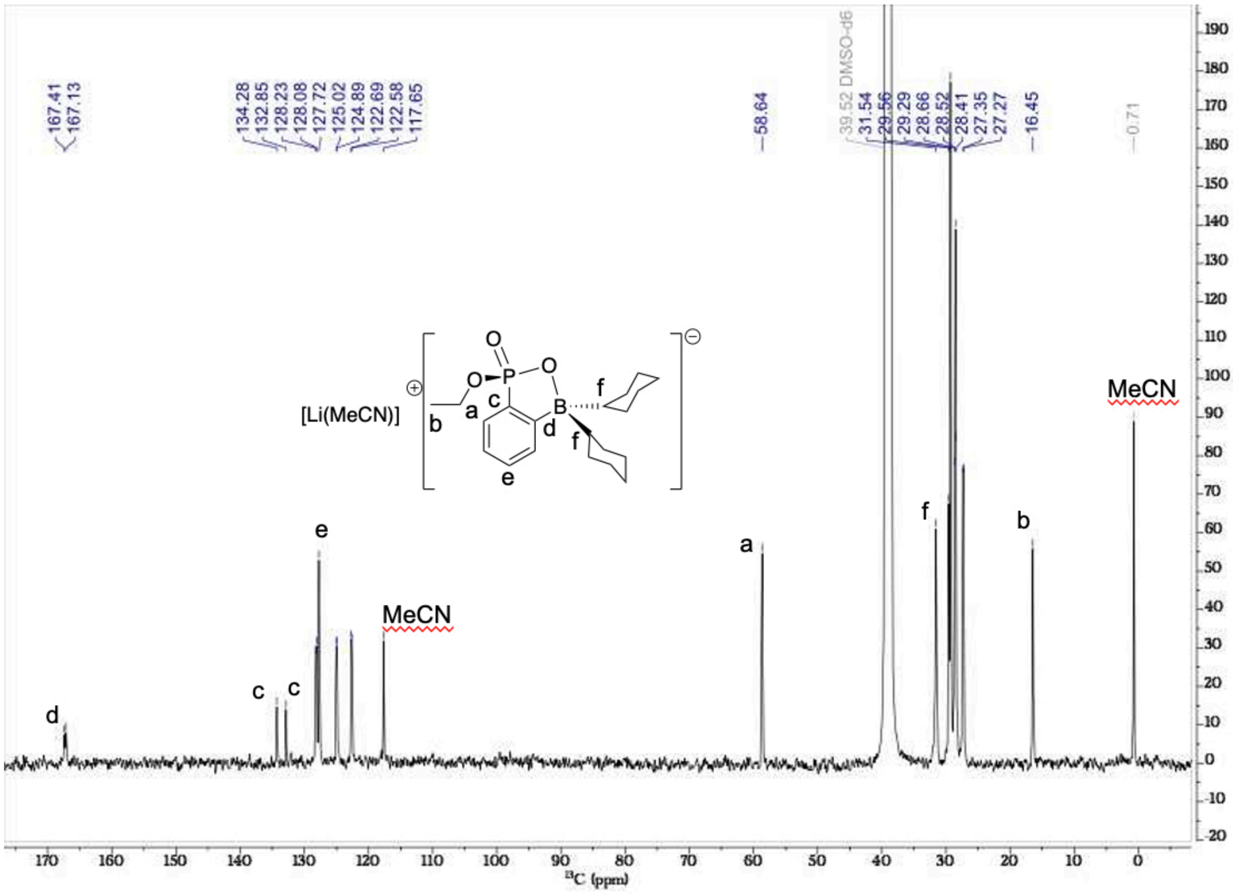

Figure S13. $^{13}C\{^1H\}$ NMR (202 MHz, DMSO-d$_6$) spectrum of [Li(MeCN)][**3**].

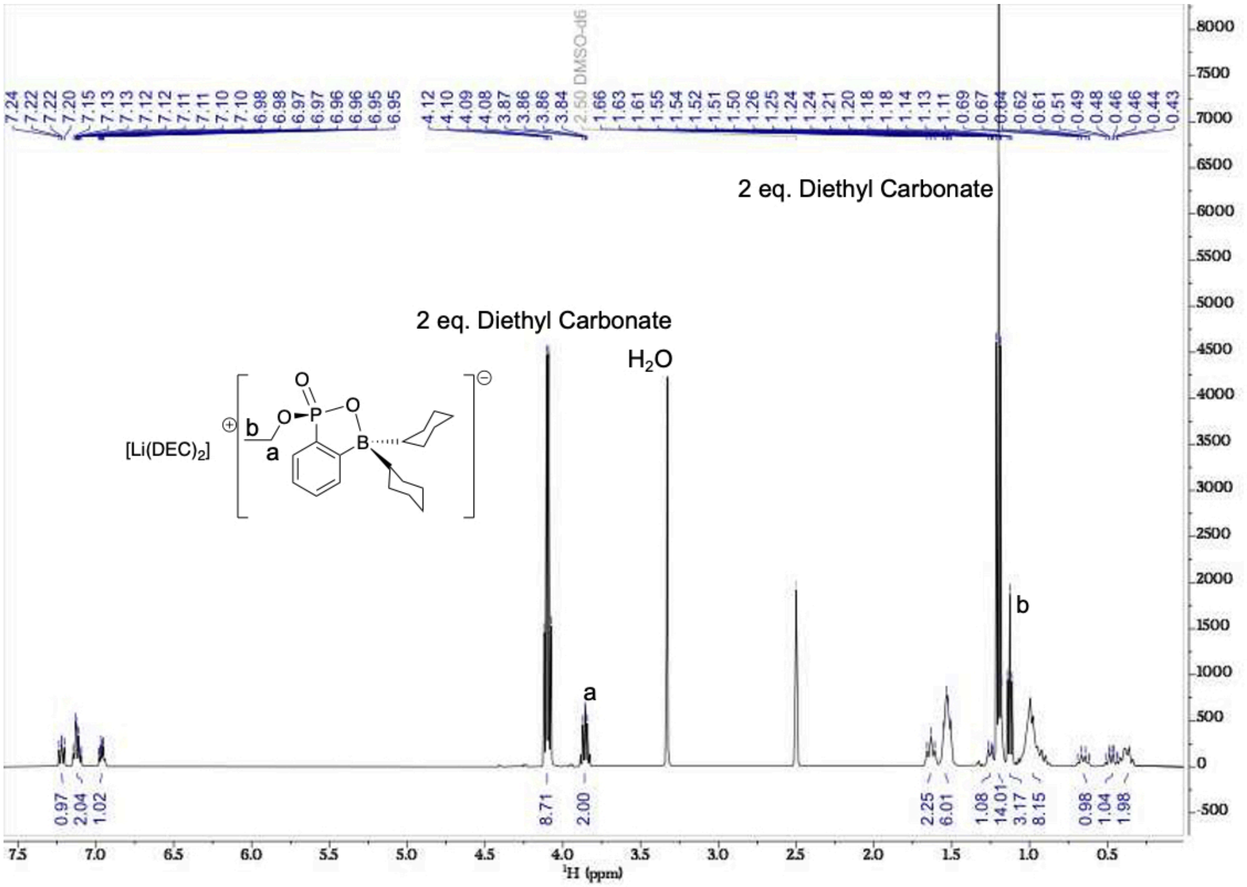

Figure S14. ^{1}H NMR (500 MHz, DMSO-d$_6$) spectrum of [Li(DEC)$_2$][**3**].

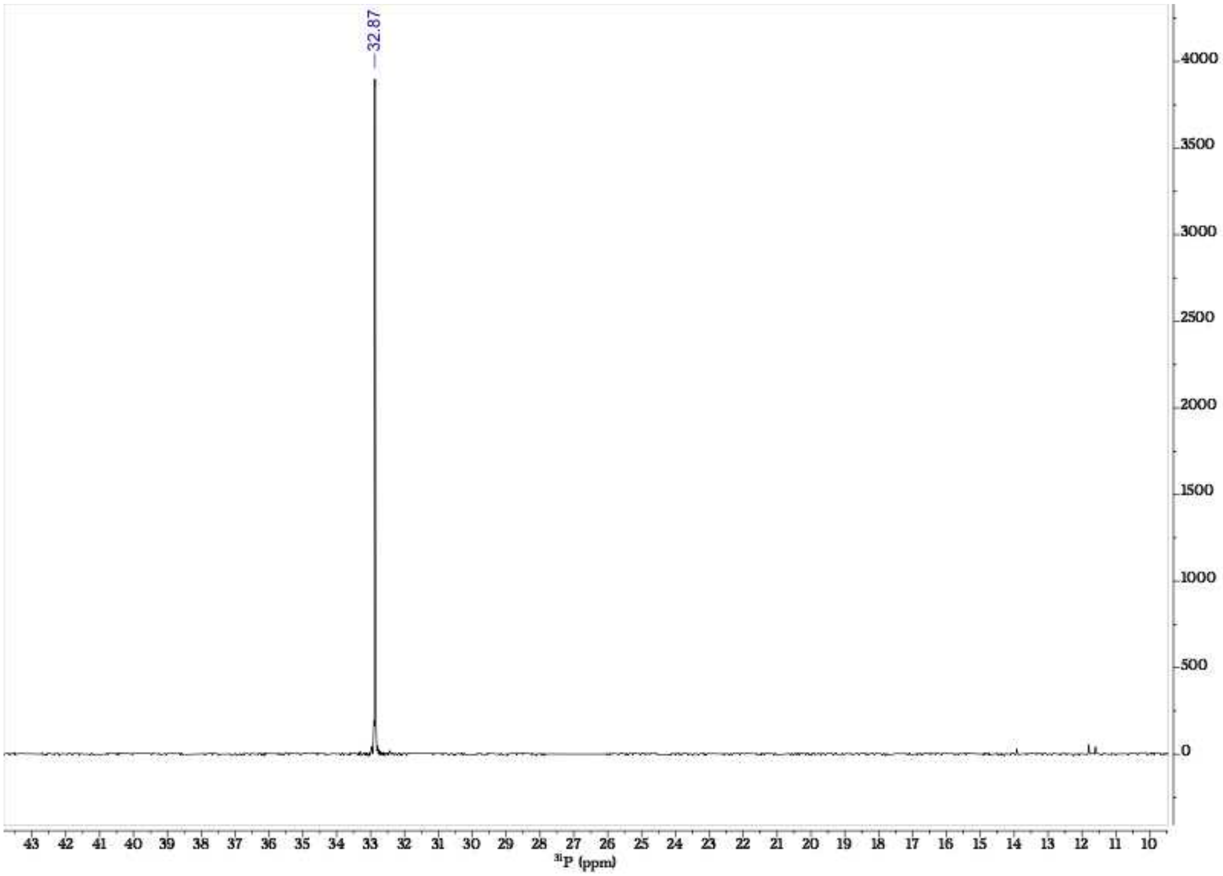

Figure S15. $^{31}P\{^{1}H\}$ NMR (202 MHz, DMSO-d$_6$) spectrum of [Li(DECC)$_2$][**3**].

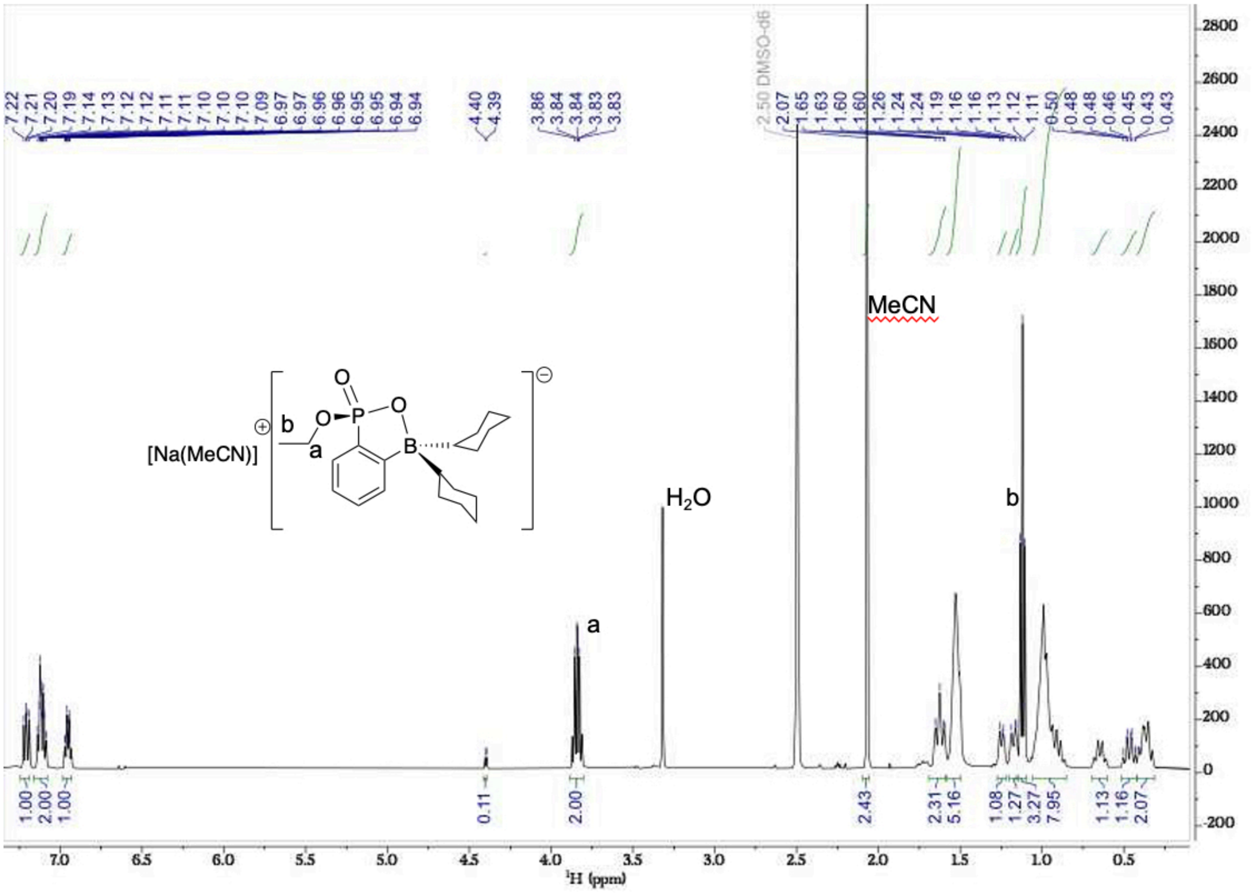

Figure S16. ^{1}H NMR (500 MHz, DMSO-d_6) spectrum of [Na(MeCN)][**3**].

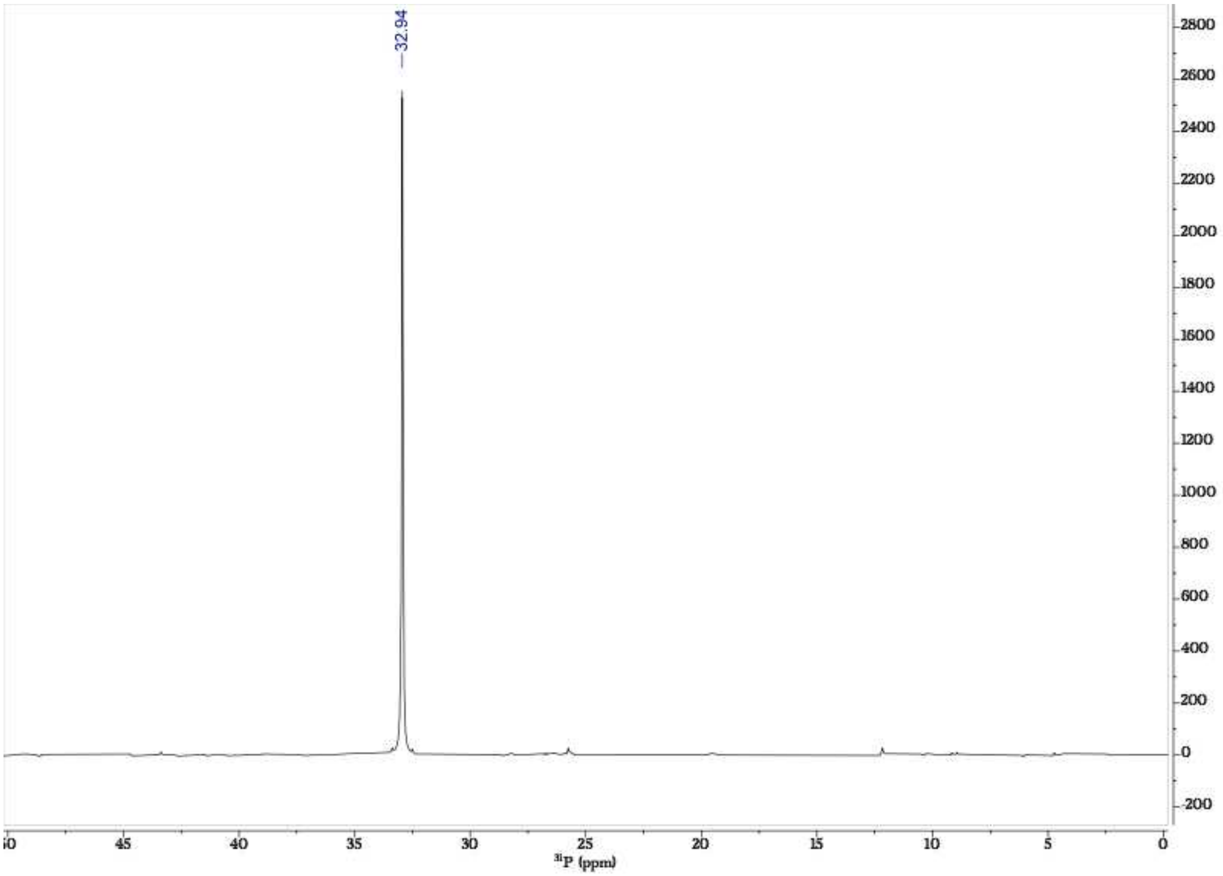

Figure S17. $^{31}P\{^1H\}$ NMR (202 MHz, DMSO-d$_6$) spectrum of [Na(MeCN)][**3**].

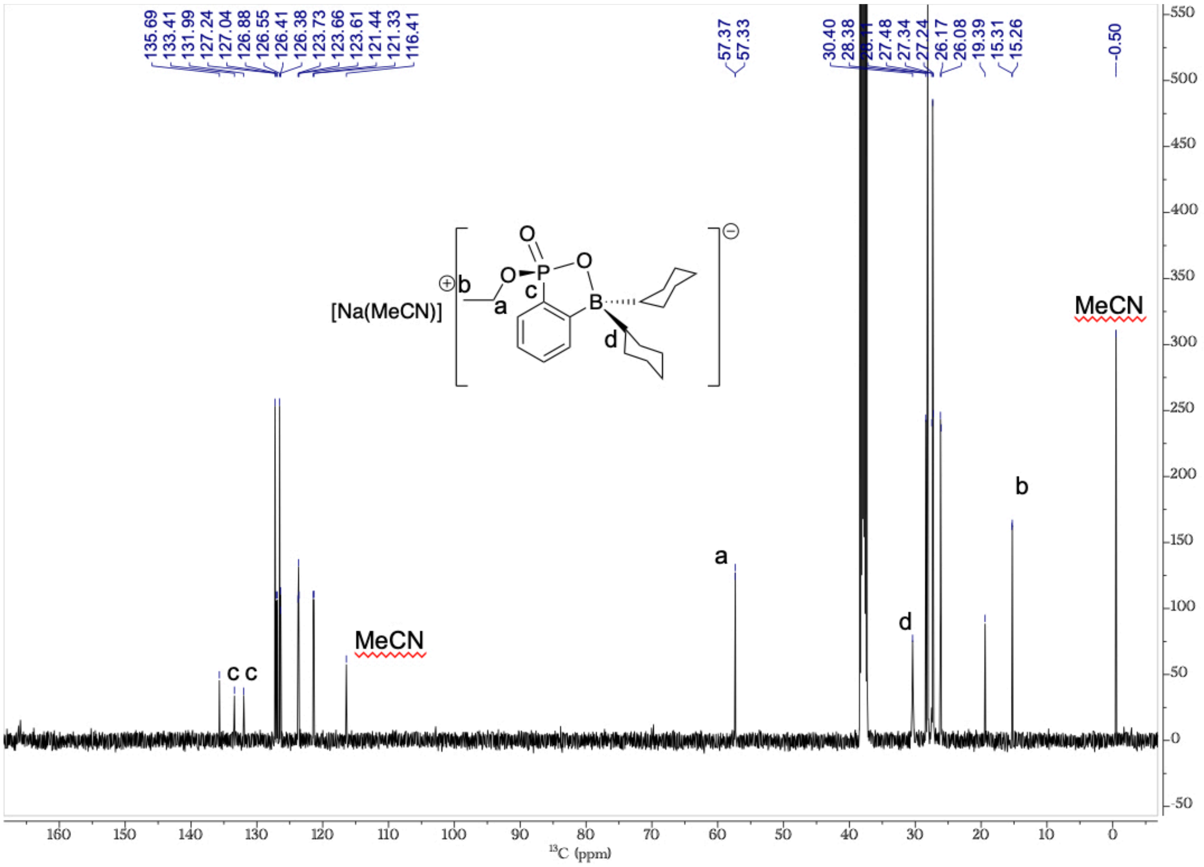

Figure S18. $^{13}C\{^{1}H\}$ (126 MHz, DMSO-d$_6$) spectrum of [Na(MeCN)][**3**].

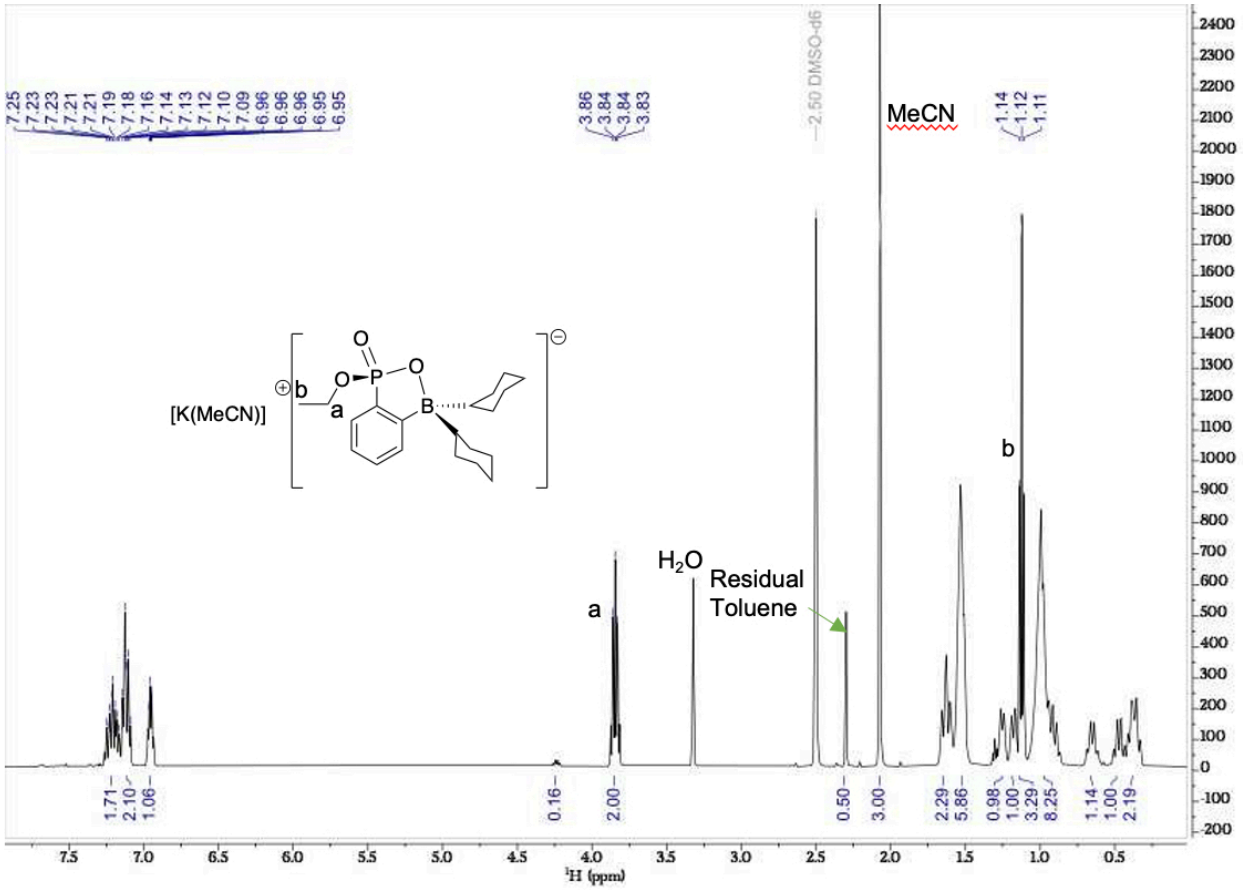

Figure S19. ^{1}H NMR (500 MHz, DMSO-d$_6$) spectrum of [K(MeCN)][**3**].

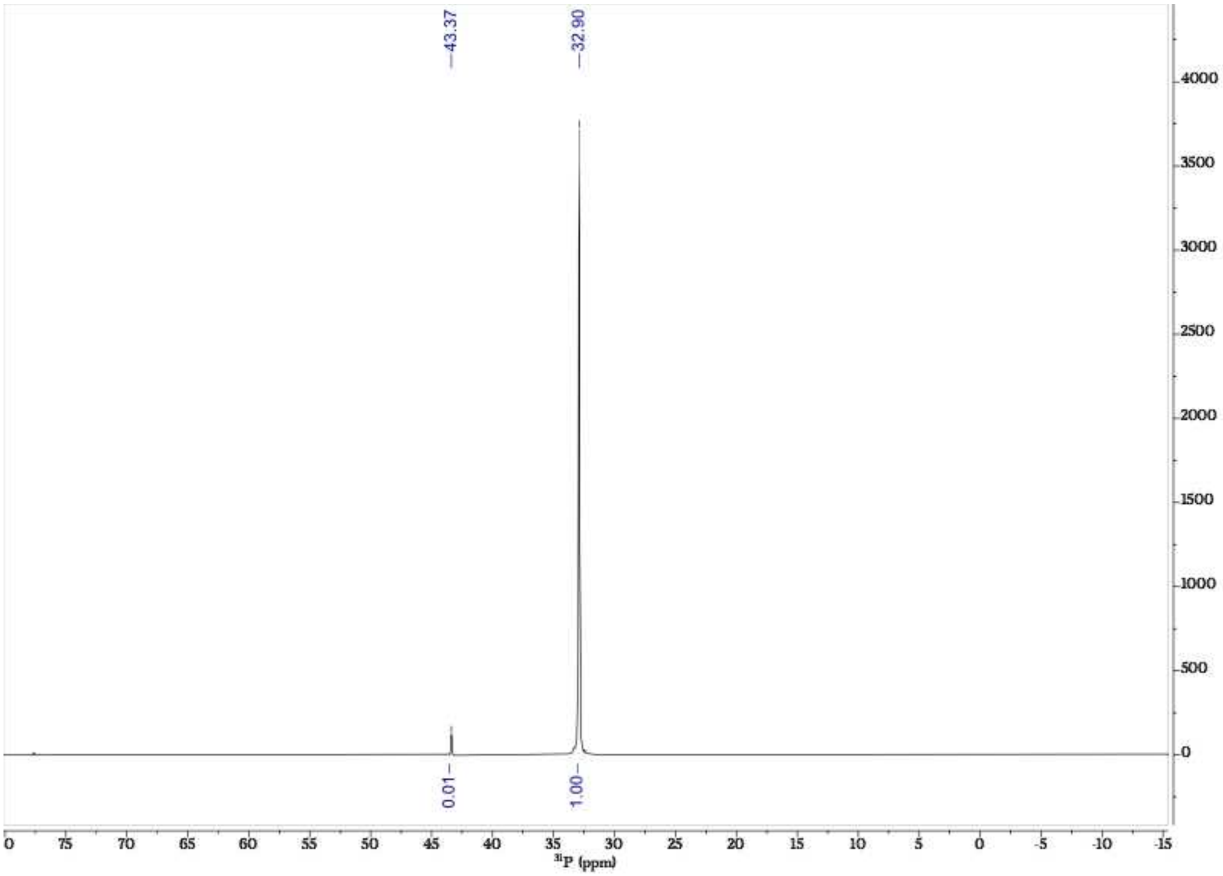

Figure S20. $^{31}P\{^{1}H\}$ NMR (202 MHz, DMSO-d_6) spectrum of [K(MeCN)][**3**].

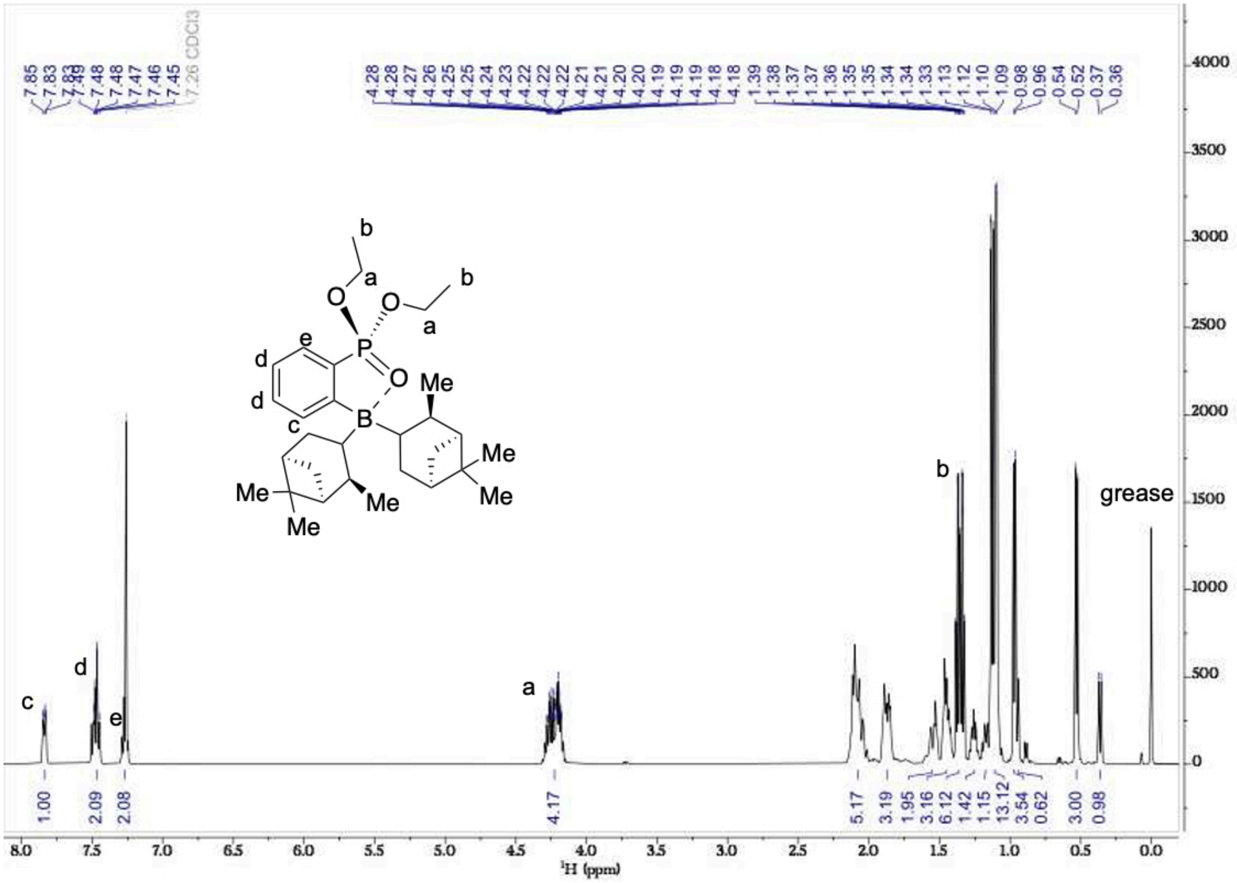

Figure S21. ^{1}H NMR (500 MHz, Chloroform-d) spectrum of **2b**.

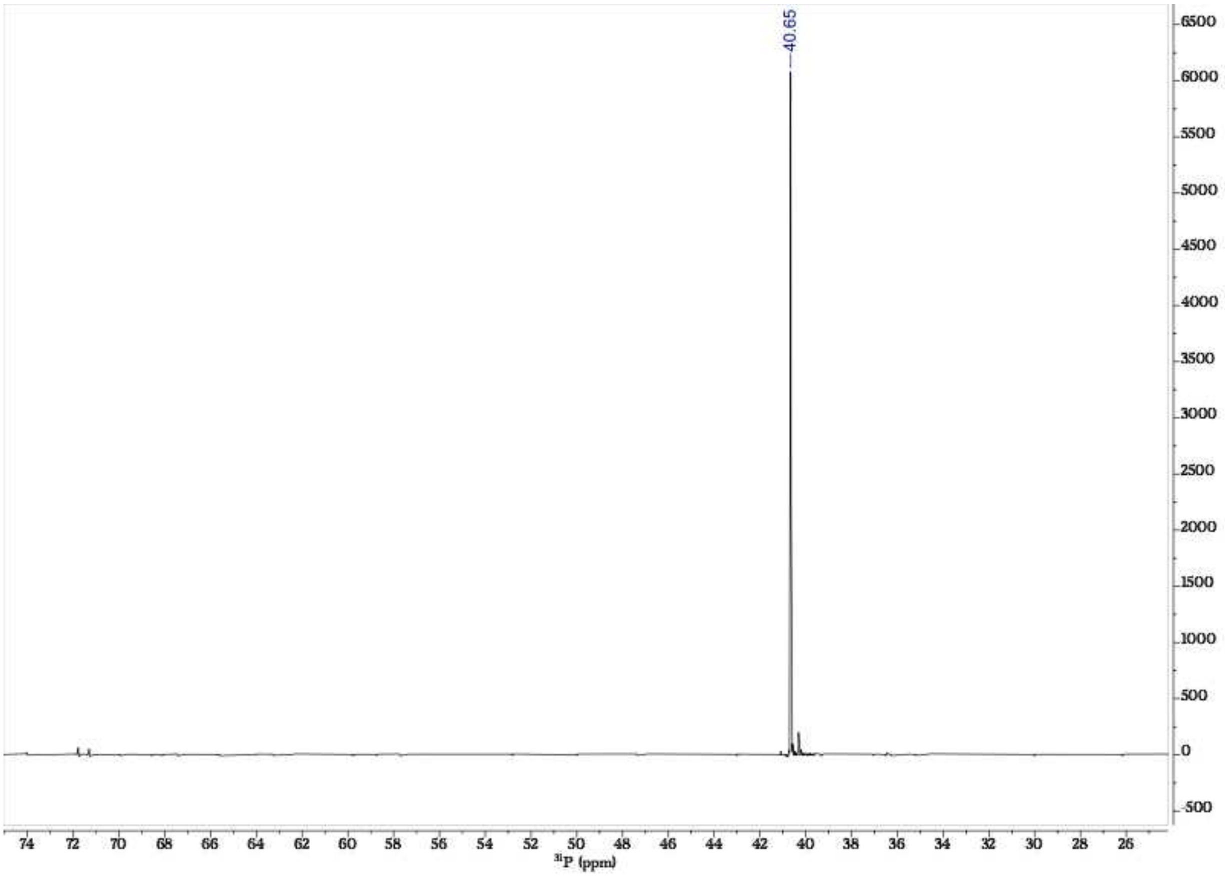

Figure S22. $^{31}P\{^{1}H\}$ NMR (202 MHz, Chloroform-d) spectrum of **2b**.

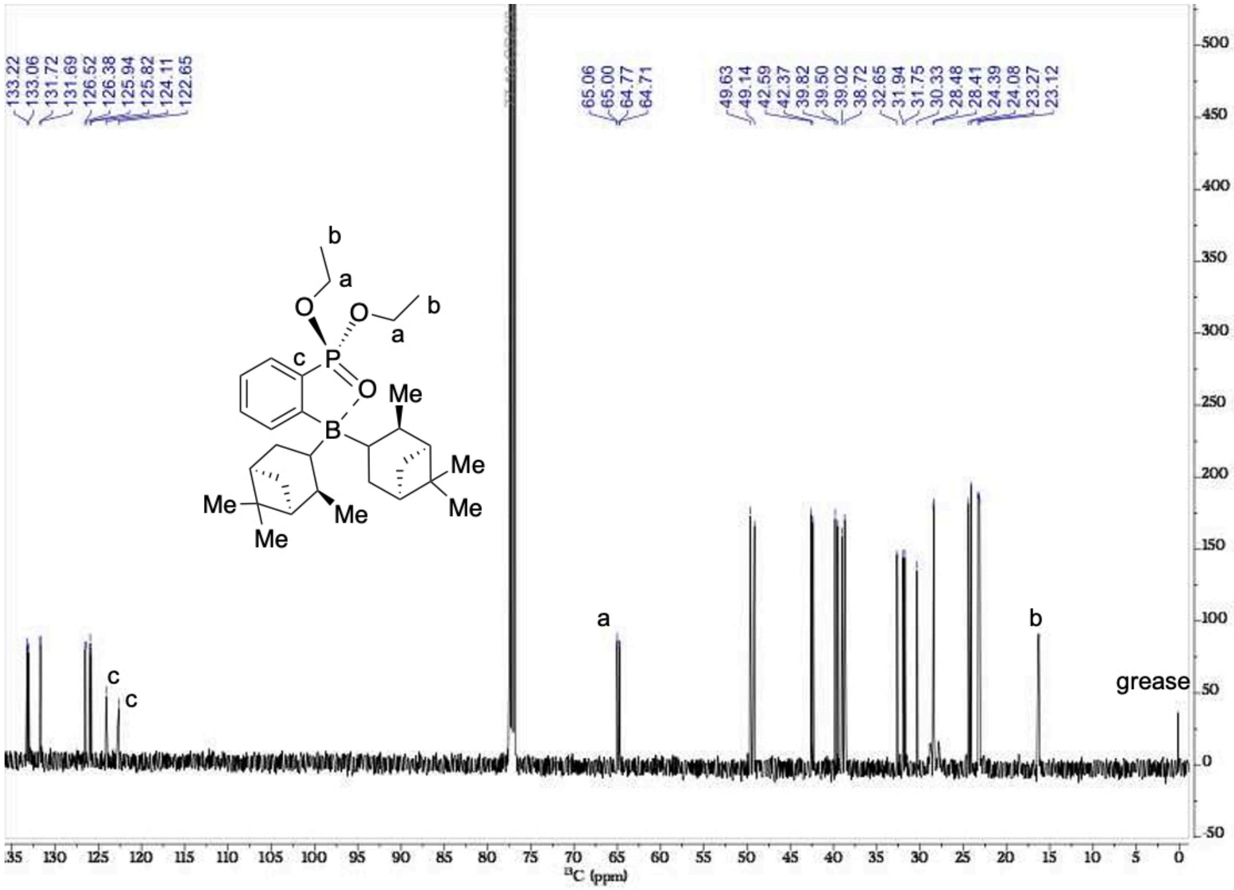

Figure S23. $^{13}C\{^{1}H\}$ NMR (126 MHz, Chloroform-d) spectrum of **2b**.

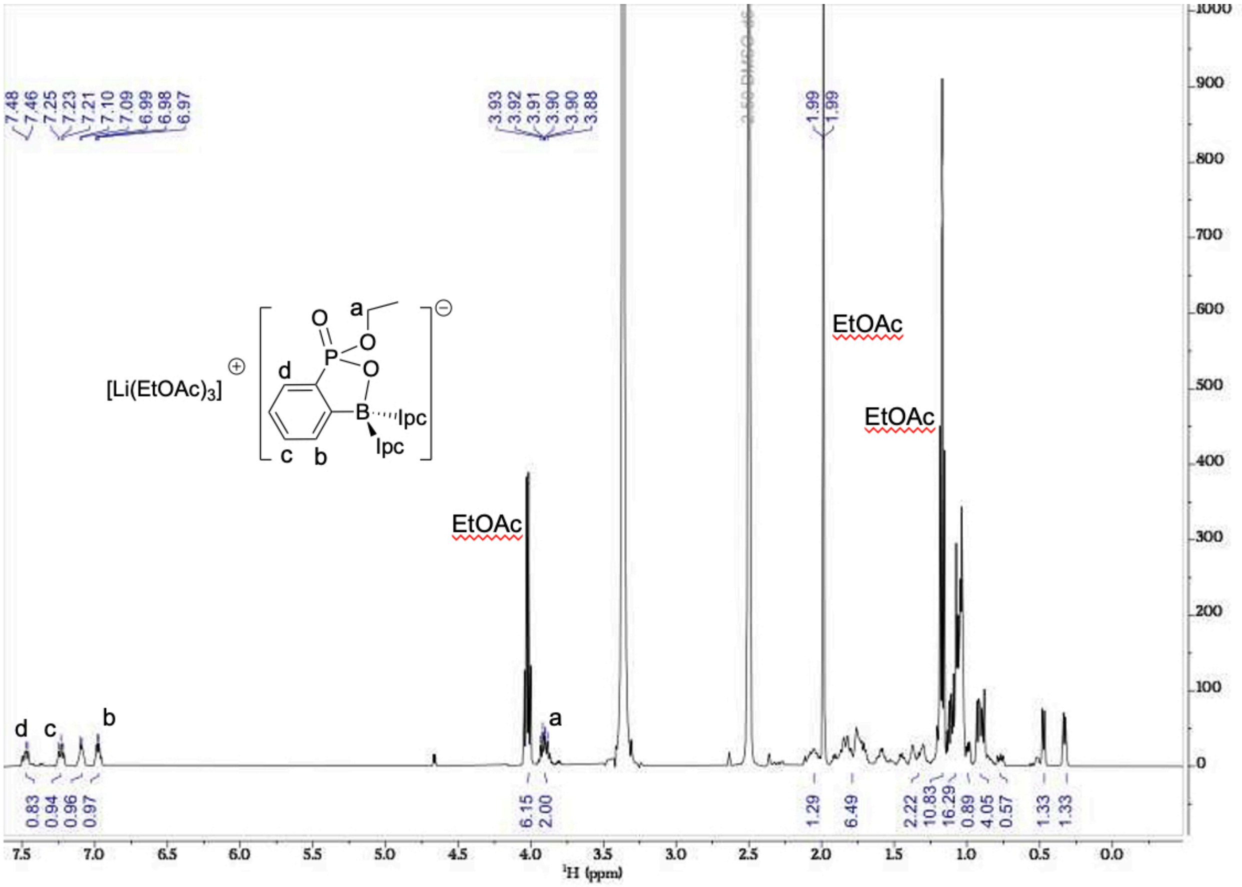

Figure S24. ^{1}H NMR (500 MHz, DMSO-d$_6$) spectrum of [Li(EtOAc)$_3$][**4**].

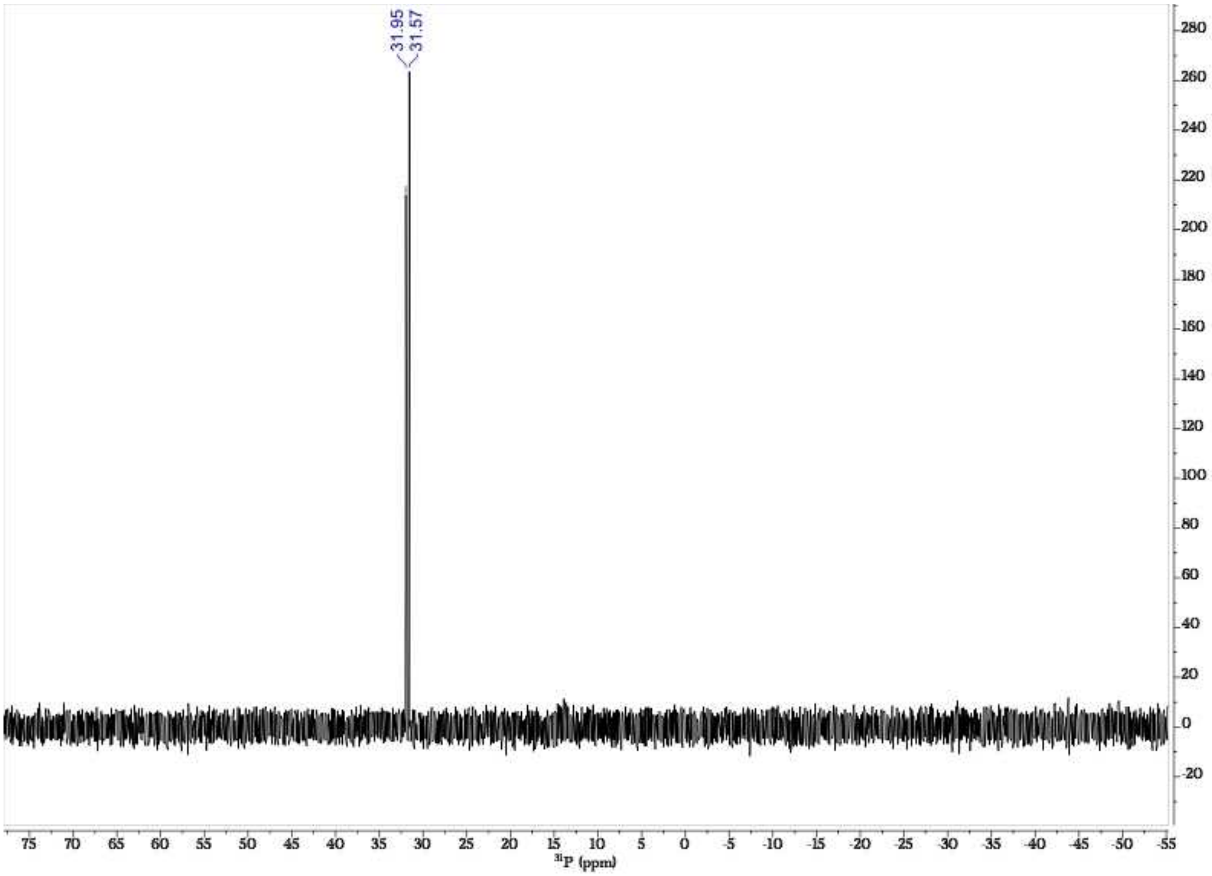

Figure S25. $^{31}P\{^{1}H\}$ NMR (202 MHz, DMSO-d_6) spectrum of [Li(EtOAc)$_3$][**4**].

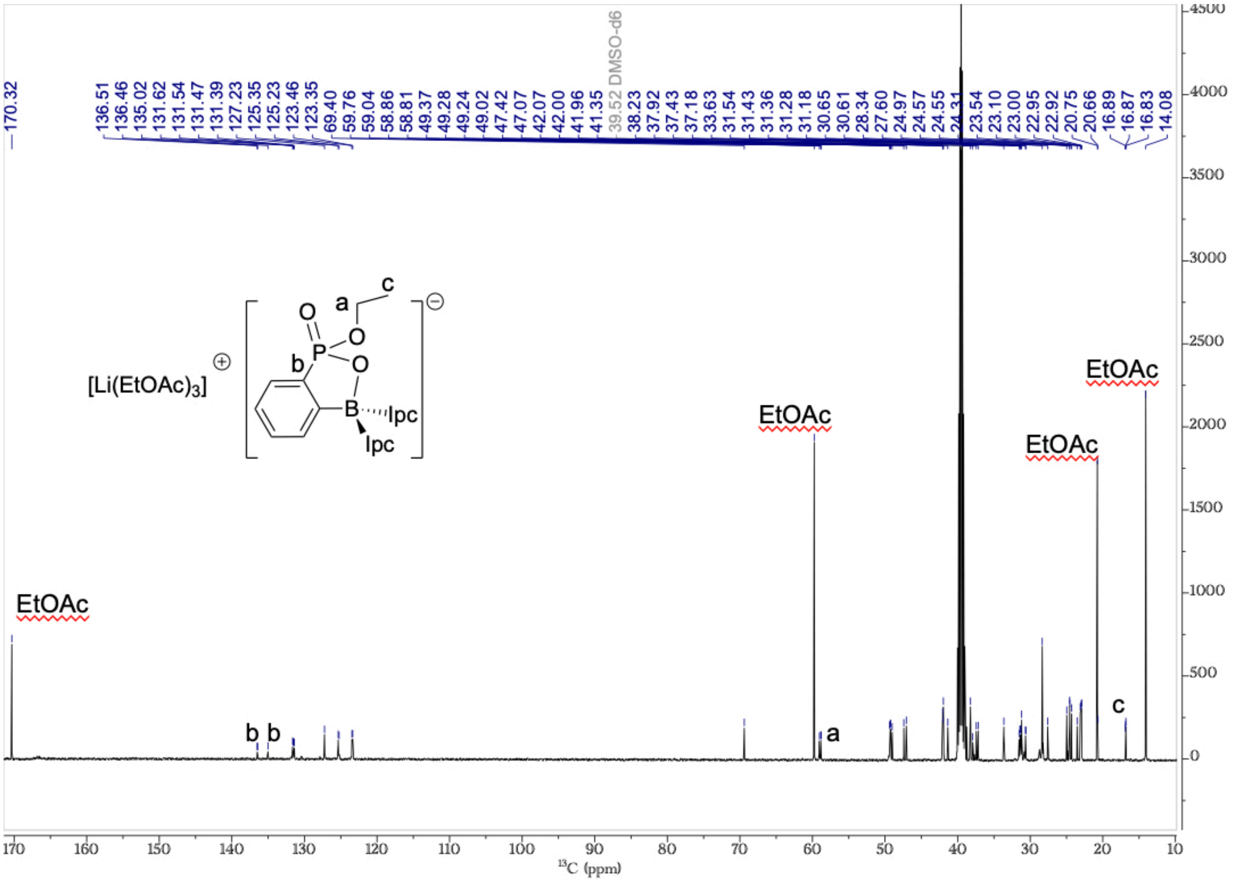

Figure S26. $^{13}C\{^{1}H\}$ NMR (126 MHz, DMSO-d$_6$) spectrum of [Li(EtOAc)$_3$][**4**].

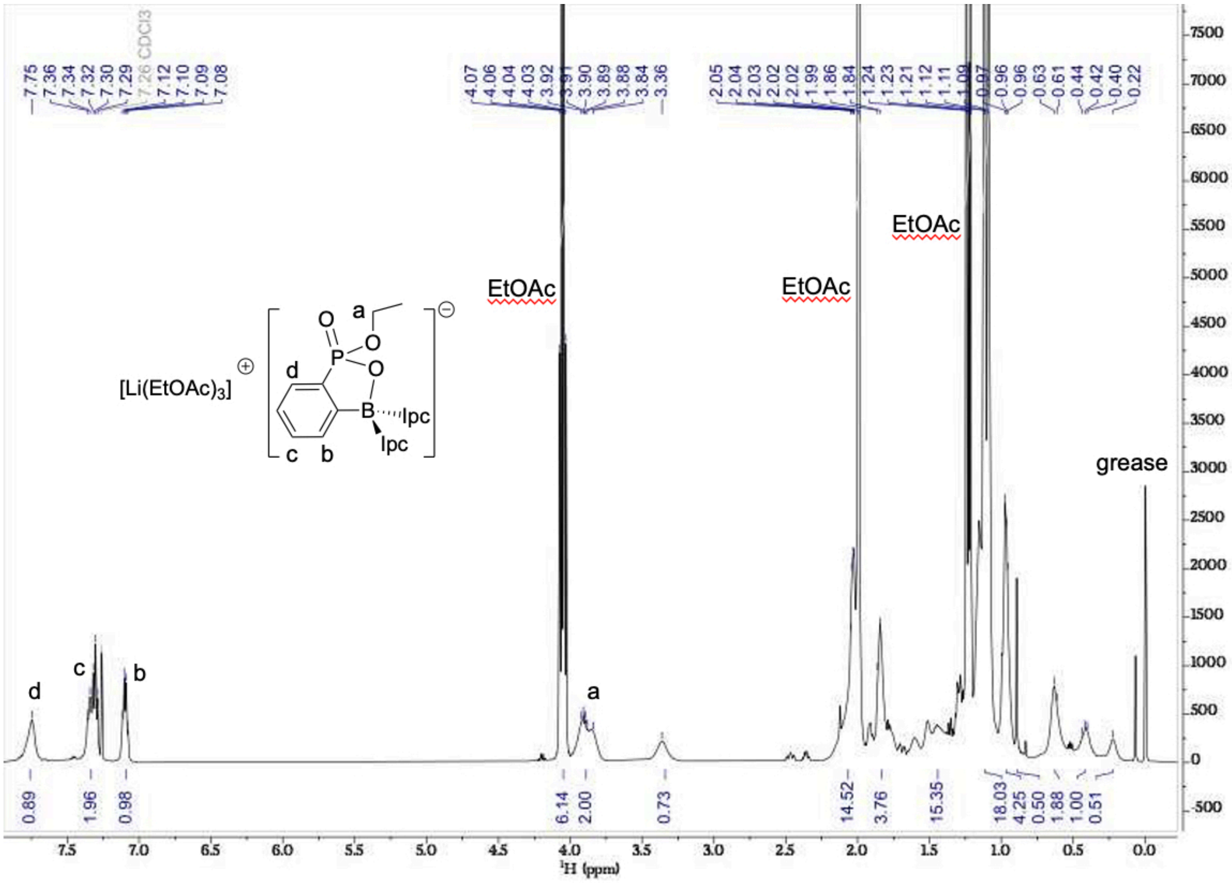

Figure S27. ¹H NMR (500 MHz, $CDCl_3$-d) spectrum of [Li(EtOAc)$_3$][**4**].

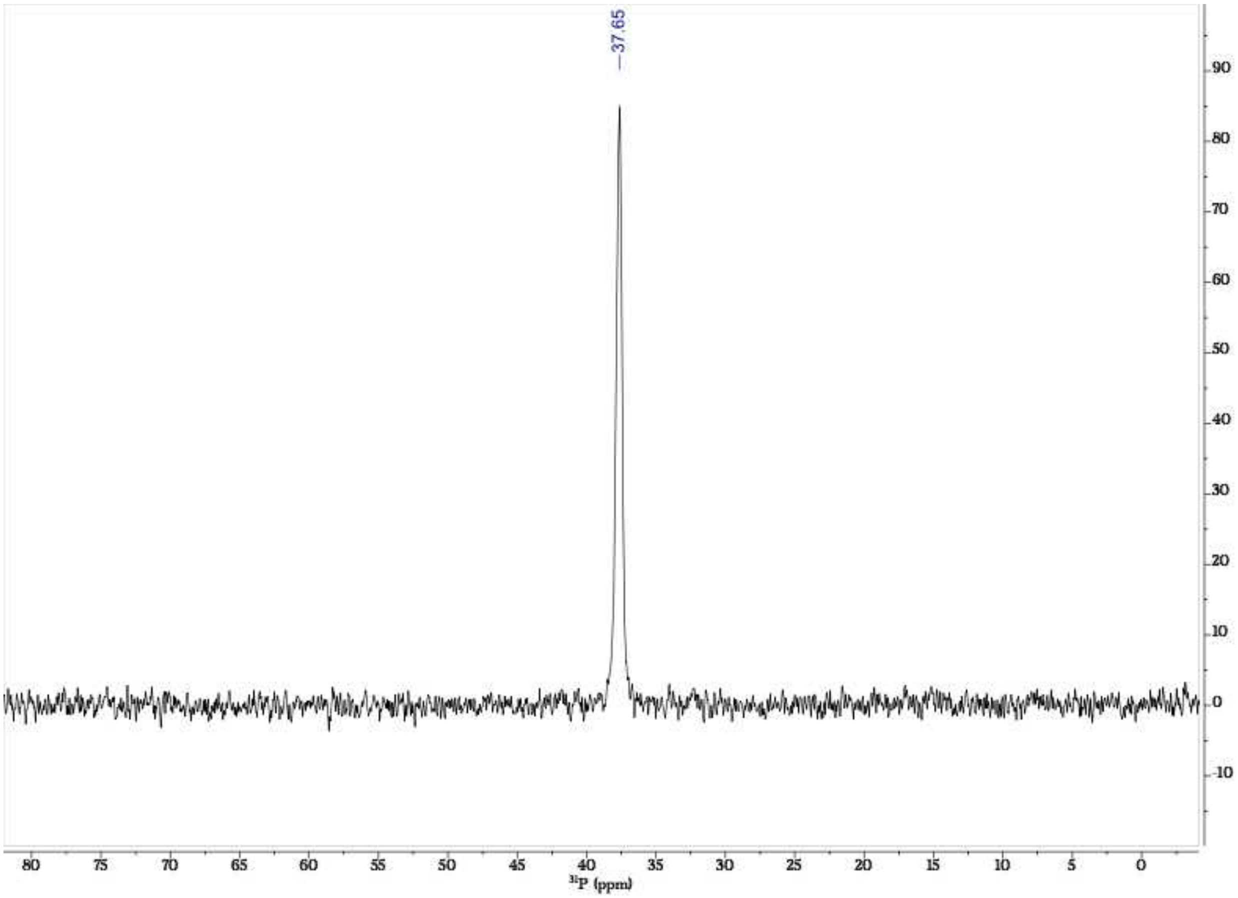

Figure S28. $^{31}P\{^{1}H\}$ NMR (202 MHz, $CDCl_3$-d) spectrum of [Li(EtOAc)$_3$][**4**].

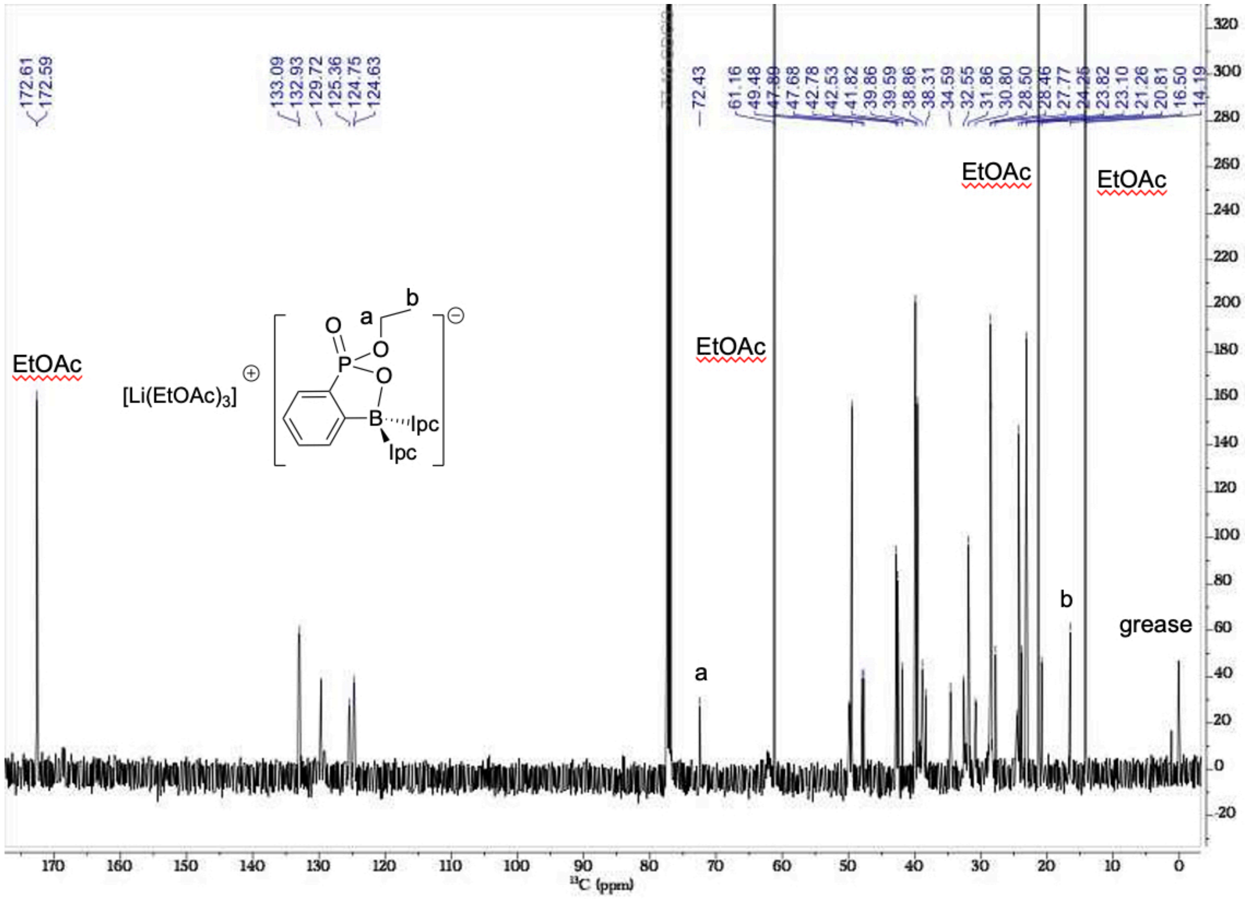

Figure S29. $^{13}C\{^{1}H\}$ NMR (126 MHz, $CDCl_3$-d) spectrum of [Li(EtOAc)$_3$][**4**].

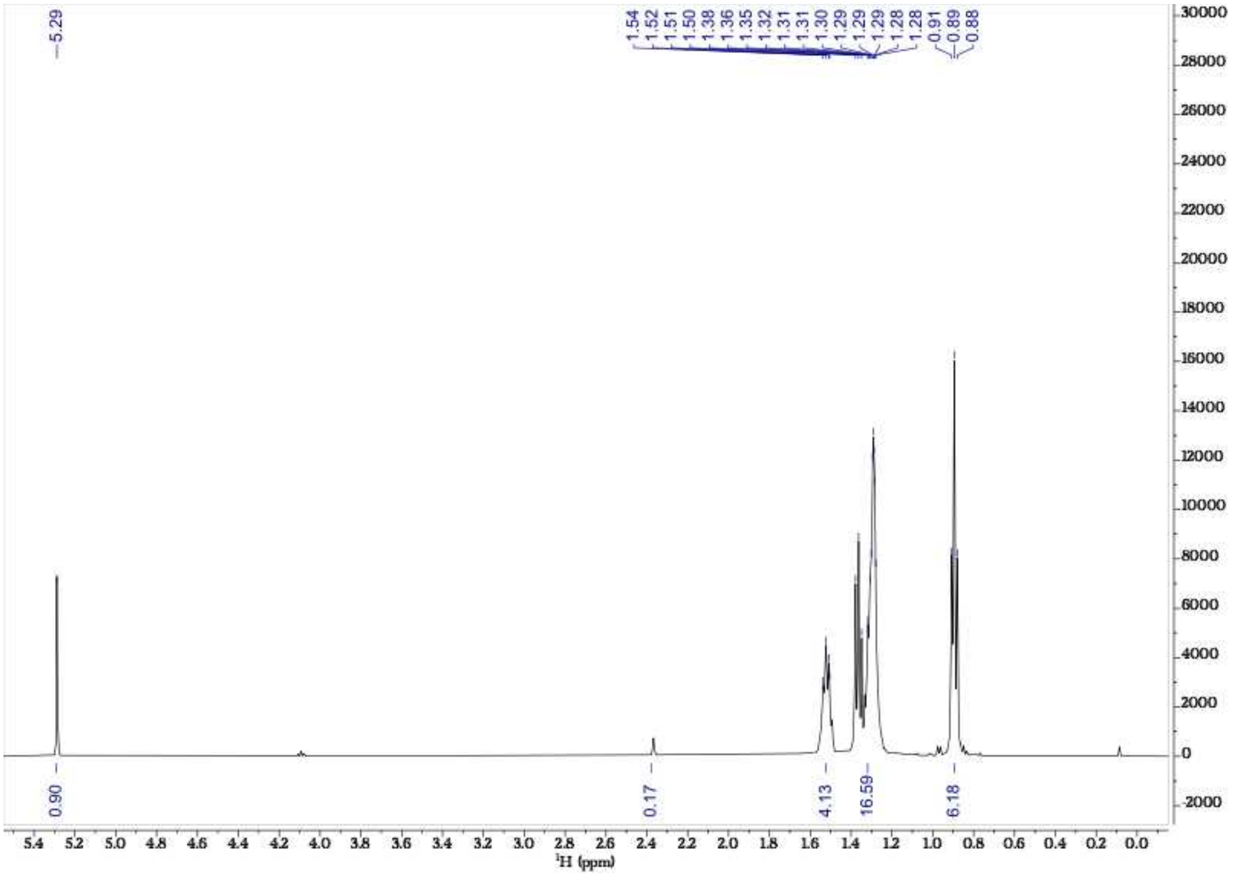

Figure S30. ^{1}H NMR (500 MHz, Chloroform-d) spectrum of dihexylchloroborane.

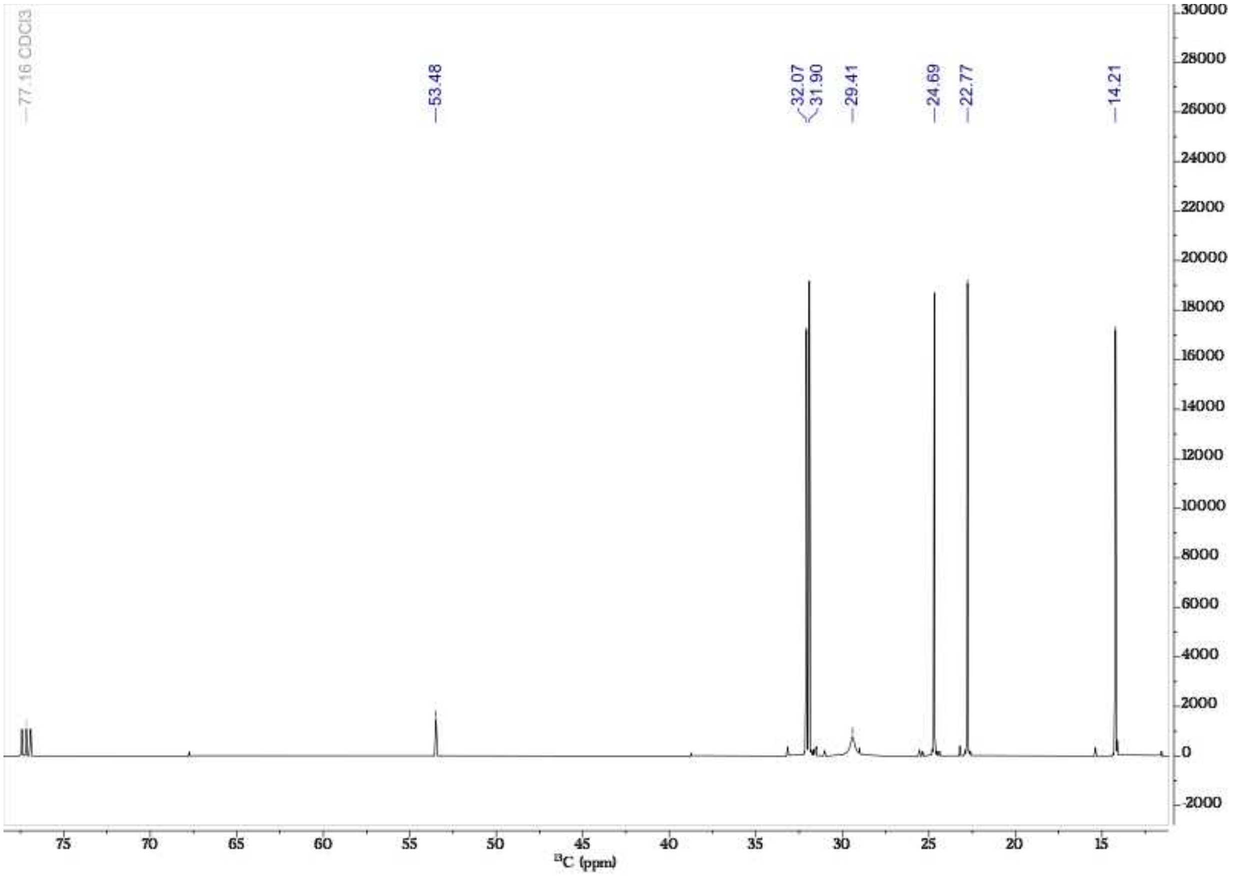

Figure S31. $^{13}C\{^{1}H\}$ NMR (126 MHz, Chloroform-d) spectrum of dihexylchloroborane.

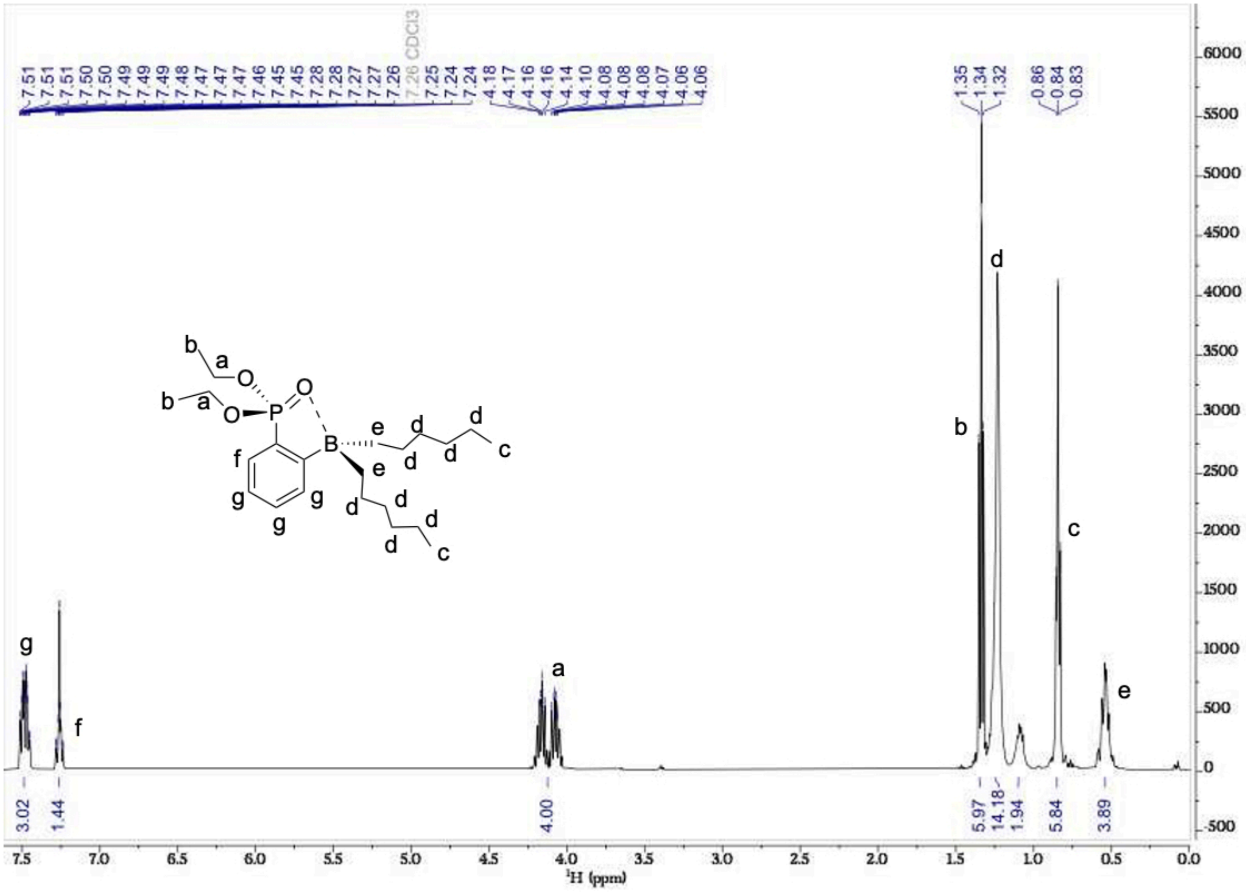

Figure S32. ^{1}H NMR (500 MHz, Chloroform-d) spectrum of **2c**.

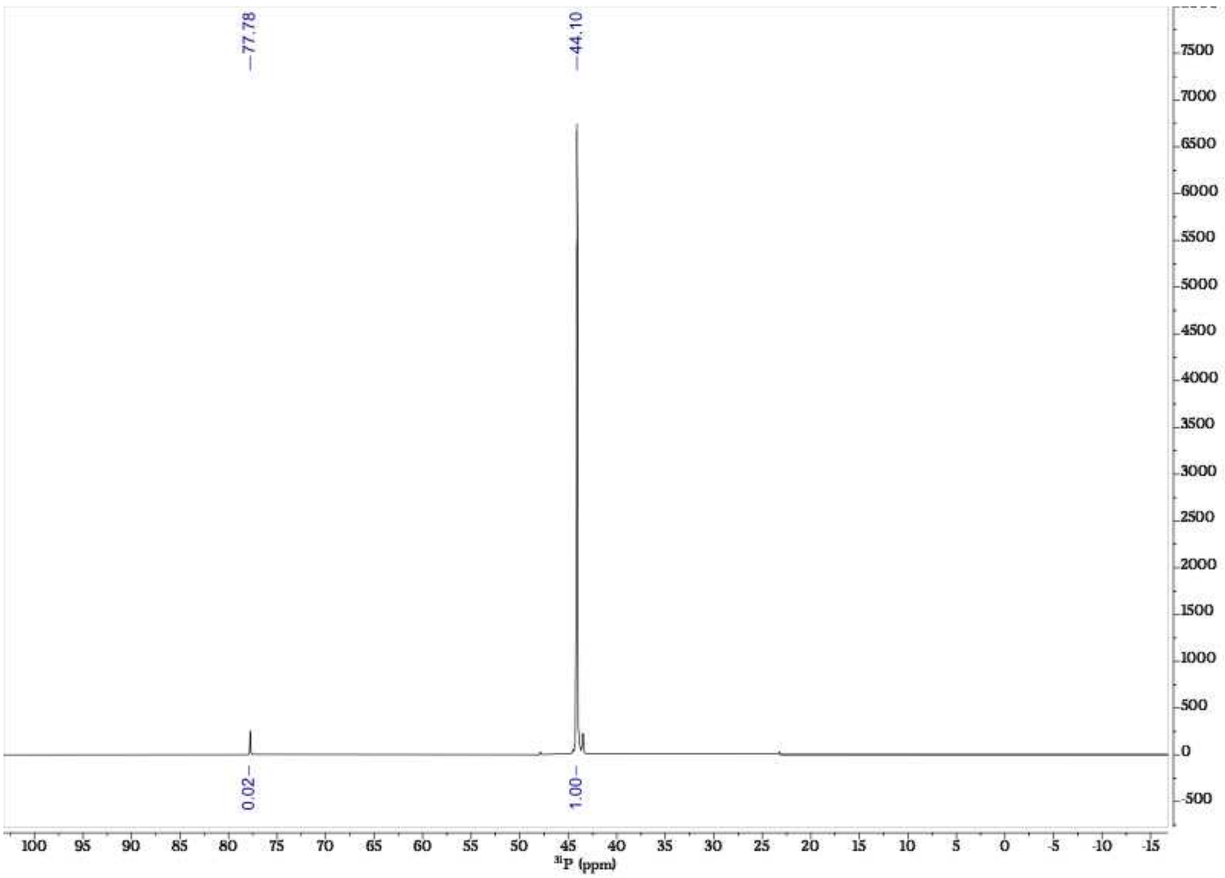

Figure S33. $^{31}P\{^1H\}$ NMR (202 MHz, Chloroform-d) spectrum of **2c**.

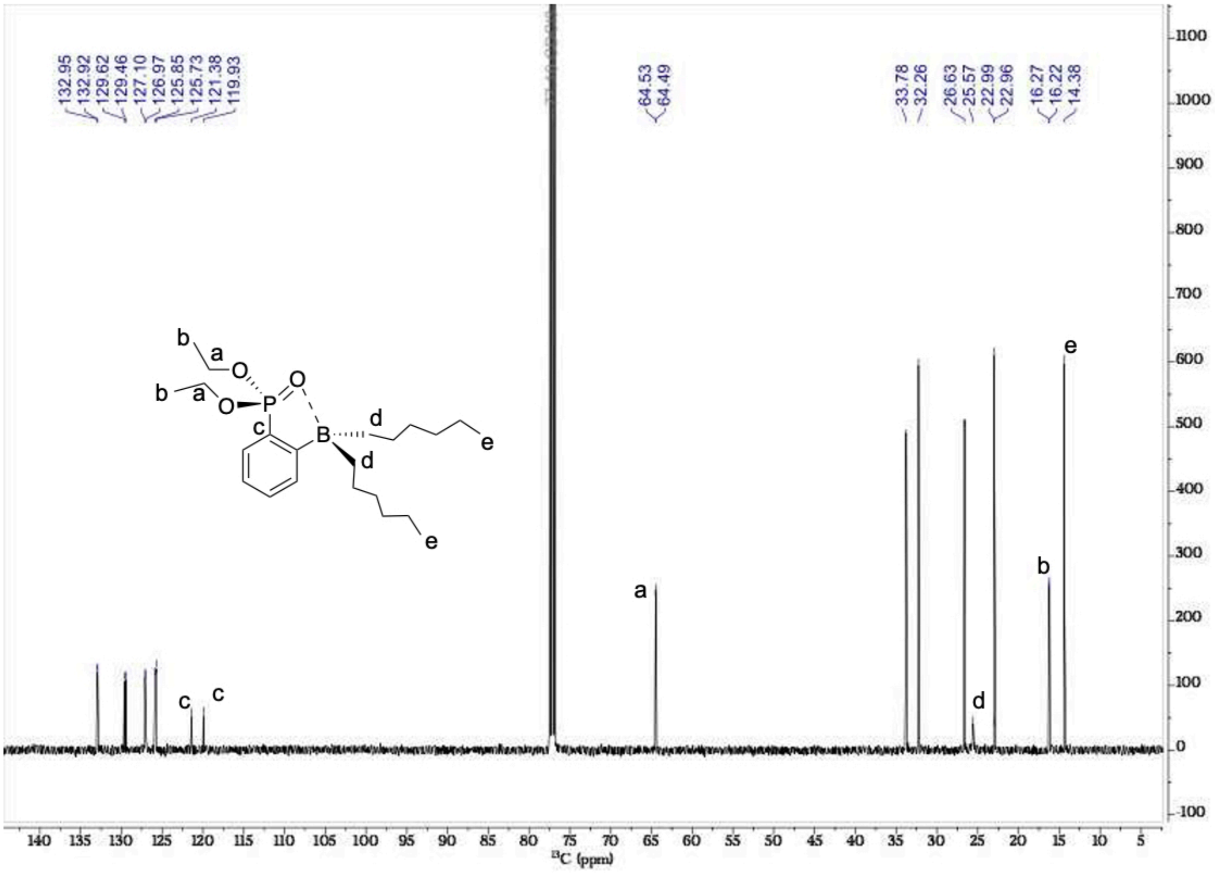

Figure S34. $^{13}C\{^{1}H\}$ NMR (126 MHz, Chloroform-d) spectrum of **2c**.

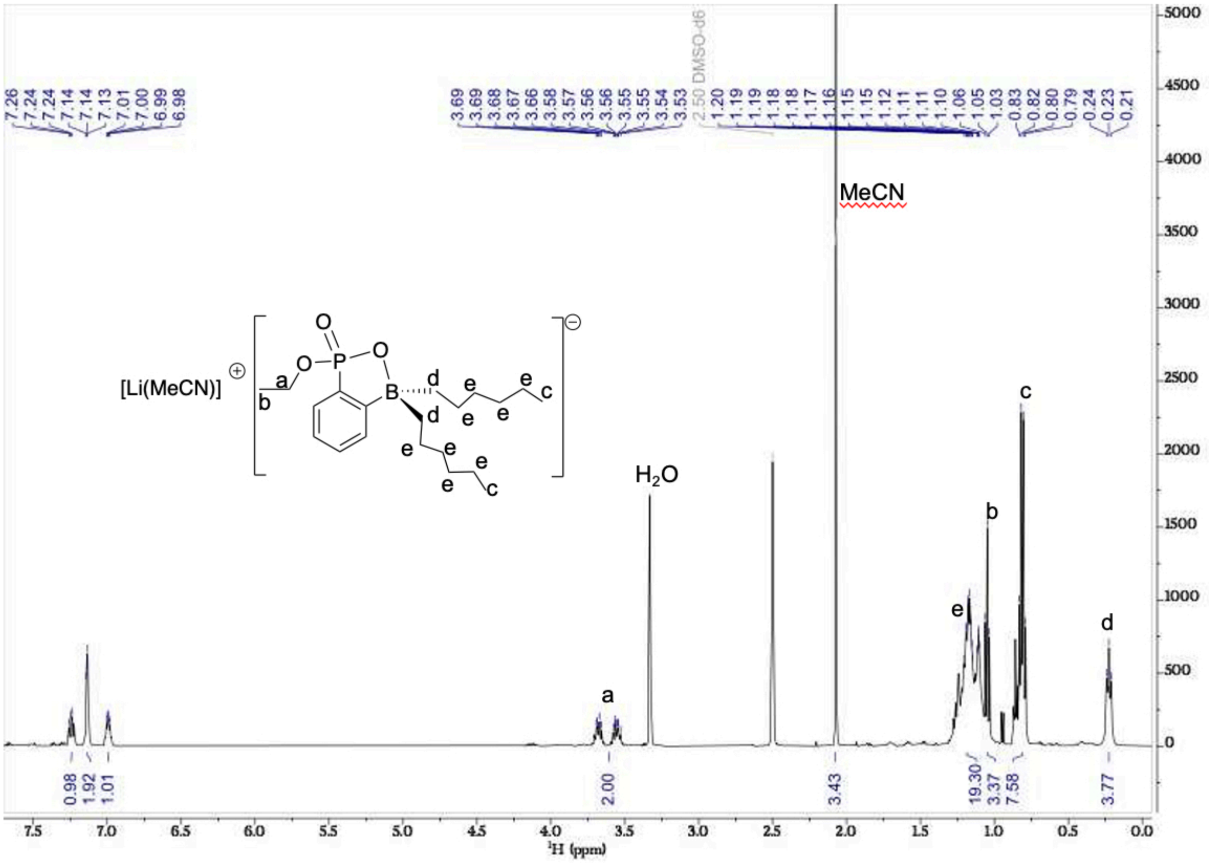

Figure S35. ^{1}H NMR (500 MHz, DMSO- d_6) spectrum of $[Li(MeCN)_2]$[**5**].

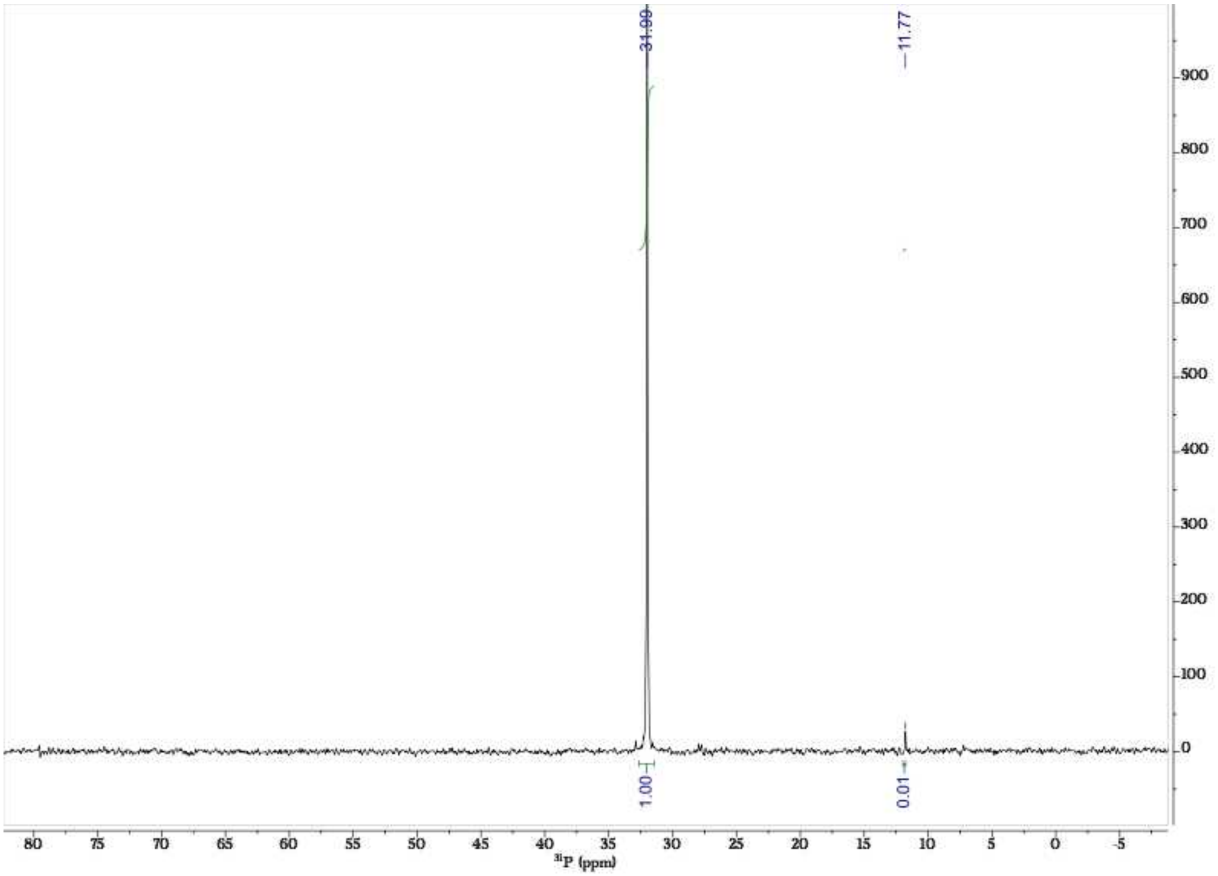

Figure S36. $^{31}P\{^1H\}$ NMR (202 MHz, DMSO-d$_6$) spectrum of [Li(MeCN)$_2$][**5**].

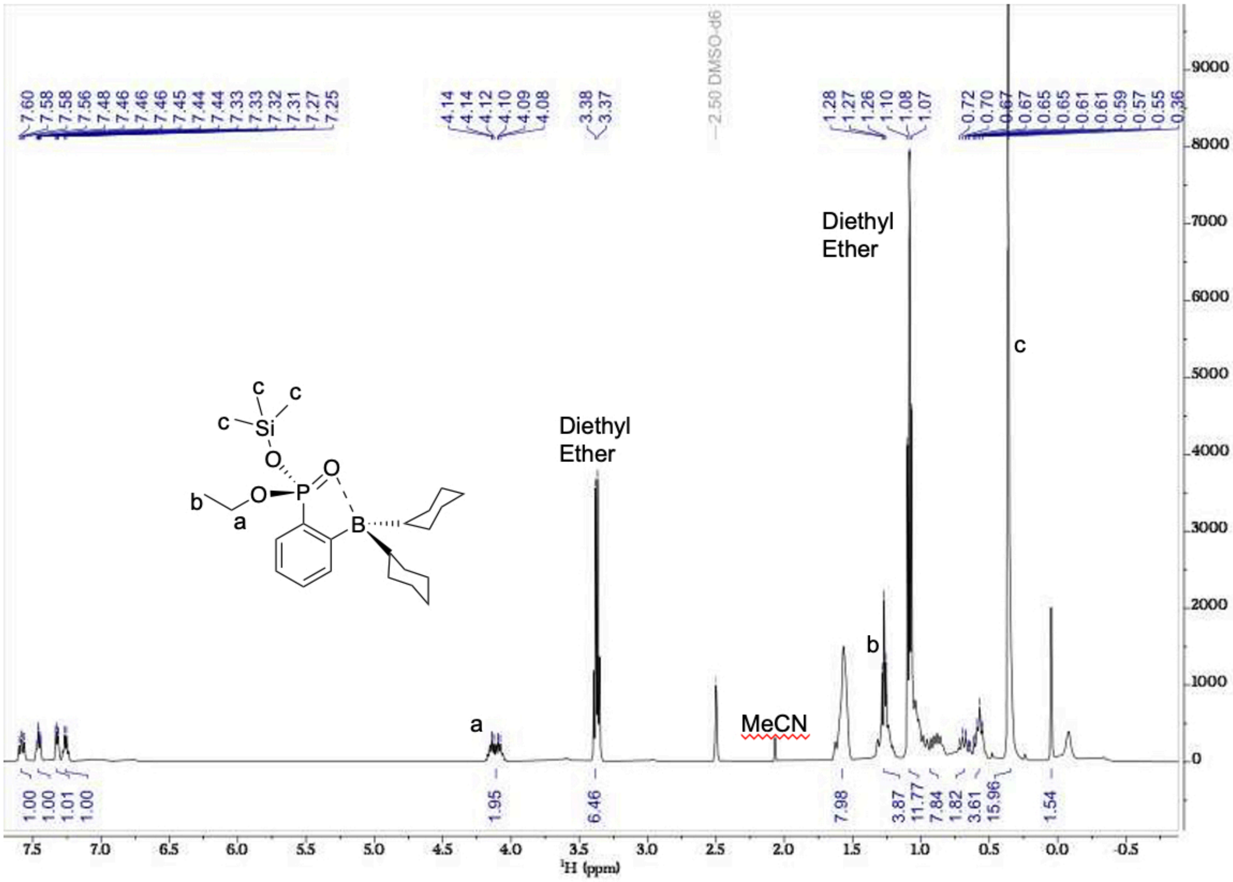

Figure S37. ^{1}H NMR (500 MHz, DMSO-d_6) spectrum of **6**.

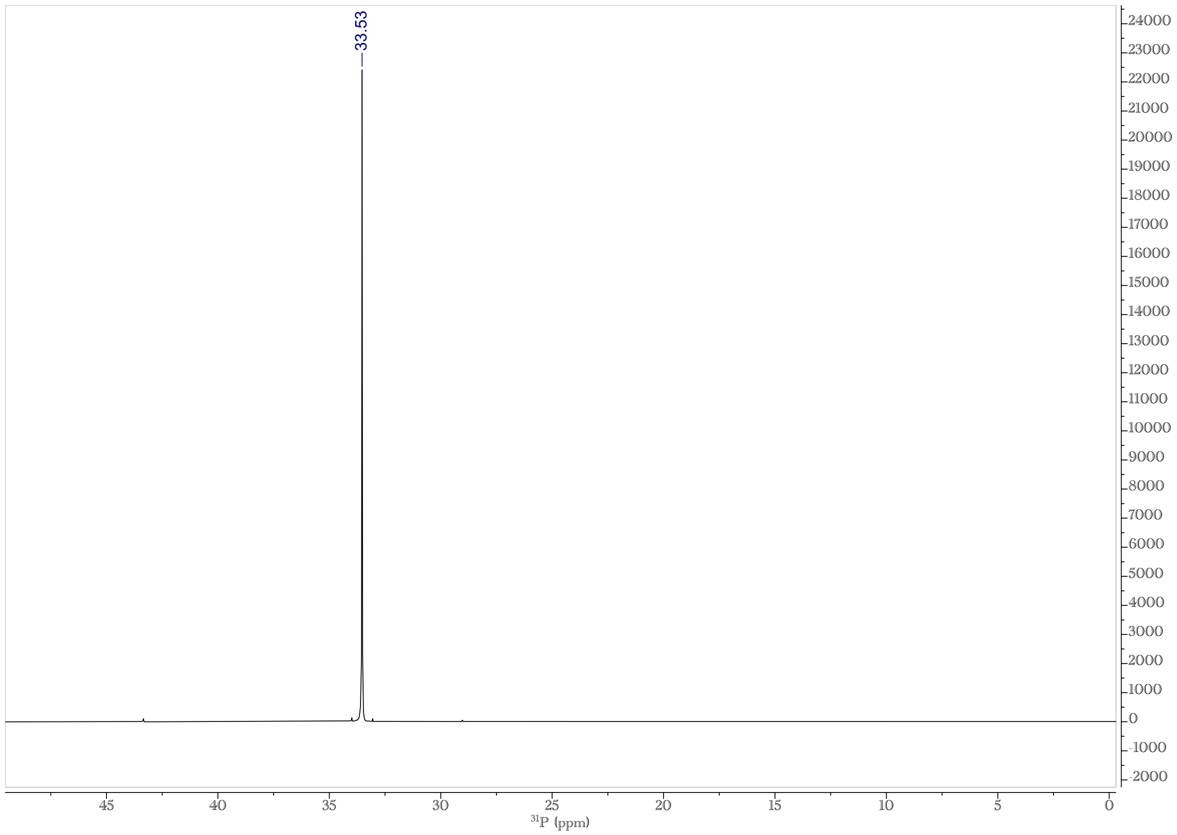

Figure S38. $^{31}P\{^{1}H\}$ NMR (202 MHz, Chloroform-d) spectrum of **6**.

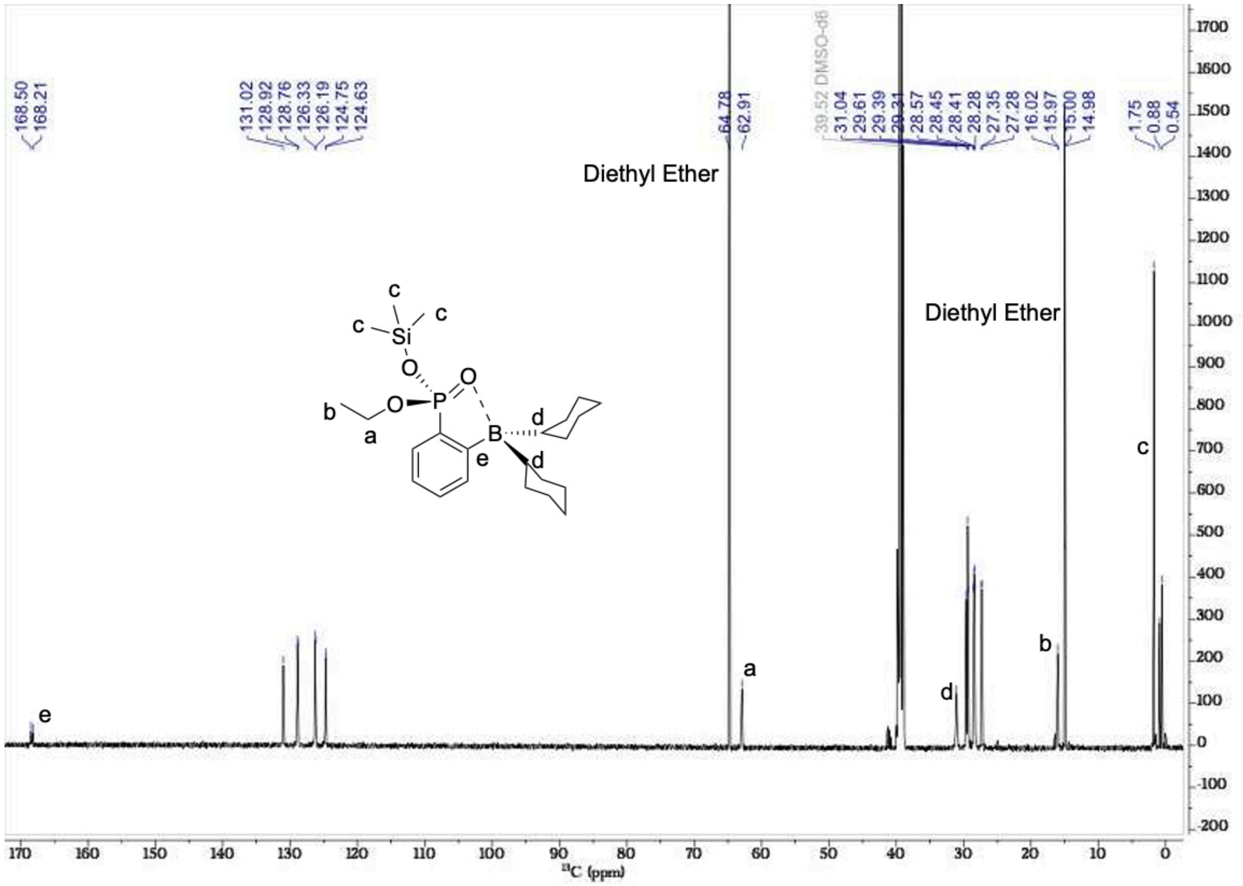

Figure S39. $^{13}C\{^{1}H\}$ NMR (126 MHz, DMSO-d$_6$) spectrum of **6**.

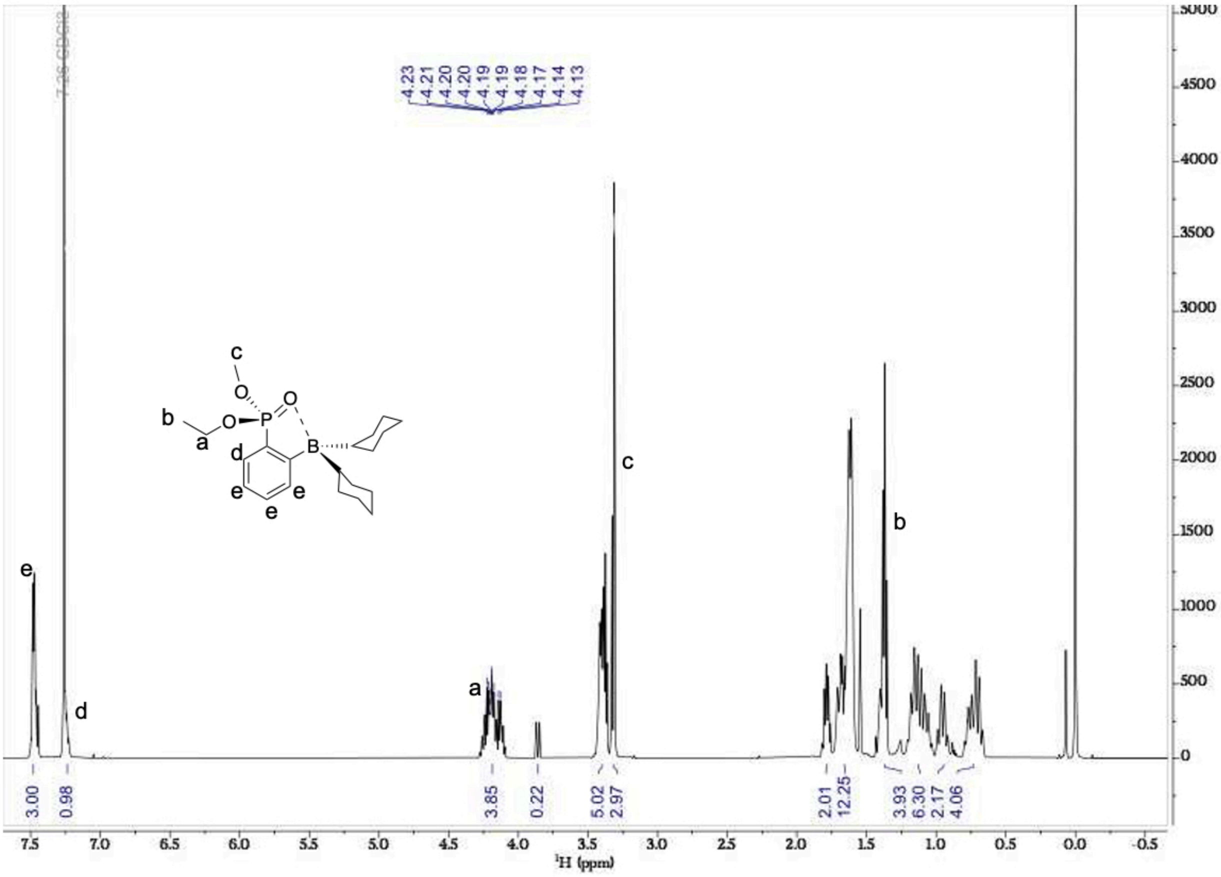

Figure S40. ^{1}H NMR (500 MHz, Chloroform-d) spectrum of **7**.

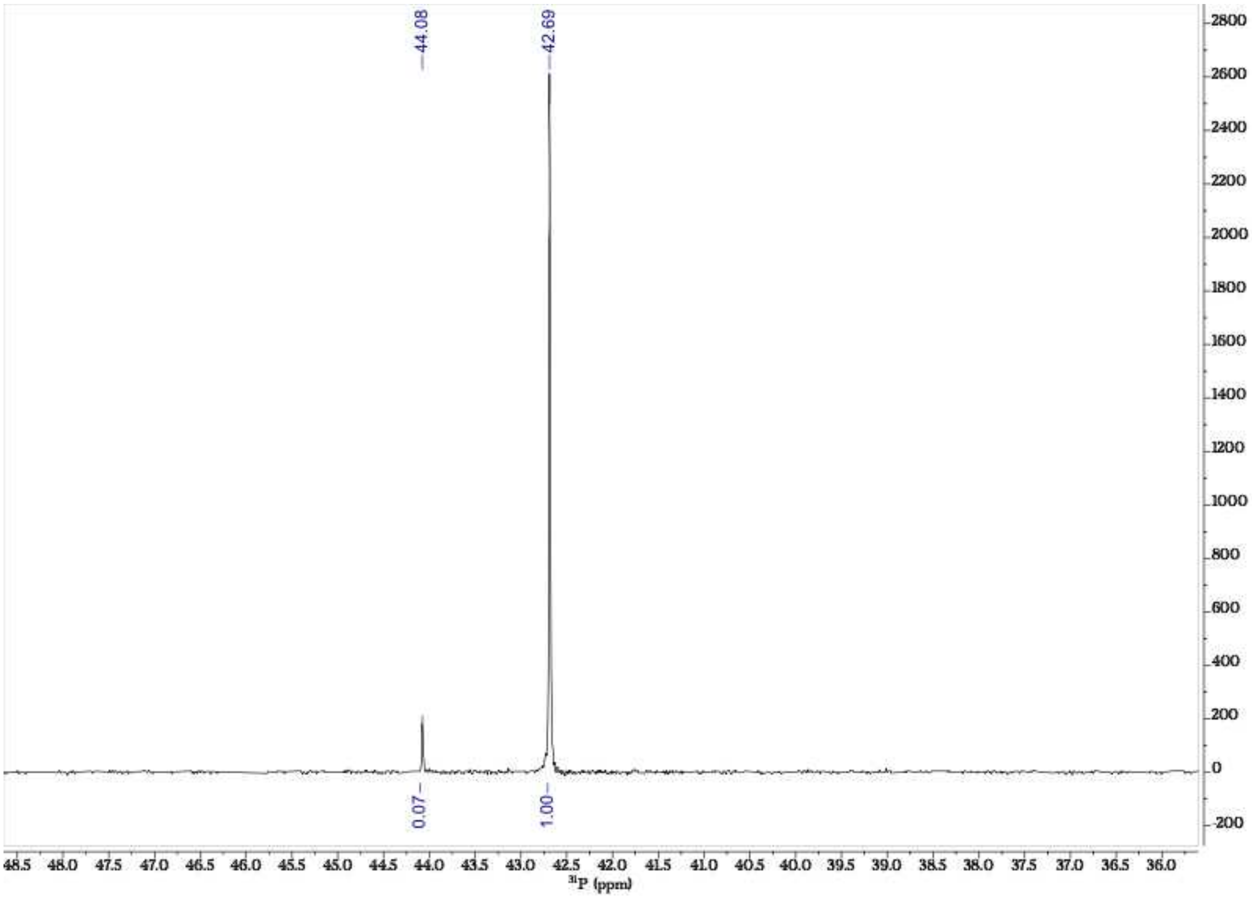

Figure S41. $^{31}P\{^1H\}$ NMR (202 MHz, Chloroform-d) spectrum of **7**.

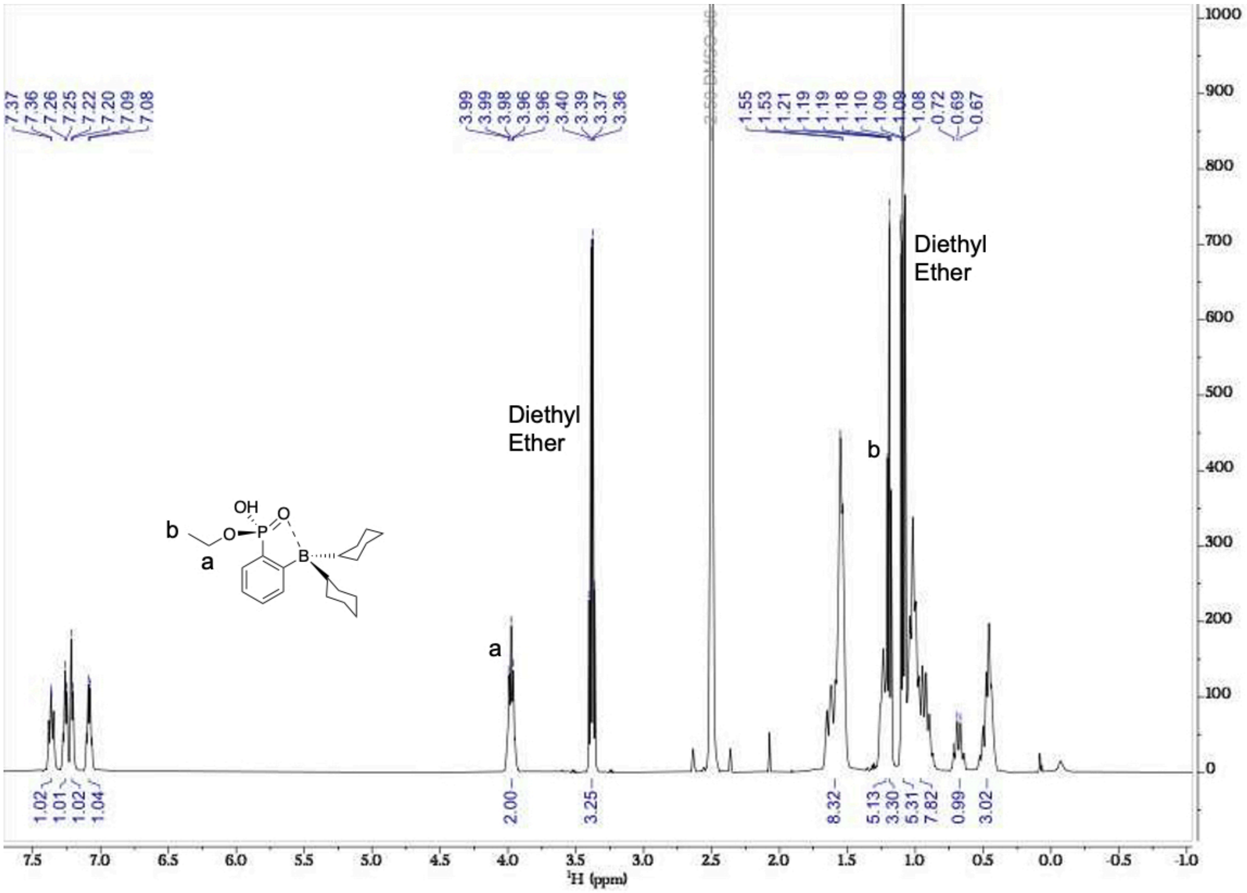

Figure S42. ^{1}H NMR (500 MHz, DMSO-d$_6$) spectrum of **8**.

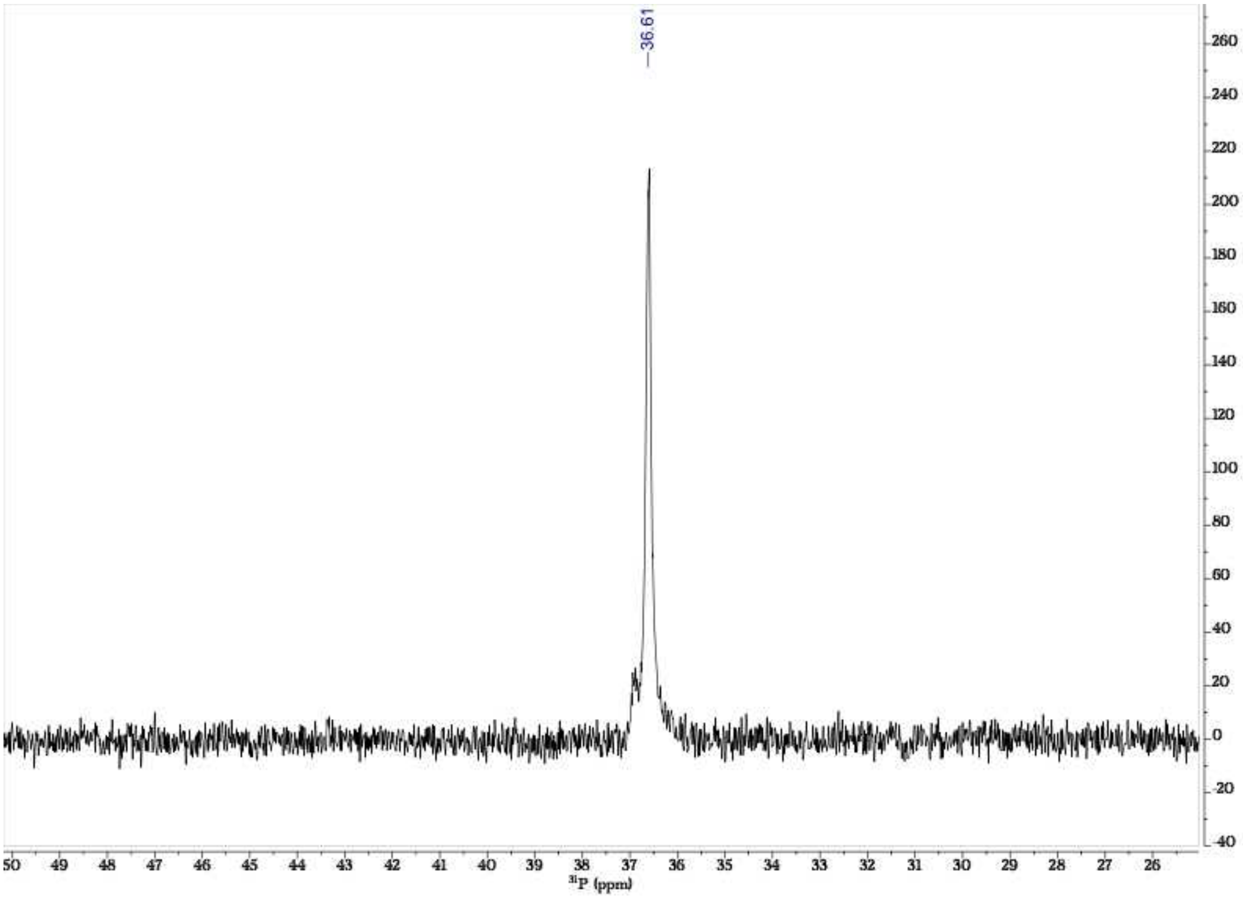

Figure S43. $^{31}P\{^{1}H\}$ NMR (202 MHz, DMSO-d_6) spectrum of **8**.

3. Solubility of [Li(MeCN)$_2$][3] and [Li(EtOAc)$_3$][4]

Into a dry 3 mL vial [Li(MeCN)$_2$][**3**] or [Li(EtOAc)$_3$][**4**] was massed out (10-20 mg). The chosen solvent was then added dropwise via syringe, until the solution appeared homogeneous. The solubility was measured at room temperature (18.5°C).

Table S1. Solubility of [Li(MeCN)$_2$][**3**] in selected solvents.

**Solvent**	**M (mol/L)**
H_2O	0.2
Ethanol	0.4
THF	0.2
DMSO	0.5
MeCN	<0.01
Propylene Carbonate	0.1
DEC	0.3
EMC	0.2
Toluene	0.1
DEC/EMC (50:50 by volume)	0.7

Table S2. Solubility of $[Li(EtOAc)_3]$[**4**] in selected solvents.

**Solvent**	**M (mol/L)**
DEC	0.9
EMC	1.0
Hexanes	1.2
Toluene	1.3
DCM	0.8

4. UV-vis absorption and emission spectra of 2a and [Li(MeCN)$_2$][3]

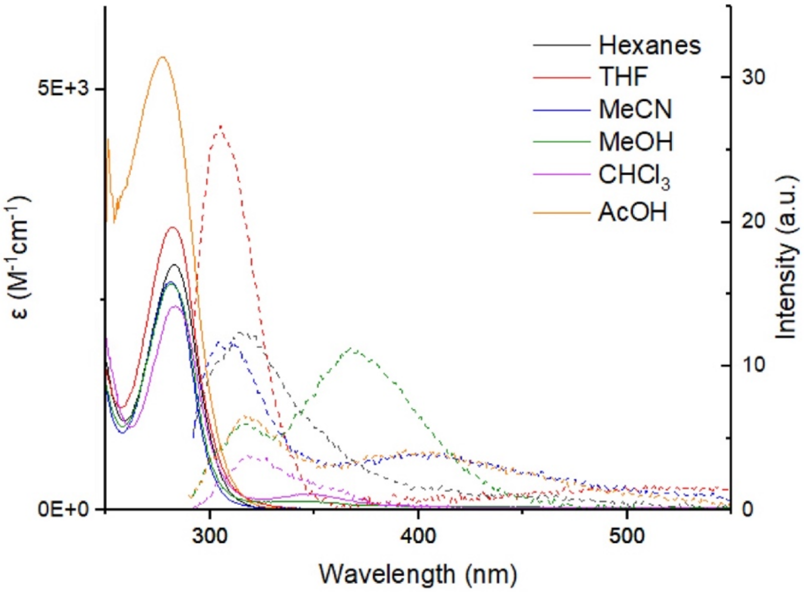

Figure S44. UV-vis absorption (solid) and fluorescence (dashed) of **2a** in THF (black), Hexanes (red), MeCN (blue), MeOH (green), $CHCl_3$ (purple), and AcOH (orange) (concentration 5•10^{-5}M). Excitation wavelength for fluorescence measurements was the absorption maxima (λ = 282 nm).

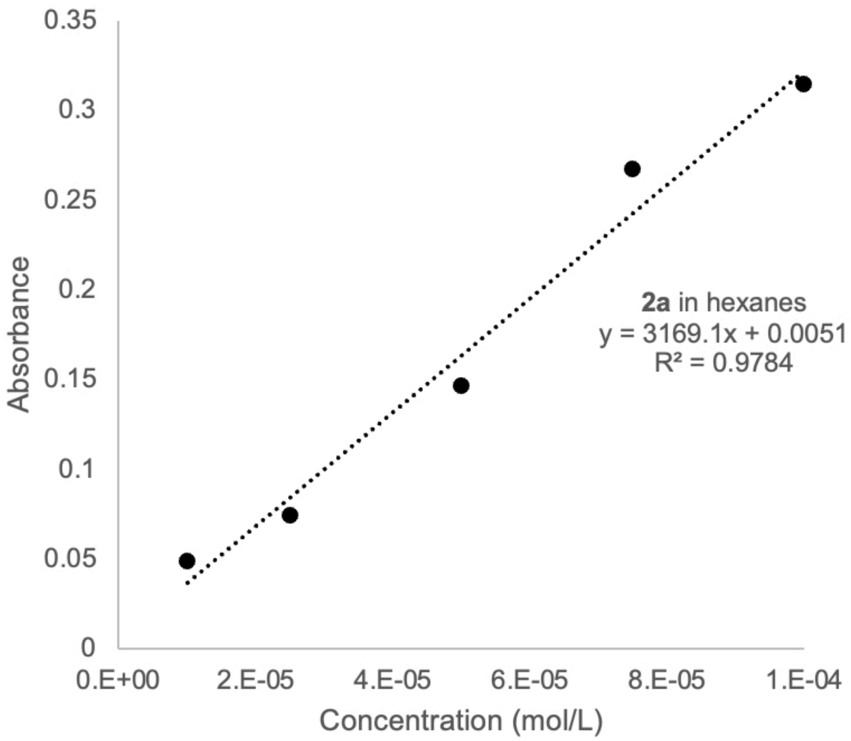

Figure S45. Beer's law plot of **2a** in hexanes (λ = 282 nm and ε = 2921)

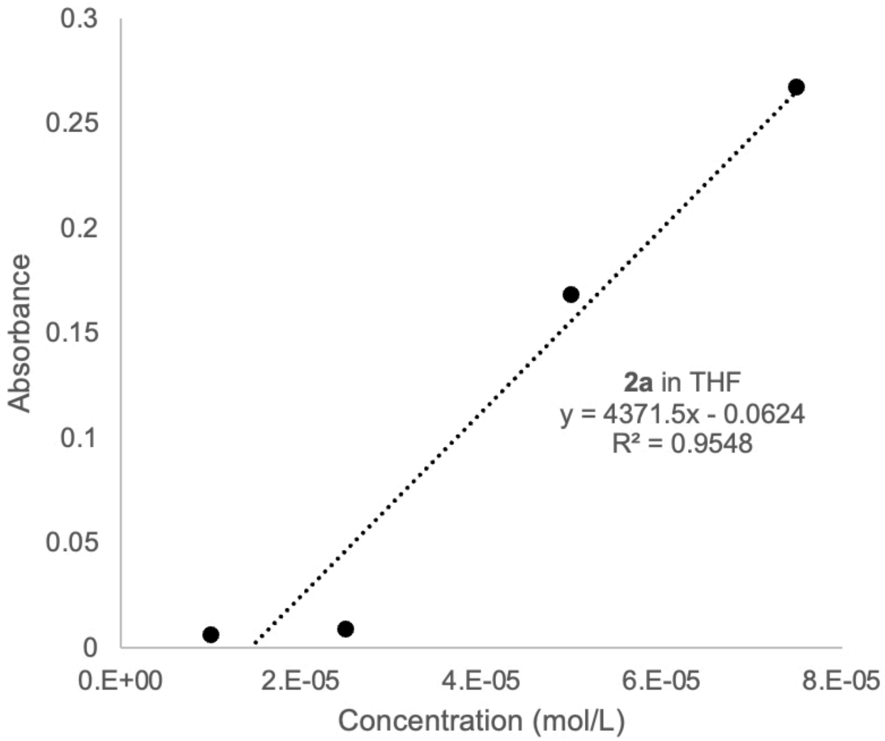

Figure S46. Beer's law plot of **2a** in THF (λ = 282 nm and ε = 3372)

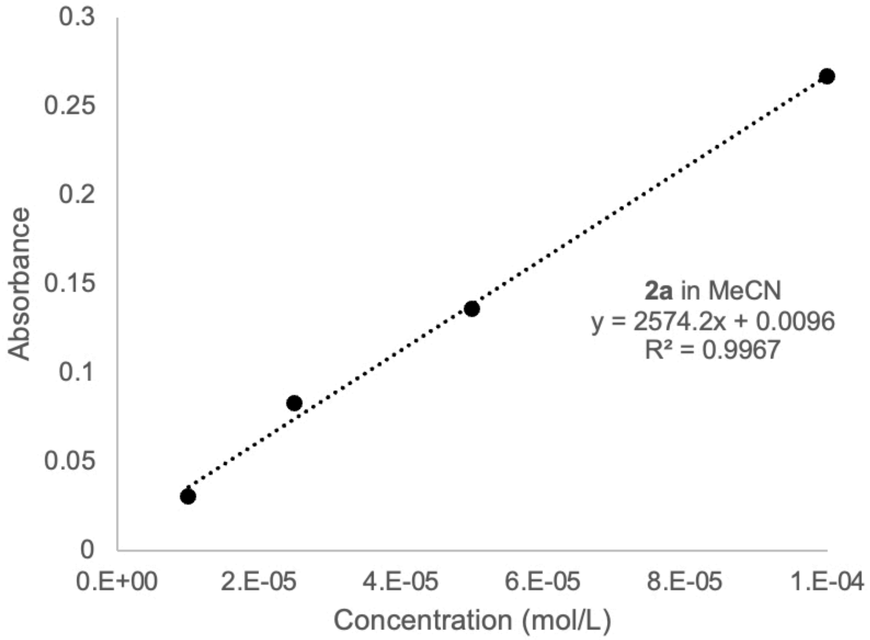

Figure S47. Beer's law plot of **2a** in MeCN (λ = 282 nm and ε = 2668)

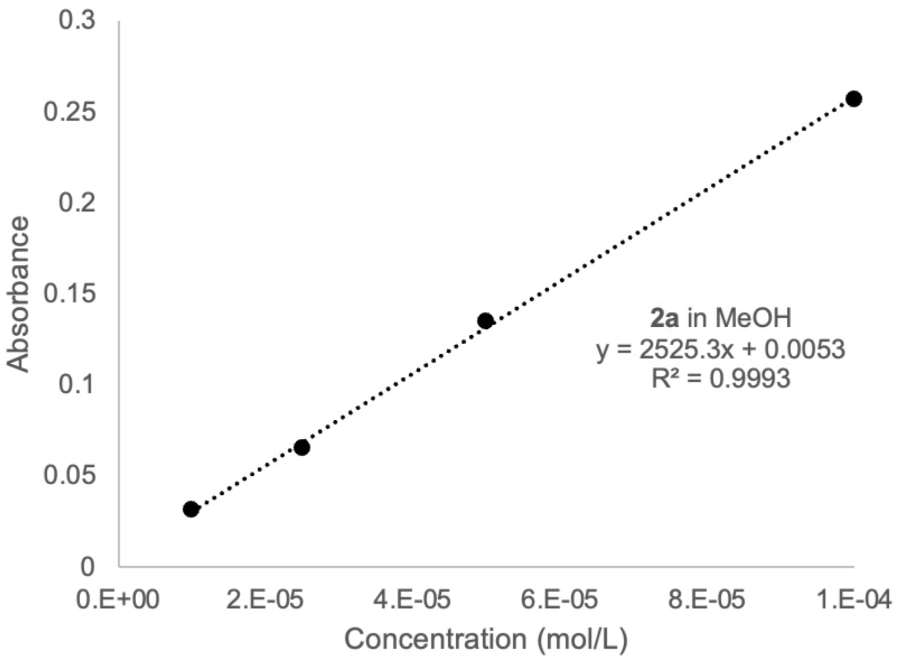

Figure S48. Beer's law plot of **2a** in MeOH (λ = 282 nm and ε = 2574)

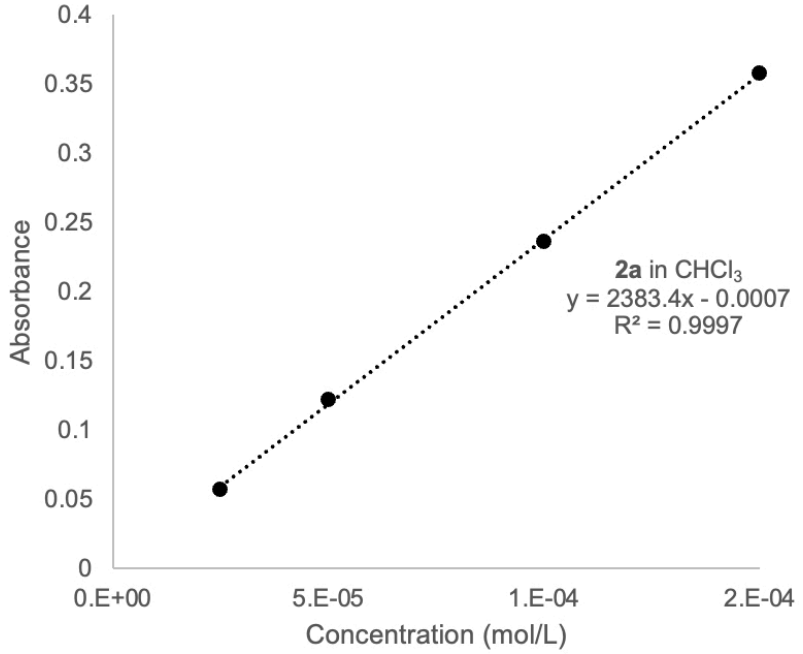

Figure S49. Beer's law plot of **2a** in $CHCl_3$ (λ = 282 nm and ε = 2381)

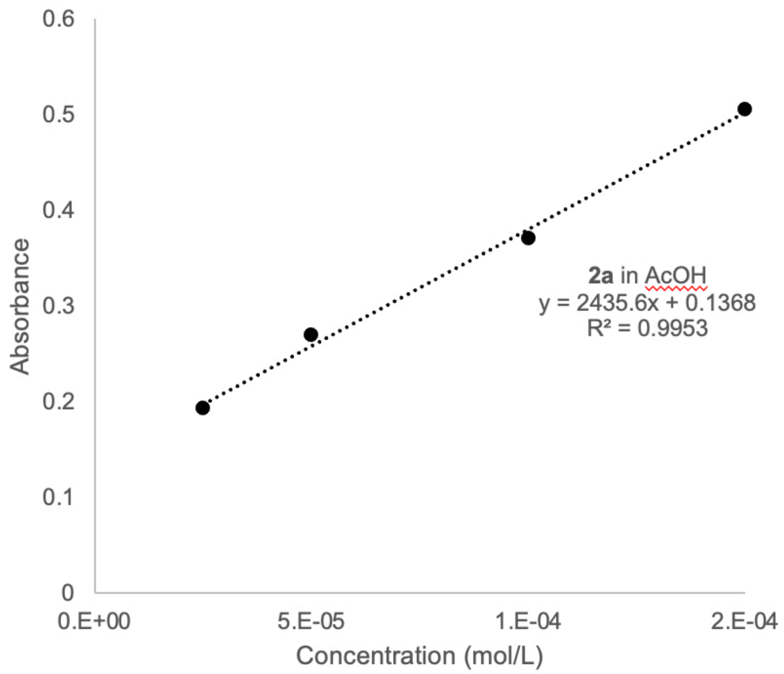

Figure S50. Beer's law plot of **2a** in AcOH (λ = 282 nm and ε = 3372)

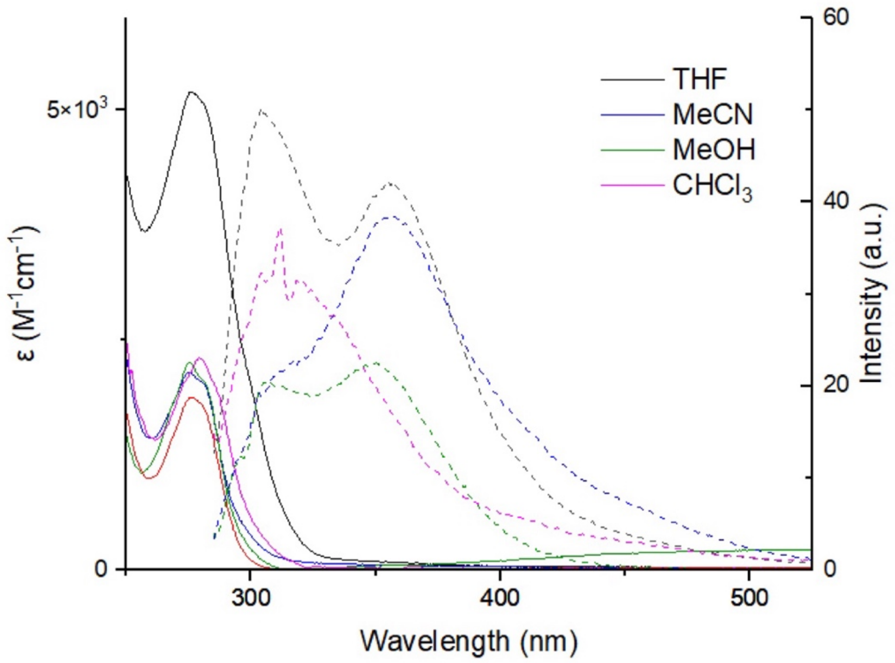

Figure S51. UV-vis absorption (solid) and fluorescence (dashed) of [Li(MeCN)$_2$][**3**] in THF (black), MeCN (blue), MeOH (green), and $CHCl_3$ (purple) (concentration 5•10^{-5}M). Excitation wavelength for fluorescence measurements was the absorption maxima (λ = 275 nm).

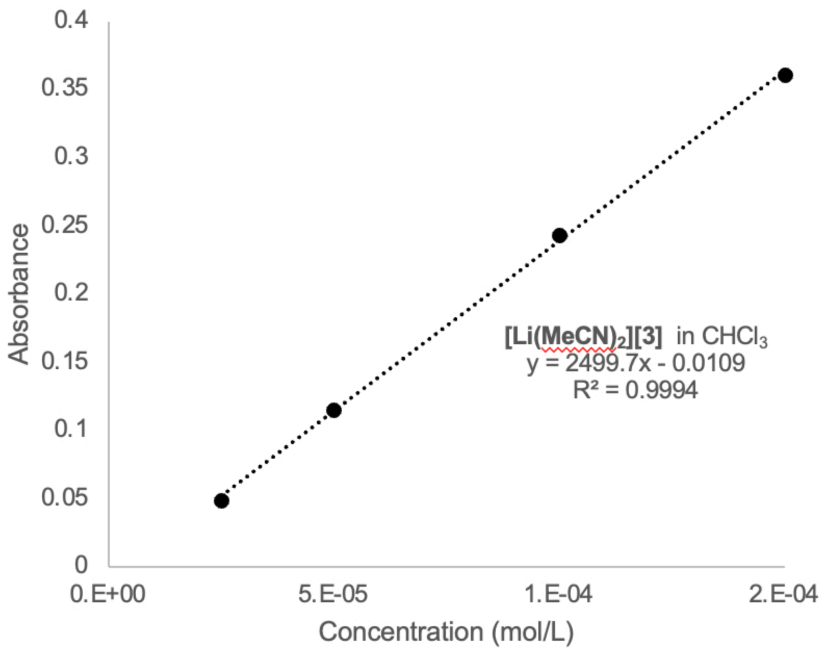

Figure S52. Beer's law plot of [Li(MeCN)$_2$][**3**] in $CHCl_3$ (λ = 275 nm and ε = 2408).

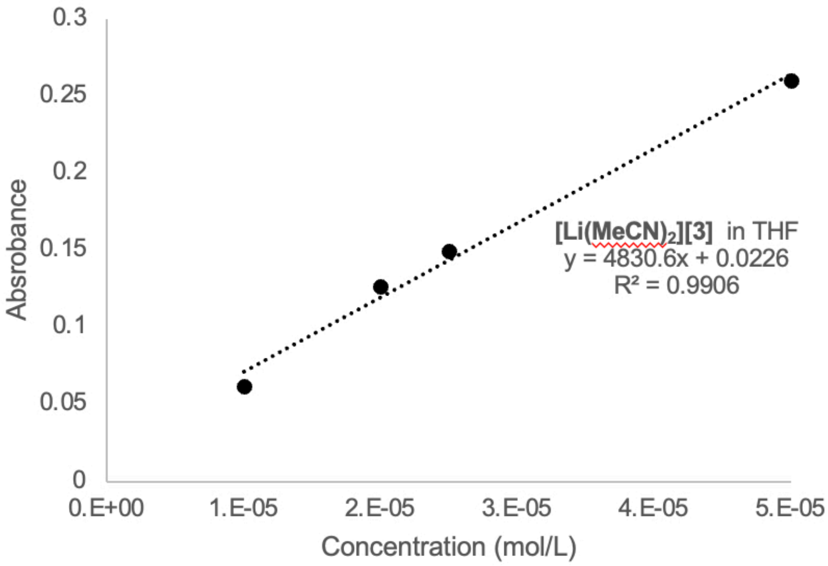

Figure S53. Beer's law plot of [Li(MeCN)$_2$][**3**] in THF (λ = 275 nm and ε = 8534).

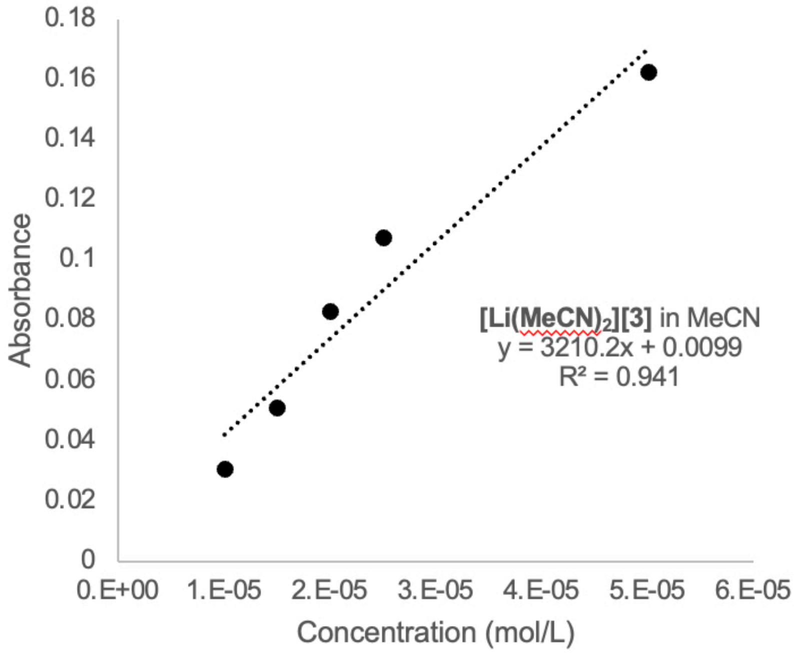

Figure S54. Beer's law plot of [Li(MeCN)$_2$][**3**] in MeCN (λ = 275 nm and ε = 6502).

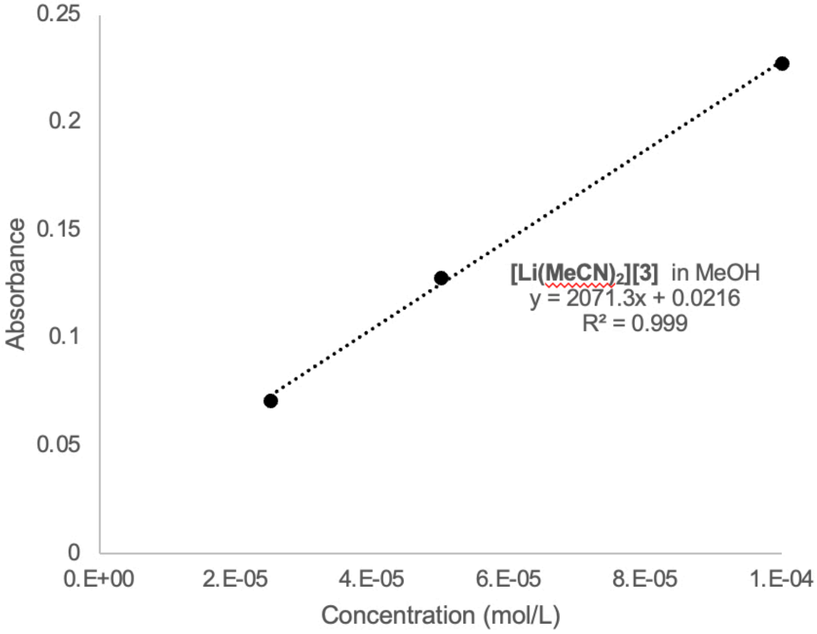

Figure S55. Beer's law plot of [Li(MeCN)$_2$][**3**] in MeOH (λ = 275 nm and ε = 2277).

5. Thermal gravimetric analysis of [Li(MeCN)$_2$][3]

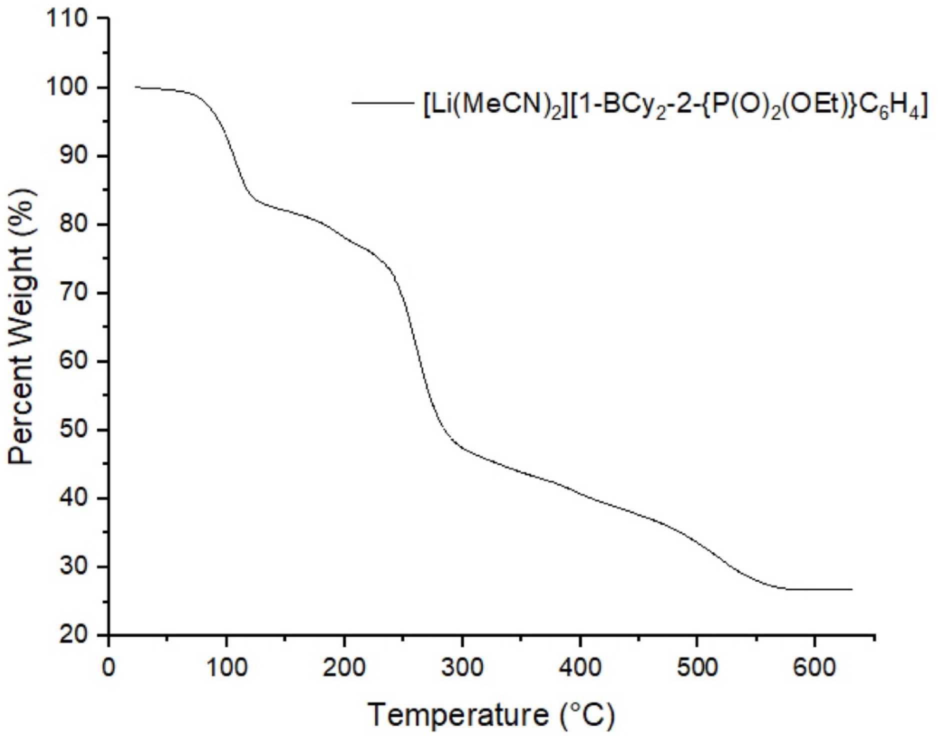

Figure S56. TGA (ramp rate of 20°C per minute to 700°C, atmosphere N_2) of [Li(MeCN)$_2$][**3**].

6. Thermalysis of [Li(MeCN)$_2$][3] in solution

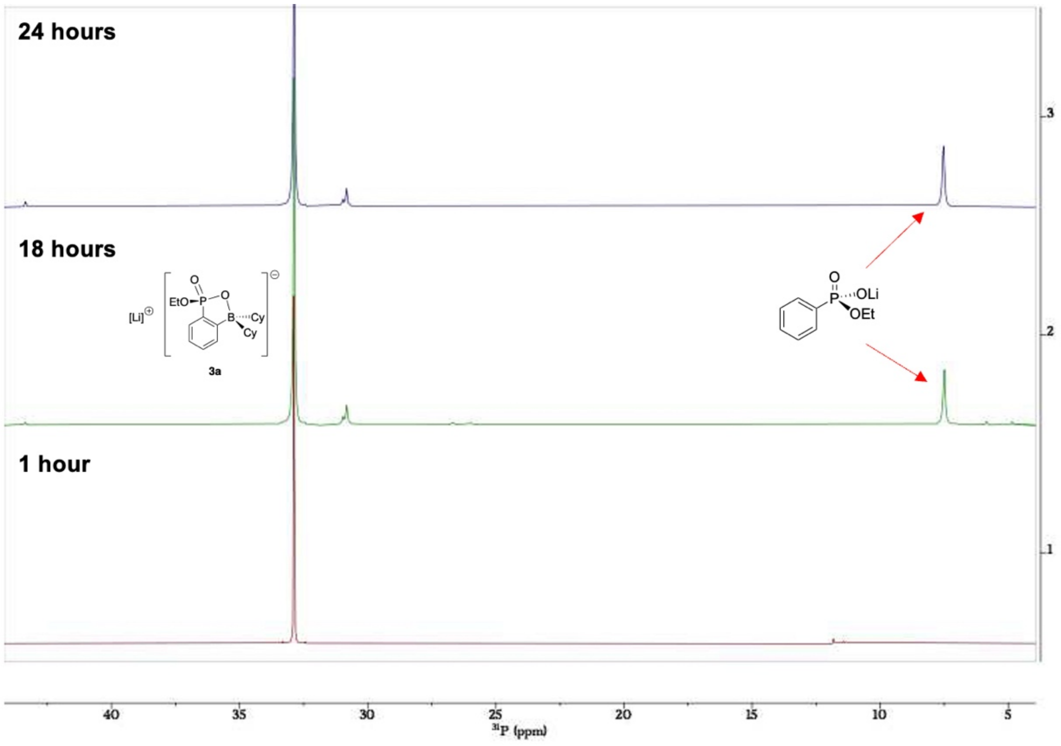

Figure S57. Monitoring thermal degradation of [Li(MeCN)$_2$]**3** over 24 hours by ^{31}P{^{1}H} NMR spectroscopy.

7. Crystal data for compounds 2a and [Li(MeCN)$_2$][3].

Crystals of compound **2a**, suitable for X-ray diffraction, were grown from a concentrated solution of toluene at -20 °C.

Data for **2a** and [Li(MeCN)$_2$][**3**] were collected from a shock-cooled single crystal at 100.15 K on a Bruker APEX-II Ultra Kappa Four-Circle Diffractometer with a Micro Focus Rotating Anode using a Double Bounce Multilayer Mirrors as monochromator and a CCD Area Detector detector. The diffractometer used MoK_α radiation (λ = 0.71073 Å). All data were integrated with SAINT v8.40B and a multi-scan absorption correction using SADABS 2016/2 was applied.[1,2] The structure was solved by direct methods with SHELXS and refined by full-matrix least-squares methods against F^2 using XL.[3,4] All non-hydrogen atoms were refined with anisotropic displacement parameters. All hydrogen atoms were refined isotropic on calculated positions using a riding model with their U_{iso} values constrained to 1.5 times the U_{eq} of their pivot atoms for terminal sp^3 carbon atoms and 1.2 times for all other carbon atoms. During the refinement the presence of residual electron density was noted in the vicinity of C2. This was successfully modeled as a bromine atom at 2% occupancy, which presumably is the result of co-crystallization of **2a** with 2% impurity of **2a*** (below) in this particular crystal. Under certain conditions it is known that deprotonation (directed metallation) can be faster that metal-halogen exchange (see for example Anna Cederbalk, Morten Lysén, Jan Kehler, Jesper L. Kristensen, "*Ortho* lithiation-in situ borylation of substituted morpholine benzamides" *Tetrahedron*, **2017**, *73*, 1576-1582. https://doi.org/10.1016/j.tet.2017.02.002).

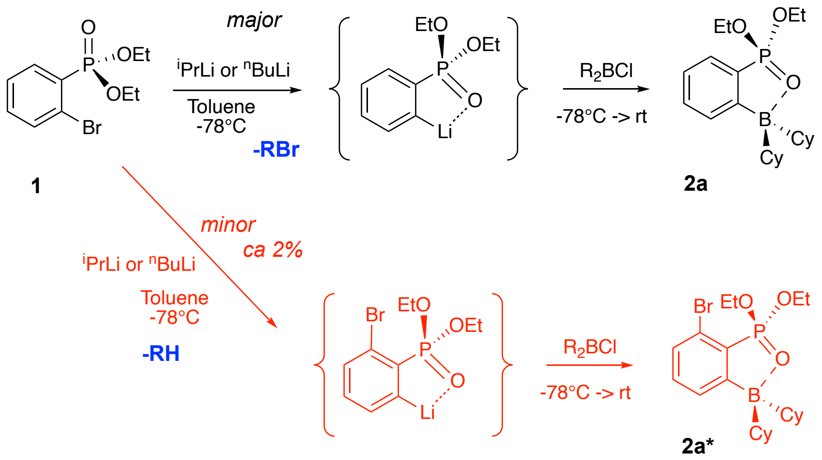

Table S3. Crystal data and structure refinement for **2a**

CCDC number	
Empirical formula	C22 H36 B Br0.02 O3 P
Formula weight	392.08
Temperature	100.15 K
Wavelength	0.71073 Å
Crystal system	Monoclinic
Space group	P 1 21/c 1
Unit cell dimensions	a = 10.5034(8) Å a= 90°.
	b = 12.4129(9) Å b= 95.025(2)°.
	c = 17.0752(12) Å g = 90°.
Volume	2217.7(3) Å3
Z	4
Density (calculated)	1.174 Mg/m3
Absorption coefficient	0.178 mm-1
F(000)	851
Crystal size	0.33 x 0.29 x 0.28 mm3
Theta range for data collection	1.946 to 26.372°.
Index ranges	-12<=h<=13, -12<=k<=15, -21<=l<=21
Reflections collected	14306
Independent reflections	4544 [R(int) = 0.0502]
Completeness to theta = 25.242°	100.0 %
Absorption correction	Semi-empirical from equivalents
Max. and min. transmission	0.5628 and 0.5181
Refinement method	Full-matrix least-squares on F2
Data / restraints / parameters	4544 / 0 / 256
Goodness-of-fit on F2	1.018
Final R indices [I>2sigma(I)]	R1 = 0.0427, wR2 = 0.0959
R indices (all data)	R1 = 0.0603, wR2 = 0.1057
Extinction coefficient	n/a
Largest diff. peak and hole	0.449 and -0.311 e.Å-3

Table S4. Bond lengths of **2a**

**Atom–Atom**	**Length [Å]**
P(1)-O(1)	1.5132(12)
P(1)-O(2)	1.5606(13)
P(1)-O(3)	1.5609(12)
P(1)-C(1)	1.7549(16)
O(1)-B(1)	1.666(2)
O(2)-C(9)	1.464(2)
O(3)-C(7)	1.4619(19)
C(1)-C(2)	1.403(2)
C(1)-C(6)	1.404(2)
C(2)-H(2)	0.9500
C(2)-C(3)	1.380(2)
C(4)-H(4)	0.9500
C(4)-C(5)	1.385(2)
C(4)-C(3)	1.392(2)
C(5)-H(5)	0.9500
C(5)-C(6)	1.405(2)
C(6)-B(1)	1.629(2)
C(7)-H(7A)	0.9900
C(7)-H(7B)	0.9900
C(7)-C(8)	1.497(3)
C(8)-H(8A)	0.9800
C(8)-H(8B)	0.9800
C(8)-H(8C)	0.9800
C(9)-H(9A)	0.9900
C(9)-H(9B)	0.9900
C(9)-C(10)	1.498(3)
C(10)-H(10A)	0.9800
C(10)-H(10B)	0.9800
C(10)-H(10C)	0.9800
C(11)-H(11)	1.0000
C(11)-C(12)	1.538(2)
C(11)-C(16)	1.529(2)
C(11)-B(1)	1.621(2)
C(12)-H(12A)	0.9900
C(12)-H(12B)	0.9900
C(12)-C(13)	1.534(3)
C(13)-H(13A)	0.9900
C(13)-H(13B)	0.9900
C(13)-C(14)	1.525(3)
C(14)-H(14A)	0.9900
C(14)-H(14B)	0.9900
C(14)-C(15)	1.524(2)
C(15)-H(15A)	0.9900
C(15)-H(15B)	0.9900
C(15)-C(16)	1.532(2)
C(16)-H(16A)	0.9900

C(16)-H(16B)	0.9900
C(17)-H(17)	1.0000
C(17)-C(18)	1.539(2)
C(17)-C(22)	1.533(2)
C(17)-B(1)	1.625(3)
C(18)-H(18A)	0.9900
C(18)-H(18B)	0.9900
C(18)-C(19)	1.523(3)
C(19)-H(19A)	0.9900
C(19)-H(19B)	0.9900
C(19)-C(20)	1.514(3)
C(20)-H(20A)	0.9900
C(20)-H(20B)	0.9900
C(20)-C(21)	1.522(3)
C(21)-H(21A)	0.9900
C(21)-H(21B)	0.9900
C(21)-C(22)	1.529(2)
C(22)-H(22A)	0.9900
C(22)-H(22B)	0.9900
C(3)-H(3)	0.9500

Table S5. Bond angles of **2a**

**Atom–Atom–Atom**	**Angle [°]**
O(1)-P(1)-O(2)	113.11(7)
O(1)-P(1)-O(3)	114.97(7)
O(1)-P(1)-C(1)	103.12(7)
O(2)-P(1)-O(3)	101.46(7)
O(2)-P(1)-C(1)	116.88(7)
O(3)-P(1)-C(1)	107.70(7)
P(1)-O(1)-B(1)	112.67(9)
C(9)-O(2)-P(1)	123.06(11)
C(7)-O(3)-P(1)	121.23(10)
C(2)-C(1)-P(1)	128.81(14)
C(2)-C(1)-C(6)	123.87(15)
C(6)-C(1)-P(1)	107.24(12)
C(1)-C(2)-H(2)	120.8
C(3)-C(2)-C(1)	118.32(16)
C(3)-C(2)-H(2)	120.8
C(5)-C(4)-H(4)	119.2
C(5)-C(4)-C(3)	121.59(15)
C(3)-C(4)-H(4)	119.2
C(4)-C(5)-H(5)	119.5
C(4)-C(5)-C(6)	121.10(16)
C(6)-C(5)-H(5)	119.5
C(1)-C(6)-C(5)	115.66(15)
C(1)-C(6)-B(1)	116.04(14)
C(5)-C(6)-B(1)	128.27(15)
O(3)-C(7)-H(7A)	110.4
O(3)-C(7)-H(7B)	110.4
O(3)-C(7)-C(8)	106.84(14)
H(7A)-C(7)-H(7B)	108.6
C(8)-C(7)-H(7A)	110.4
C(8)-C(7)-H(7B)	110.4
C(7)-C(8)-H(8A)	109.5
C(7)-C(8)-H(8B)	109.5
C(7)-C(8)-H(8C)	109.5
H(8A)-C(8)-H(8B)	109.5
H(8A)-C(8)-H(8C)	109.5
H(8B)-C(8)-H(8C)	109.5
O(2)-C(9)-H(9A)	110.3
O(2)-C(9)-H(9B)	110.3
O(2)-C(9)-C(10)	107.10(14)
H(9A)-C(9)-H(9B)	108.5
C(10)-C(9)-H(9A)	110.3
C(10)-C(9)-H(9B)	110.3
C(9)-C(10)-H(10A)	109.5
C(9)-C(10)-H(10B)	109.5
C(9)-C(10)-H(10C)	109.5
H(10A)-C(10)-H(10B)	109.5

H(10A)-C(10)-H(10C)	109.5
H(10B)-C(10)-H(10C)	109.5
C(12)-C(11)-H(11)	106.0
C(12)-C(11)-B(1)	113.74(13)
C(16)-C(11)-H(11)	106.0
C(16)-C(11)-C(12)	109.13(14)
C(16)-C(11)-B(1)	115.17(14)
B(1)-C(11)-H(11)	106.0
C(11)-C(12)-H(12A)	109.1
C(11)-C(12)-H(12B)	109.1
H(12A)-C(12)-H(12B)	107.8
C(13)-C(12)-C(11)	112.60(15)
C(13)-C(12)-H(12A)	109.1
C(13)-C(12)-H(12B)	109.1
C(12)-C(13)-H(13A)	109.3
C(12)-C(13)-H(13B)	109.3
H(13A)-C(13)-H(13B)	108.0
C(14)-C(13)-C(12)	111.47(17)
C(14)-C(13)-H(13A)	109.3
C(14)-C(13)-H(13B)	109.3
C(13)-C(14)-H(14A)	109.5
C(13)-C(14)-H(14B)	109.5
H(14A)-C(14)-H(14B)	108.1
C(15)-C(14)-C(13)	110.54(16)
C(15)-C(14)-H(14A)	109.5
C(15)-C(14)-H(14B)	109.5
C(14)-C(15)-H(15A)	109.5
C(14)-C(15)-H(15B)	109.5
C(14)-C(15)-C(16)	110.94(14)
H(15A)-C(15)-H(15B)	108.0
C(16)-C(15)-H(15A)	109.5
C(16)-C(15)-H(15B)	109.5
C(11)-C(16)-C(15)	112.82(14)
C(11)-C(16)-H(16A)	109.0
C(11)-C(16)-H(16B)	109.0
C(15)-C(16)-H(16A)	109.0
C(15)-C(16)-H(16B)	109.0
H(16A)-C(16)-H(16B)	107.8
C(18)-C(17)-H(17)	107.1
C(18)-C(17)-B(1)	110.71(13)
C(22)-C(17)-H(17)	107.1
C(22)-C(17)-C(18)	109.81(14)
C(22)-C(17)-B(1)	114.76(14)
B(1)-C(17)-H(17)	107.1
C(17)-C(18)-H(18A)	108.7
C(17)-C(18)-H(18B)	108.7
H(18A)-C(18)-H(18B)	107.6
C(19)-C(18)-C(17)	114.14(15)

C(19)-C(18)-H(18A)	108.7
C(19)-C(18)-H(18B)	108.7
C(18)-C(19)-H(19A)	109.4
C(18)-C(19)-H(19B)	109.4
H(19A)-C(19)-H(19B)	108.0
C(20)-C(19)-C(18)	111.36(16)
C(20)-C(19)-H(19A)	109.4
C(20)-C(19)-H(19B)	109.4
C(19)-C(20)-H(20A)	109.5
C(19)-C(20)-H(20B)	109.5
C(19)-C(20)-C(21)	110.66(15)
H(20A)-C(20)-H(20B)	108.1
C(21)-C(20)-H(20A)	109.5
C(21)-C(20)-H(20B)	109.5
C(20)-C(21)-H(21A)	109.3
C(20)-C(21)-H(21B)	109.3
C(20)-C(21)-C(22)	111.47(15)
H(21A)-C(21)-H(21B)	108.0
C(22)-C(21)-H(21A)	109.3
C(22)-C(21)-H(21B)	109.3
C(17)-C(22)-H(22A)	108.8
C(17)-C(22)-H(22B)	108.8
C(21)-C(22)-C(17)	113.83(15)
C(21)-C(22)-H(22A)	108.8
C(21)-C(22)-H(22B)	108.8
H(22A)-C(22)-H(22B)	107.7
C(6)-B(1)-O(1)	99.94(12)
C(11)-B(1)-O(1)	107.11(12)
C(11)-B(1)-C(6)	113.87(13)
C(11)-B(1)-C(17)	114.98(14)
C(17)-B(1)-O(1)	106.35(12)
C(17)-B(1)-C(6)	112.95(13)
C(2)-C(3)-C(4)	119.45(15)
C(2)-C(3)-H(3)	120.3
C(4)-C(3)-H(3)	120.3

X-ray quality crystals of **3a** were grown from a warm solution of acetonitrile and cooling to -20°C overnight.

Table S6. Crystal data and structure refinement for [Li(MeCN)$_2$][**3**].

CCDC number	
Empirical formula	$C_{48}H_{74}B_2Li_2N_4O_6P_2$
Formula weight	900.55
Temperature [K]	100.15
Crystal system	triclinic
Space group (number)	$P\bar{1}$ (2)
a [Å]	9.5239(5)
b [Å]	10.8118(6)
c [Å]	14.3061(6)
α [°]	71.418(2)
β [°]	82.662(2)
γ [°]	70.581(2)
Volume [Å^3]	1316.49(12)
Z	1
ρ_{calc} [gcm^{-3}]	1.136
μ [mm^{-1}]	1.119
F(000)	484
Crystal size [mm^3]	0.12×0.3×0.4
Crystal colour	colourless
Crystal shape	block
Radiation	CuK_α (λ=1.54178 Å)
2θ range [°]	6.52 to 136.59 (0.83 Å)
Index ranges	$-11 \leq h \leq 11$
$-12 \leq k \leq 13$	
$-17 \leq l \leq 17$	
Reflections collected	30644
Independent reflections	4733
R_{int} = 0.0363	
R_{sigma} = 0.0185	
Completeness to	
θ = 67.500°	98.4 %
Data / Restraints / Parameters	4733 / 0 / 300
Absorption correction T_{min}/T_{max} (method)	0.5778 / 0.6617
(multi-scan)	
Goodness-of-fit on F^2	1.110
Final R indexes	
[$I \geq 2\sigma(I)$]	R_1 = 0.0398
wR_2 = 0.1071	
Final R indexes	
[all data]	R_1 = 0.0413
wR_2 = 0.1083	
Largest peak/hole [eÅ^{-3}]	0.47/−0.23

Table S7. Atomic coordinates and U_{eq} [Å^2] of [Li(MeCN)$_2$][**3**].

**Atom**	***x***	***y***	***z***	U_{eq}
P1	0.31909(4)	0.67342(4)	0.61229(3)	0.02109(12)
O2	0.26413(11)	0.58653(10)	0.70694(7)	0.0208(2)
O1	0.40677(13)	0.59975(12)	0.54038(8)	0.0274(3)
O3	0.17299(13)	0.78661(13)	0.55979(8)	0.0330(3)
N2	0.66047(19)	0.71578(16)	0.38566(11)	0.0367(4)
N1	0.78162(17)	0.51928(17)	0.58408(11)	0.0369(4)
C5	0.50756(17)	0.73088(15)	0.81661(11)	0.0223(3)
H5	0.512353	0.694384	0.886367	0.027
C9	0.66662(17)	0.23228(16)	0.83432(12)	0.0254(3)
H9A	0.747421	0.207383	0.786382	0.031
H9B	0.712888	0.224016	0.895087	0.031
C21	0.7023(2)	0.79712(18)	0.32768(13)	0.0317(4)
C7	0.43299(16)	0.42716(15)	0.85690(11)	0.0191(3)
H7	0.472434	0.425750	0.918870	0.023
C13	0.17902(16)	0.63646(15)	0.87353(11)	0.0195(3)
H13	0.115765	0.576817	0.878834	0.023
C1	0.42202(16)	0.74787(15)	0.66168(11)	0.0207(3)
C6	0.42209(16)	0.69136(14)	0.76494(11)	0.0187(3)
C11	0.44507(18)	0.17421(15)	0.92875(12)	0.0258(3)
H11A	0.484589	0.163513	0.992563	0.031
H11B	0.384207	0.111799	0.941182	0.031
C4	0.58520(18)	0.82255(17)	0.76724(13)	0.0289(4)
H4	0.641389	0.848460	0.803780	0.035
C14	0.08433(18)	0.78527(16)	0.82528(13)	0.0283(4)
H14A	0.145611	0.846646	0.815983	0.034
H14B	0.054567	0.792980	0.759326	0.034

C2	0.50088(19)	0.83887(16)	0.61083(12)	0.0277(4)
H2	0.499104	0.873745	0.540922	0.033
C8	0.56898(17)	0.38096(16)	0.79090(12)	0.0239(3)
H8A	0.533667	0.390931	0.725949	0.029
H8B	0.630515	0.442509	0.779904	0.029
C12	0.34632(17)	0.32279(15)	0.88670(12)	0.0227(3)
H12A	0.268703	0.346659	0.936686	0.027
H12B	0.295500	0.329686	0.828191	0.027
C18	0.21450(17)	0.62123(17)	0.97916(11)	0.0246(3)
H18A	0.282013	0.674990	0.976376	0.029
H18B	0.267385	0.523615	1.012159	0.029
C3	0.5816(2)	0.87697(18)	0.66463(13)	0.0322(4)
H3	0.634416	0.940047	0.631811	0.039
C17	0.07419(18)	0.67002(18)	1.04090(13)	0.0300(4)
H17A	0.011974	0.609304	1.050786	0.036
H17B	0.103829	0.663677	1.106544	0.036
C15	−0.05555(19)	0.83311(18)	0.88641(15)	0.0368(4)
H15A	−0.109428	0.930554	0.853664	0.044
H15B	−0.122331	0.778353	0.890010	0.044
C10	0.57490(17)	0.13370(16)	0.85843(12)	0.0253(3)
H10A	0.638989	0.039052	0.889285	0.030
H10B	0.536078	0.135579	0.796852	0.030
C23	0.85184(19)	0.54141(19)	0.63152(14)	0.0344(4)
C22	0.7553(2)	0.9009(2)	0.25275(14)	0.0401(4)
H22A	0.696712	0.933689	0.193179	0.060
H22B	0.860462	0.860594	0.236809	0.060
H22C	0.744144	0.978005	0.278011	0.060
C16	−0.0165(2)	0.81764(19)	0.99019(15)	0.0371(4)

H16A	−0.109162	0.844253	1.029169	0.045
H16B	0.041508	0.879783	0.987061	0.045
B1	0.32711(18)	0.58379(17)	0.80807(12)	0.0185(3)
C24	0.9395(3)	0.5717(3)	0.69283(18)	0.0548(6)
H24A	0.954670	0.660772	0.658897	0.082
H24B	1.036254	0.500041	0.704968	0.082
H24C	0.886424	0.574723	0.755845	0.082
C19	0.1723(3)	0.8577(3)	0.45522(15)	0.0433(6)
H19A	0.264223	0.811326	0.423205	0.052
H19B	0.170821	0.953100	0.445165	0.052
C20	0.0414(3)	0.8596(3)	0.40948(18)	0.0527(7)
H20A	0.040516	0.911630	0.339429	0.079
H20B	−0.049521	0.903169	0.442497	0.079
H20C	0.045952	0.765337	0.416051	0.079
Li1	0.6113(3)	0.5543(3)	0.4934(2)	0.0298(6)
C19A	0.094(2)	0.809(2)	0.4788(14)	0.0433(6)
H19C	−0.013765	0.846274	0.493607	0.052
H19D	0.110396	0.720140	0.466464	0.052
C20A	0.134(3)	0.903(3)	0.3907(17)	0.0527(7)
H20D	0.112652	0.882859	0.332620	0.079
H20E	0.240220	0.891216	0.390965	0.079
H20F	0.075768	0.997065	0.388651	0.079

Table S8. Bond lengths for [Li(MeCN)$_2$][**3**].

Atom–Atom	Length [Å]
P1–O2	1.5249(11)
P1–O1	1.5001(12)
P1–O3	1.5958(12)
P1–C1	1.7812(15)
O2–B1	1.6236(18)
O1–Li1$^{\#1}$	1.940(3)
O1–Li1	1.931(3)
O3–C19	1.448(2)
O3–C19A	1.38(2)
N2–C21	1.142(2)
N2–Li1	2.071(3)
N1–C23	1.136(2)
N1–Li1	2.067(3)
C5–H5	0.9500
C5–C6	1.406(2)
C5–C4	1.391(2)
C9–H9A	0.9900
C9–H9B	0.9900
C9–C8	1.534(2)
C9–C10	1.525(2)
C21–C22	1.461(2)
C7–H7	1.0000
C7–C8	1.542(2)
C7–C12	1.536(2)
C7–B1	1.632(2)
C13–H13	1.0000
C13–C14	1.538(2)
C13–C18	1.536(2)
C13–B1	1.631(2)
C1–C6	1.407(2)
C1–C2	1.399(2)
C6–B1	1.628(2)
C11–H11A	0.9900
C11–H11B	0.9900
C11–C12	1.533(2)
C11–C10	1.528(2)
C4–H4	0.9500
C4–C3	1.397(3)
C14–H14A	0.9900
C14–H14B	0.9900
C14–C15	1.530(2)
C2–H2	0.9500
C2–C3	1.384(2)
C8–H8A	0.9900
C8–H8B	0.9900
C12–H12A	0.9900
C12–H12B	0.9900
C18–H18A	0.9900
C18–H18B	0.9900
C18–C17	1.540(2)
C3–H3	0.9500
C17–H17A	0.9900
C17–H17B	0.9900

C17–C16	1.525(3)
C15–H15A	0.9900
C15–H15B	0.9900
C15–C16	1.521(3)
C10–H10A	0.9900
C10–H10B	0.9900
C23–C24	1.458(3)
C22–H22A	0.9800
C22–H22B	0.9800
C22–H22C	0.9800
C16–H16A	0.9900
C16–H16B	0.9900
C24–H24A	0.9800
C24–H24B	0.9800
C24–H24C	0.9800
C19–H19A	0.9900
C19–H19B	0.9900
C19–C20	1.472(3)
C20–H20A	0.9800
C20–H20B	0.9800
C20–H20C	0.9800
Li1–Li1$^{\#1}$	2.706(6)
C19A–H19C	0.9900
C19A–H19D	0.9900
C19A–C20A	1.44(3)
C20A–H20D	0.9800
C20A–H20E	0.9800
C20A–H20F	0.9800

Table 9. Bond angles for [Li(MeCN)$_2$][**3**].

Atom–Atom–Atom	Bond Angle [°]
O2–P1–O3	105.60(6)
O2–P1–C1	100.62(6)
O1–P1–O2	116.66(6)
O1–P1–O3	108.60(7)
O1–P1–C1	113.89(7)
O3–P1–C1	111.03(7)
P1–O2–B1	115.14(9)
P1–O1–Li1	135.36(11)
P1–O1–Li1$^{\#1}$	130.85(11)
Li1–O1–Li1$^{\#1}$	88.71(13)
C19–O3–P1	121.72(13)
C19A–O3–P1	135.5(10)
C21–N2–Li1	173.10(18)
C23–N1–Li1	158.67(18)
C6–C5–H5	119.5
C4–C5–H5	119.5
C4–C5–C6	121.09(14)
H9A–C9–H9B	108.0
C8–C9–H9A	109.4
C8–C9–H9B	109.4
C10–C9–H9A	109.4
C10–C9–H9B	109.4
C10–C9–C8	110.96(13)
N2–C21–C22	179.45(19)
C8–C7–H7	106.9
C8–C7–B1	113.13(12)
C12–C7–H7	106.9
C12–C7–C8	110.13(12)
C12–C7–B1	112.59(12)
B1–C7–H7	106.9
C14–C13–H13	107.0
C14–C13–B1	113.20(12)
C18–C13–H13	107.0
C18–C13–C14	109.17(13)
C18–C13–B1	113.02(12)
B1–C13–H13	107.0
C6–C1–P1	108.13(11)
C2–C1–P1	128.39(12)
C2–C1–C6	123.32(14)
C5–C6–C1	116.30(14)
C5–C6–B1	128.69(13)
C1–C6–B1	114.96(13)
H11A–C11–H11B	107.9
C12–C11–H11A	109.3
C12–C11–H11B	109.3
C10–C11–H11A	109.3
C10–C11–H11B	109.3
C10–C11–C12	111.77(13)
C5–C4–H4	119.6
C5–C4–C3	120.83(15)
C3–C4–H4	119.6
C13–C14–H14A	109.0
C13–C14–H14B	109.0

H14A–C14–H14B	107.8
C15–C14–C13	112.85(14)
C15–C14–H14A	109.0
C15–C14–H14B	109.0
C1–C2–H2	120.7
C3–C2–C1	118.54(15)
C3–C2–H2	120.7
C9–C8–C7	113.69(13)
C9–C8–H8A	108.8
C9–C8–H8B	108.8
C7–C8–H8A	108.8
C7–C8–H8B	108.8
H8A–C8–H8B	107.7
C7–C12–H12A	108.9
C7–C12–H12B	108.9
C11–C12–C7	113.37(12)
C11–C12–H12A	108.9
C11–C12–H12B	108.9
H12A–C12–H12B	107.7
C13–C18–H18A	109.0
C13–C18–H18B	109.0
C13–C18–C17	112.80(13)
H18A–C18–H18B	107.8
C17–C18–H18A	109.0
C17–C18–H18B	109.0
C4–C3–H3	120.0
C2–C3–C4	119.91(15)
C2–C3–H3	120.0
C18–C17–H17A	109.4
C18–C17–H17B	109.4
H17A–C17–H17B	108.0
C16–C17–C18	111.19(14)
C16–C17–H17A	109.4
C16–C17–H17B	109.4
C14–C15–H15A	109.4
C14–C15–H15B	109.4
H15A–C15–H15B	108.0
C16–C15–C14	111.21(14)
C16–C15–H15A	109.4
C16–C15–H15B	109.4
C9–C10–C11	110.37(13)
C9–C10–H10A	109.6
C9–C10–H10B	109.6
C11–C10–H10A	109.6
C11–C10–H10B	109.6
H10A–C10–H10B	108.1
N1–C23–C24	178.9(2)
C21–C22–H22A	109.5
C21–C22–H22B	109.5
C21–C22–H22C	109.5
H22A–C22–H22B	109.5
H22A–C22–H22C	109.5
H22B–C22–H22C	109.5
C17–C16–H16A	109.5
C17–C16–H16B	109.5
C15–C16–C17	110.83(14)

C15–C16–H16A	109.5
C15–C16–H16B	109.5
H16A–C16–H16B	108.1
O2–B1–C7	106.91(11)
O2–B1–C13	104.98(11)
O2–B1–C6	100.91(11)
C13–B1–C7	115.15(12)
C6–B1–C7	112.45(12)
C6–B1–C13	114.74(12)
C23–C24–H24A	109.5
C23–C24–H24B	109.5
C23–C24–H24C	109.5
H24A–C24–H24B	109.5
H24A–C24–H24C	109.5
H24B–C24–H24C	109.5
O3–C19–H19A	109.5
O3–C19–H19B	109.5
O3–C19–C20	110.69(19)
H19A–C19–H19B	108.1
C20–C19–H19A	109.5
C20–C19–H19B	109.5
C19–C20–H20A	109.5
C19–C20–H20B	109.5
C19–C20–H20C	109.5
H20A–C20–H20B	109.5
H20A–C20–H20C	109.5
H20B–C20–H20C	109.5
O1–Li1–O1$^{\#1}$	91.29(13)
O1$^{\#1}$–Li1–N2	120.65(15)
O1–Li1–N2	113.40(15)
O1–Li1–N1	120.51(15)
O1$^{\#1}$–Li1–N1	118.48(16)
O1–Li1–Li1$^{\#1}$	45.78(9)
O1$^{\#1}$–Li1–Li1$^{\#1}$	45.51(9)
N2–Li1–Li1$^{\#1}$	130.45(19)
N1–Li1–N2	94.72(13)
N1–Li1–Li1$^{\#1}$	134.75(19)
O3–C19A–H19C	108.9
O3–C19A–H19D	108.9
O3–C19A–C20A	113.4(19)
H19C–C19A–H19D	107.7
C20A–C19A–H19C	108.9
C20A–C19A–H19D	108.9
C19A–C20A–H20D	109.5
C19A–C20A–H20E	109.5
C19A–C20A–H20F	109.5
H20D–C20A–H20E	109.5
H20D–C20A–H20F	109.5
H20E–C20A–H20F	109.5

8. DFT calculations modeling bond lengths of 2a and [3]

Geometry and energy minimizations were performed using Gaussian '16[i] software packages. DFT calculations (B3LYP-D3(BJ)/6-311++G(2d,p)) on model neutral and anion model compounds were conducted, and minimized structures confirmed by the absence of any imaginary frequency.[ii]

[i] Gaussian 16, Revision C.01, Frisch, M. J.; Trucks, G. W.; Schlegel, H. B.; Scuseria, G. E.; Robb, M. A.; Cheeseman, J. R.; Scalmani, G.; Barone, V.; Petersson, G. A.; Nakatsuji, H.; Li, X.; Caricato, M.; Marenich, A. V.; Bloino, J.; Janesko, B. G.; Gomperts, R.; Mennucci, B.; Hratchian, H. P.; Ortiz, J. V.; Izmaylov, A. F.; Sonnenberg, J. L.; Williams-Young, D.; Ding, F.; Lipparini, F.; Egidi, F.; Goings, J.; Peng, B.; Petrone, A.; Henderson, T.; Ranasinghe, D.; Zakrzewski, V. G.; Gao, J.; Rega, N.; Zheng, G.; Liang, W.; Hada, M.; Ehara, M.; Toyota, K.; Fukuda, R.; Hasegawa, J.; Ishida, M.; Nakajima, T.; Honda, Y.; Kitao, O.; Nakai, H.; Vreven, T.; Throssell, K.; Montgomery, J. A., Jr.; Peralta, J. E.; Ogliaro, F.; Bearpark, M. J.; Heyd, J. J.; Brothers, E. N.; Kudin, K. N.; Staroverov, V. N.; Keith, T. A.; Kobayashi, R.; Normand, J.; Raghavachari, K.; Rendell, A. P.; Burant, J. C.; Iyengar, S. S.; Tomasi, J.; Cossi, M.; Millam, J. M.; Klene, M.; Adamo, C.; Cammi, R.; Ochterski, J. W.; Martin, R. L.; Morokuma, K.; Farkas, O.; Foresman, J. B.; Fox, D. J. Gaussian, Inc., Wallingford CT, 2016. Gaussian 16, Revision C.01; Gaussian, Inc.: Wallingford CT, 2016. [ii]Schröder, H.; Creon, A.; Schwabe, T. Reformulation of the D3(Becke–Johnson) Dispersion Correction without Resorting to Higher than C6 Dispersion Coefficients. *J. Chem. Theory Comput.* **2015**, *11* (7), 3163-3170. DOI: 10.1021/acs.jctc.5b00400.

Anion

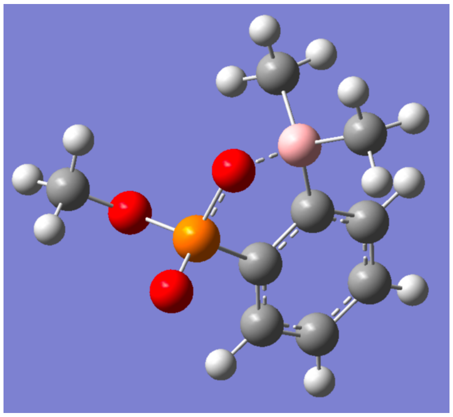

File Type	.log	
Calculation Type	FREQ	
Calculation Method	RB3LYP	
Formula	$C_9H_{13}BO_3P$	
Basis Set	6-311++G(2d,p)	
Charge	-1	
Spin	Singlet	
Solvation	None	
E(RB3LYP)	-943.146163	Hartree
RMS Gradient Norm	0.000029	Hartree/Bohr
Imaginary Freq	0	
Dipole Moment	3.997966	Debye
Polarizability (α)	162.929333	a.u.
Point Group	C1	
Molecular Mass	211.069537	amu

Symbol	X	Y	Z
C	0.5170030	-0.8169020	0.1480860
C	3.2153020	-0.9902110	-0.3731140
C	1.2295070	0.3719950	-0.0632140
C	1.1185990	-2.0709040	0.1135860
C	2.4804990	-2.1578090	-0.1508140
C	2.5977060	0.2560910	-0.3286140
H	0.5330960	-2.9660030	0.2937860
H	2.9720960	-3.1242100	-0.1821140
H	3.1907090	1.1504890	-0.4984140
H	4.2796020	-1.0604140	-0.5777140
P	-1.2029960	-0.4168970	0.4756860
O	-1.0815910	1.1359020	0.5102860
O	-1.9060970	-0.8115950	-0.9563140
O	-1.9197980	-1.0844950	1.5919860
C	-3.3142970	-0.6284910	-1.0473140
H	-3.6286980	-1.0343900	-2.0101140
H	-3.5697930	0.4351100	-1.0050140
B	0.3132110	1.7113980	0.0445860
H	-3.8267980	-1.1544890	-0.2378140
C	0.1106130	2.4260990	-1.4026140
H	-0.5798840	3.2763010	-1.3301140
H	-0.2947890	1.7335000	-2.1493140
H	1.0600140	2.8117960	-1.7969140
C	0.8318140	2.7331970	1.1938860
H	0.9074120	2.2461960	2.1729860
H	0.1516160	3.5873990	1.3028860
H	1.8237150	3.1382930	0.9527860

Neutral

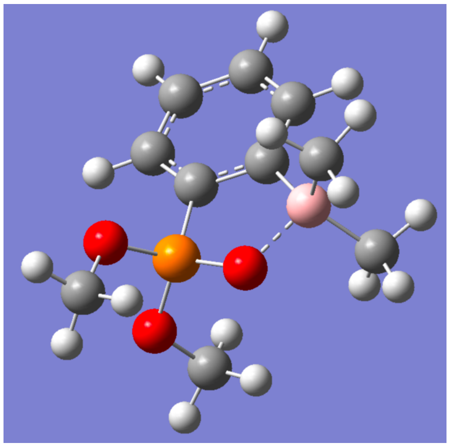

File Type	.log	
Calculation Type	FREQ	
Calculation Method	RB3LYP	
Formula	$C_{10}H_{16}BO_3P$	
Basis Set	6-311++G(2d,p)	
Charge	0	
Spin	Singlet	
Solvation	None	
E(RB3LYP)	-982.970260	Hartree
RMS Gradient Norm	0.000012	Hartree/Bohr
Imaginary Freq	0	
Dipole Moment	4.410010	Debye
Polarizability (α)	163.879000	a.u.
Point Group	C1	
Molecular Mass	226.093012	amu

Symbol	X	Y	Z
C	0.5764950	-0.8159020	-0.1144320
C	3.2839160	-1.2110670	-0.1571670
C	1.4038970	0.3191360	-0.1153140
C	1.0586390	-2.1263400	-0.1309950
C	2.4289180	-2.3191130	-0.1533800
C	2.7830670	0.0835920	-0.1383150
H	0.3799250	-2.9709160	-0.1304200
H	2.8387550	-3.3211660	-0.1692720
H	3.4701900	0.9223770	-0.1418390
H	4.3560500	-1.3711140	-0.1751150
P	-1.0878120	-0.2582640	-0.0611710
O	-0.9632310	1.2530450	-0.0288060
O	-1.9014730	-0.8519090	-1.2776730
O	-1.9362740	-0.8054030	1.1730920
C	-1.5857480	-0.3405470	2.4976020
H	-2.3245340	-0.7682170	3.1697190
H	-1.6191110	0.7471790	2.5320770
C	-3.2620890	-0.4202630	-1.5166490
H	-3.5578800	-0.8773680	-2.4565950
H	-3.2973130	0.6657140	-1.5991770
B	0.6562820	1.7534250	-0.0885930
H	-0.5885890	-0.6925590	2.7640950
H	-3.9068530	-0.7642240	-0.7089260
C	0.7992880	2.5829150	-1.4535840
H	0.1747280	3.4819730	-1.4458690
H	0.5293750	1.9956070	-2.3376730
H	1.8348490	2.9131220	-1.5948740
C	0.8911570	2.5799730	1.2674250
H	0.7167850	1.9863630	2.1717290
H	0.2460080	3.4629180	1.3174740
H	1.9259600	2.9369870	1.3211370

Mulliken Atomic Charges

Neutral

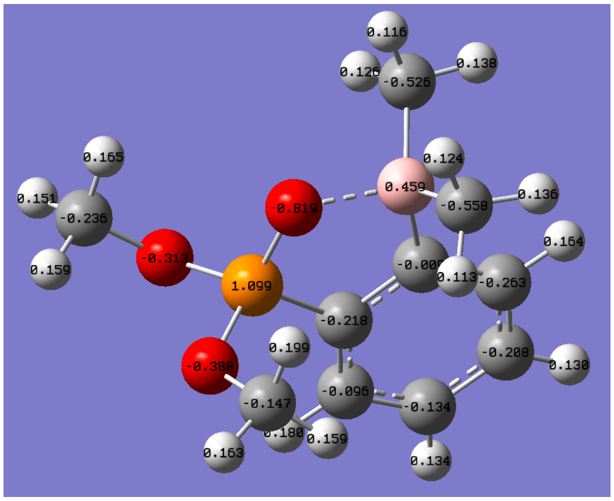

Anion

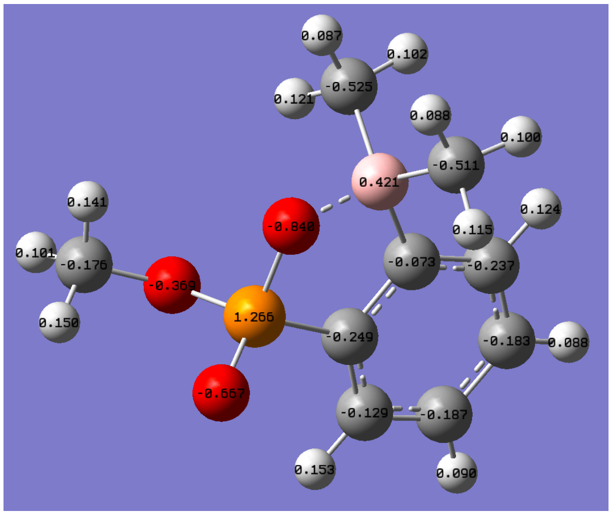

9. References

(1) Brown, H. C.; Basavaiah, D.; Kulkarni, S. U.; Bhat, N. G.; Prasad, J. V. N. V. Vinylic organoboranes. 9. A general stereospecific synthesis of (Z)- and (E)-disubstituted alkenes via organoboranes. *J. Org. Chem.* **1988**, *53* (2), 239-246. DOI: 10.1021/jo00237a003.

(2) Xu, F.; Duke, O. M.; Rojas, D.; Eichelberger, H. M.; Kim, R. S.; Clark, T. B.; Watson, D. A. Arylphosphonate-Directed Ortho C–H Borylation: Rapid Entry into Highly-Substituted Phosphoarenes. *J. Am. Chem. Soc.* **2020**, *142* (28), 11988-11992. DOI: 10.1021/jacs.0c04159.

(3) Brown, H. C.; Basavaiah, D.; Kulkarni, S. U. A stereospecific synthesis of trisubstituted alkenes via hydridation of dialkylhaloboranes followed by hydroboration-iodination of internal alkynes. *J. Org. Chem.* **1982**, *47* (1), 171-173. DOI: 10.1021/jo00340a045.
